# Levofloxacin enhances 5-fluorouracil efficacy in colorectal cancer by activating the DDR-TP53-mitochondrial apoptosis axis

**DOI:** 10.1016/j.isci.2026.117500

**Published:** 2026-09-17

**Authors:** Chenmeng Huang, Chenchen Sun, Han Wang, Ying Wei, Yufei Zhang, Wenfeng Li, Aping Yue, Yajing Yang, Jiajia Wang, Jindan Guo, Zhengxi Hu, Jiangyang Chi, Yongli Fan

**Affiliations:** 1 Department of Oncology, The First Affiliated Hospital of Henan University, Kaifeng, Henan 475001, China; 2 School of Life Sciences, Hubei University, Wuhan, Hubei 430062, China; 3 Hubei Key Laboratory of Natural Medicinal Chemistry and Resource Evaluation, and Wuhan (China-Romania) Belt and Road Joint Laboratory on Traditional Chinese Medicine, School of Pharmacy, Tongji Medical College, Huazhong University of Science and Technology, Wuhan, Hubei 430030, China; 4 Shouzheng Pharma Biotechnology Co., Ltd, Wuhan, Hubei 430200, China; 5 Institute of Reproductive Health, Tongji Medical College, Huazhong University of Science and Technology, Wuhan, Hubei 430030, China; 6 Kaifeng Key Laboratory of Precision Diagnosis and Treatment for Oncology, Kaifeng, Henan 475001, China

**Keywords:** levofloxacin, 5-fluorouracil, colorectal cancer, apoptosis, DNA damage

## Abstract

5-Fluorouracil (5-FU)-based regimens are a cornerstone of colorectal cancer (CRC) chemotherapy. Levofloxacin (LVF), a widely used fluoroquinolone antibiotic, has been reported to exhibit anticancer properties. We investigated LVF’s ability to enhance CRC chemosensitivity to 5-FU in cell lines, subcutaneous xenografts (Balb/c-nude mice), and spontaneous intestinal tumors (Apc^min/+^ mice). LVF potentiated 5-FU-induced apoptosis *in vitro* and *in vivo*. Mechanistically, LVF induced mitochondrial dysfunction in CRC cells, marked by reactive oxygen species (ROS) accumulation, increased mitochondrial ROS, and loss of mitochondrial membrane potential. TP53 and downstream effectors NOXA and BBC3 mediate between LVF-induced mitochondrial damage and 5-FU sensitization. LVF-induced DNA damage response (DDR) and subsequent oxidative phosphorylation (OXPHOS) upregulation trigger mitochondrial damage and TP53 activation. Importantly, we confirmed that the canonical LVF target TOP2A and the reported LVF effector ribosomal S6 kinase 4 (RSK4) are dispensable for LVF-mediated sensitization. Thus, LVF acts as a non-canonical 5-FU chemosensitizer in *TP53*-WT CRC via DDR-TP53-mitochondrial apoptosis activation, supporting clinical repurposing.

## Highlights

• Levofloxacin sensitizes colorectal cancer cells to 5-FU via sequential pretreatment
• Levofloxacin triggers mitochondrial dysfunction via ROS, cytochrome *c*, and ΔΨm collapse
• Levofloxacin initiates the DDR-p53-NOXA/BBC3 axis to amplify apoptosis
• Levofloxacin acts via a redox mechanism independent of TOP2A and RSK4

## Graphical abstract

**Figure:**
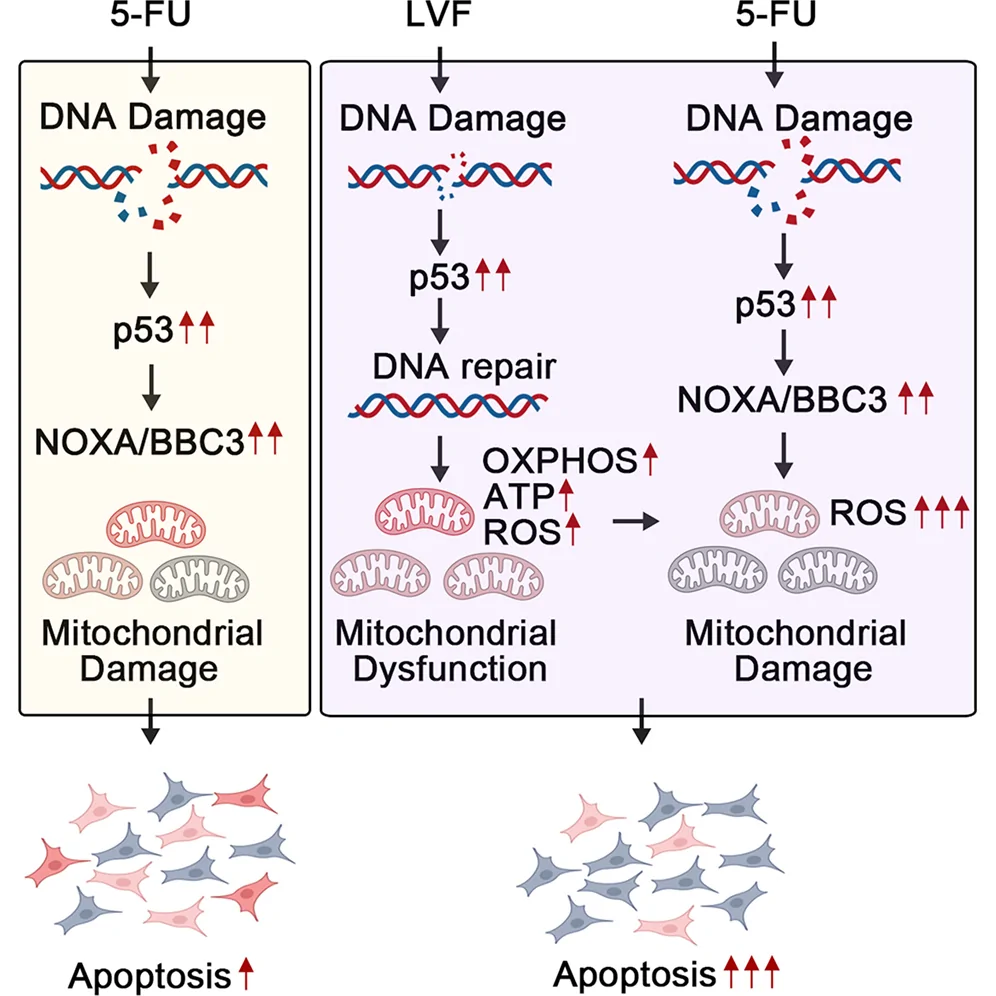

## Teaser

Biochemistry; Cell biology; Cancer

## Introduction

Colorectal cancer (CRC) ranks as the second leading cause of cancer-related death globally.^1^ For patients with metastatic disease, 5-fluorouracil (5-FU)-based regimens, such as folinic acid, 5-fluorouracil and oxaliplatin (FOLFOX) or folinic acid, 5-fluorouracil and irinotecan (FOLFIRI), remain the cornerstone of therapy.^2^ However, more than 50% of initially responding tumors develop chemoresistance, leading to disease relapse and dismal long-term survival.^3^^,^^4^ The antitumor activity of 5-FU is highly dependent on functional TP53 status: in *TP53*-wild-type (*TP53*-WT) CRC, 5-FU induces DNA damage and activates the *TP53* transcriptional program, thereby driving mitochondrial apoptosis^5^^,^^6^; conversely, in *TP53*-mutant tumors, loss of TP53 function abrogates activation of downstream pro-apoptotic pathways, resulting in therapeutic resistance.^7^^,^^8^^,^^9^ Consequently, downstream effectors, such as *NOXA* (*PMAIP1*) and *BBC3* (*PUMA*), direct *TP53* targets that drive mitochondrial outer-membrane permeabilization (MOMP), are expressed at levels below the threshold required for chemosensitivity.^10^^,^^11^ Compounding this, mitochondrial BCL-2 family members are frequently rewired in CRC, with MCL-1 (an anti-apoptotic BCL-2 family protein that sequesters pro-apoptotic BH3-only proteins) upregulation, BCL-2 (another anti-apoptotic protein) overexpression, and BAX (a pro-apoptotic effector protein that mediates MOMP) mitochondrial translocation defects that buffer MOMP.^12^^,^^13^ These observations point to a critical unmet need for pharmacological approaches that can potently activate the DDR-TP53-mitochondrial cascade and restore chemosensitivity in TP53-WT CRC.

Current strategies to enhance 5-FU efficacy have focused on either cytotoxic combinations (FOLFOX/FOLFIRI) or downstream apoptosis priming (e.g., BCL-2 inhibitors).^14^^,^^15^ However, these approaches often fail to address the root cause of incomplete DNA damage response (DDR) induction and inadequate TP53 transcriptional activation. Adding more agents to the regimen typically increases systemic toxicity without resolving the mitochondrial resistance barrier.^11^ This highlights an unmet need for clinically accessible compounds that can restore the DDR-mitochondrial checkpoint.

Fluoroquinolone antibiotics have long been known to target bacterial DNA gyrase, but accumulating evidence indicates off-target anticancer activity through mitochondrial reactive oxygen species (ROS) accumulation and DNA intercalation.^16^^,^^17^^,^^18^ Levofloxacin (LVF) exhibits cytostatic effects in multiple cancers, yet its precise mechanism in CRC remains unresolved.^19^^,^^20^^,^^21^ Recent studies demonstrate that fluoroquinolones induce mitochondrial DNA stress, leading to the release of cytoplasmic double-stranded DNA (dsDNA) and activation of innate immunity in a manner independent of gyrase inhibition.^19^^,^^22^ However, whether LVF induces nuclear DNA damage and triggers downstream signaling to activate the *TP53-NOXA/BBC3* axis in CRC remains unknown. Addressing this question is critical, as leveraging DDRs to reactivate *TP53*-dependent apoptosis represents a potential approach to overcome chemoresistance in TP53-WT CRC.^23^

In this study, we hypothesized that LVF sensitizes TP53-WT CRC cells to 5-FU by initiating a nuclear DNA damage-mediated *TP53*-*NOXA*/*BBC3* transcriptional cascade, thereby lowering the mitochondrial MOMP threshold. Using integrated pharmacogenomics, CRISPR-based node disruption, and *in vivo* validation through subcutaneous xenografts and Apc^min/+^ mouse models, we demonstrate that LVF triggers mitochondrial dysfunction and cytoplasmic dsDNA release, driving TP53-dependent NOXA/BBC3 upregulation and amplifying 5-FU-induced apoptosis. Furthermore, our data reveal that TOP2A, the canonical target of LVF, and ribosomal S6 kinase 4 (RSK4), a previously reported effector of LVF, are dispensable for this chemosensitization, establishing LVF as a non-canonical 5-FU sensitizer in *TP53*-WT CRC. These findings support the clinical development of LVF as a personalized therapeutic strategy for TP53-WT CRC to enhance 5-FU efficacy.

## Results

### LVF sensitizes CRC to 5-FU through enhanced apoptosis

**Figure 1:**
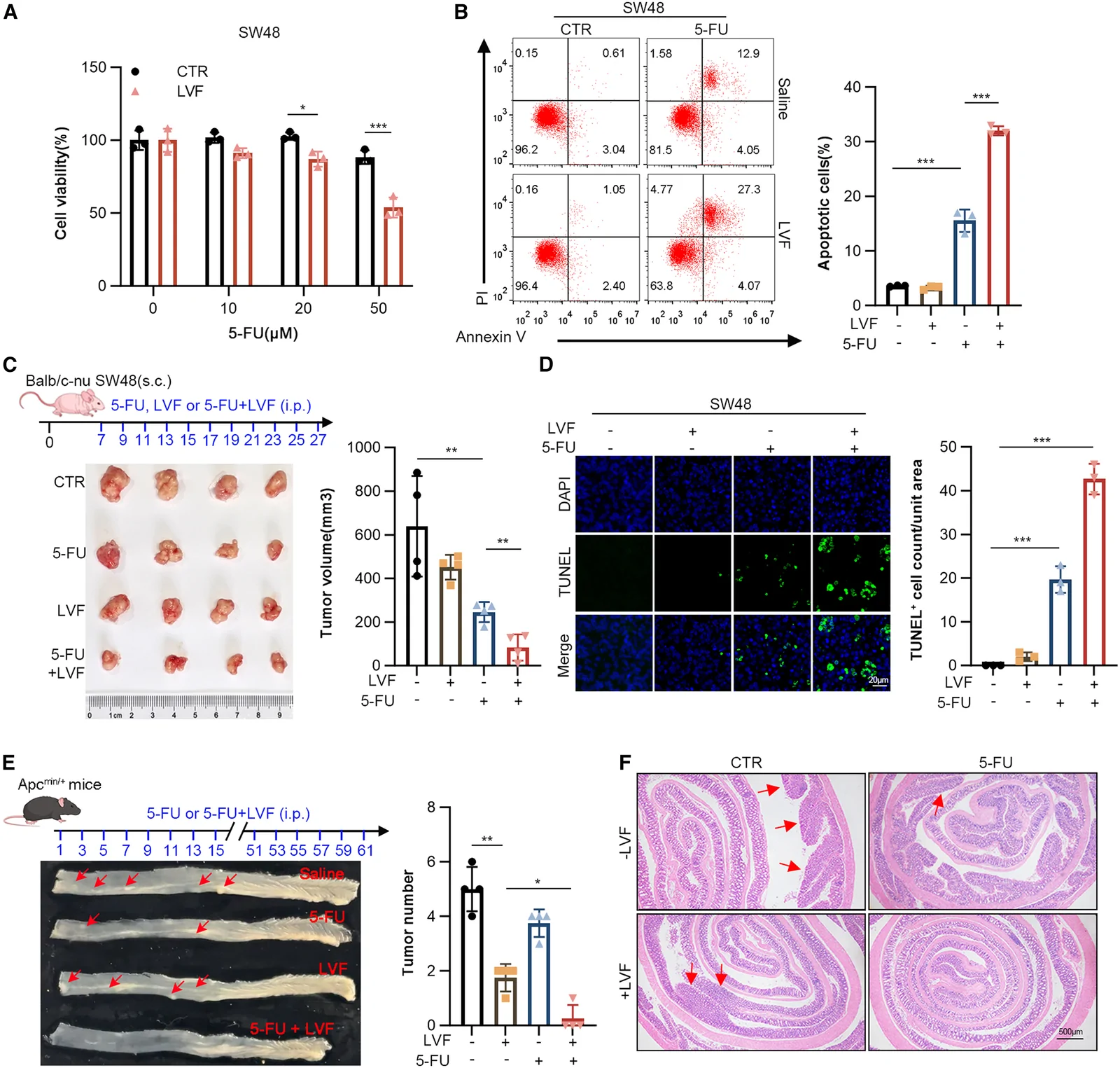
Effect of LVF combined with 5-FU on apoptosis in CRC (A) SW48 cells were pretreated with LVF for 48 h, followed by co-treatment with 5-FU and/or LVF for an additional 48 h. Cell viability was measured by CCK-8 assay. (B) SW48 cells were treated with 5-FU alone, LVF alone, or their combination. The apoptosis rate was determined by annexin V-FITC/PI staining and flow cytometry. (C) SW48 xenograft mice received combination therapy or 5-FU alone. Tumor volume was measured at the endpoint. (D) TUNEL staining was performed on SW48 xenograft tumor sections to detect apoptotic cells by immunofluorescence (green). Apoptotic cells were quantified. Scale bars, 20 μm. (E) Apc^min/+^ mice received combination therapy or 5-FU alone for 2 months. Intestinal polyps were counted. (F) H&E staining of intestinal tissues from Apc^min/+^ mice. Data are presented as means ± SD. For *in vitro* experiments, *n* = 3 independent experiments with three technical replicates each. For *in vivo* experiments, each group contained four mice. Statistical comparisons were performed using unpaired Student’s *t* test or one-way ANOVA. ∗*p* < 0.05, ∗∗*p* < 0.01, ∗∗∗*p* < 0.001.

To determine whether LVF could enhance 5-FU efficacy in CRC, SW48 and HCT116 cells were pretreated with LVF for 48 h before adding 5-FU. This sequential regimen markedly reduced viability compared to 5-FU alone, with consistent results observed in HCT116 cells (Figure 1A; Figure S1A). The 48-h half-maximal inhibitory concentration (IC50) of 5-FU in SW48 cells was 71.84 μM; accordingly, we selected 50 μM as a sub-IC50, moderate dose for subsequent experiments (Figure S1B). Subsequently, annexin V/phosphatidylinositol (PI) staining confirmed that LVF + 5-FU drove a marked increase in apoptotic cells in both cell lines (Figure 1B; Figure S1C), suggesting the combination pushes cells over an apoptotic threshold rather than simply slowing proliferation.

To test whether continuous LVF exposure is required for sensitization, we performed a washout experiment: cells were pretreated with LVF for 48 h, followed by LVF removal before 5-FU treatment. Cell counting kit-8 (CCK-8) assay 48 h after 5-FU addition showed similar viability between the washout and continuous LVF groups (Figure S1D), indicating that transient LVF exposure suffices to prime CRC cells for 5-FU-induced death. Colony formation assays revealed that both continuous and washout LVF pretreatment significantly reduced clonogenic survival relative to 5-FU alone (Figure S1E). Consistently, western blotting showed that the combination upregulated cleaved caspase-3, cleaved PARP, and BAX, and downregulated BCL-2 in SW48 and HCT116 cells (Figures S1F and S1G), confirming enhanced apoptosis.

In SW48 subcutaneous xenografts, the combination of LVF and 5-FU resulted in significantly smaller tumors, as measured by both volume and weight, than 5-FU monotherapy (Figure 1C; Figure S2A). Importantly, body weight remained stable across groups (Figure S2B), indicating the regimen was well tolerated by the mice. TUNEL staining of excised tumors confirmed increased apoptosis in the combination group (Figure 1D), consistent with our cell culture data.

To model the stepwise progression of CRC, we employed Apc^min/+^ mice, which develop spontaneous intestinal adenomas. After 2 months of treatment, mice treated with LVF + 5-FU developed significantly fewer polyps than those on 5-FU alone (Figure 1E), as confirmed by H&E staining (Figure 1F). No pathological changes were observed in major organs (Figure S3A), and body weight stayed stable (Figure S3B), indicating that the combination efficacy was not associated with systemic toxicity.

Together, these data demonstrate that LVF + 5-FU cooperates across cellular, xenograft, and genetic CRC models to promote apoptosis while maintaining a favorable safety profile, prompting investigation of the underlying mechanisms.

### LVF sensitizes CRC to 5-FU by triggering mitochondrial damage and ROS overproduction

**Figure 2:**
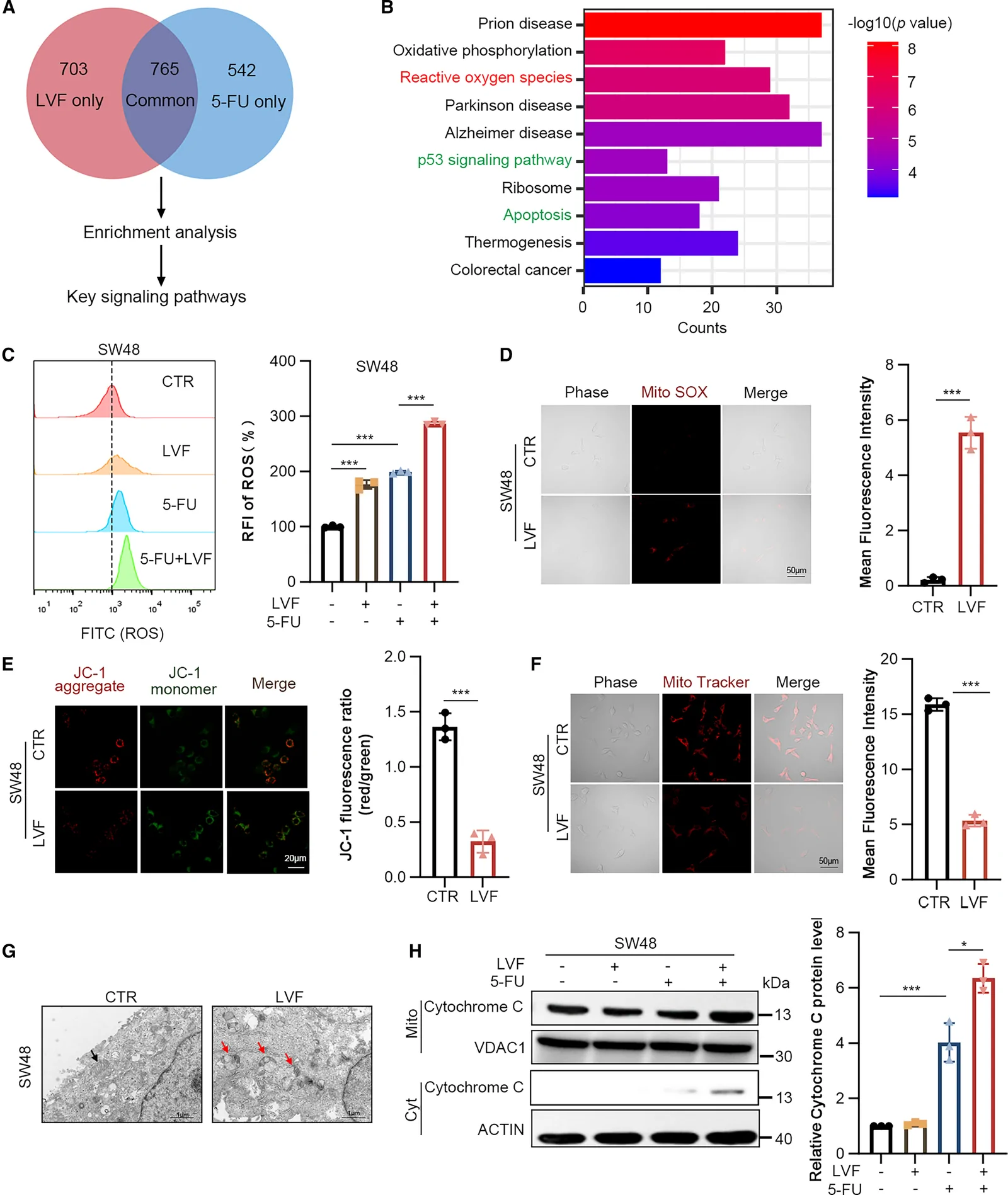
ROS generation and mitochondrial integrity in CRC cells treated with LVF combined with 5-FU (A) Transcriptomic analysis was performed on SW48 cells treated with LVF, and results were compared with gene expression data of 5-FU-treated HCT116 cells from GEO: GSE154146. Venn diagram shows 765 upregulated genes under both treatment conditions. (B) GSEA was conducted on the 765 co-upregulated genes. Enrichment of ROS metabolism-related pathways is shown. (C) Total intracellular ROS levels in SW48 cells were measured using the DCFH-DA probe and flow cytometry after treatment with LVF, 5-FU, or their combination. (D) Mitochondrial-specific ROS production in SW48 cells was evaluated using MitoSOX probe and immunofluorescence staining after combination treatment with LVF and 5-FU. Scale bars, 50 μm. (E) Mitochondrial membrane potential (ΔΨm) in SW48 cells was detected by JC-1 staining. Quantitative analysis of ΔΨm is shown. Scale bars, 20 μm. (F) Mitochondrial morphology in SW48 cells treated with combination therapy was observed by MitoTracker staining. Scale bars, 50 μm. (G) Mitochondrial ultrastructure of SW48 cells was examined by TEM. Scale bars, 1 μm. (H) Mitochondrial and cytoplasmic fractions were isolated from SW48 cells, and cytochrome *c* levels in the cytoplasm were detected by western blot analysis. Data are presented as means ± SD from three independent experiments with three technical replicates each. Statistical comparisons were performed using unpaired Student’s *t* test or one-way ANOVA. ∗*p* < 0.05, ∗∗*p* < 0.01, ∗∗∗*p* < 0.001.

To identify the molecular pathways underlying LVF-mediated 5-FU sensitization, we analyzed transcriptomic data from CRC cells treated with LVF or 5-FU. Comparison of our LVF-treated SW48 cells with a public HCT116 5-FU dataset (GEO: GSE154146) revealed 765 genes consistently upregulated by both treatments. Kyoto Encyclopedia of Genes and Genomes (KEGG) enrichment analysis of the 765 genes identified the ROS pathway as significantly enriched (Figure 2B; Figure S4), and this enrichment pattern’s tight correlation with mitochondrial dysfunction prompted functional investigation.

Consistent with this ROS-associated signature, we next examined whether it was reflected in functional redox changes. 2',7'-dichlorodihydrofluorescein diacetate (DCFH-DA) probing showed the combination drives a marked ROS surge in SW48 and HCT116 cells (Figure 2C; Figures S5 and S6). Further MitoSOX staining revealed marked elevation of mitochondrial ROS in both cell lines (Figure 2D; Figure S7A).

To dissect the mitochondrial mechanisms underlying LVF action, we examined membrane potential, morphology, and ultrastructure. JC-1 staining revealed loss of mitochondrial membrane potential (Figure 2E; Figure S7B), MitoTracker staining revealed fragmented networks (Figure 2F; Figure S7C), and transmission electron microscopy (TEM) revealed ultrastructural damage including cristae disruption (Figure 2G). To confirm the progression of mitochondrial apoptosis, subcellular fractionation followed by immunoblotting showed cytochrome *c* release into the cytoplasm (Figure 2H). Together, these LVF-induced mitochondrial alterations create a permissive state that renders CRC cells sensitive to subsequent 5-FU cytotoxicity.

### LVF sensitizes CRC to 5-FU through transcriptional upregulation of *NOXA* and *BBC3*

**Figure 3:**
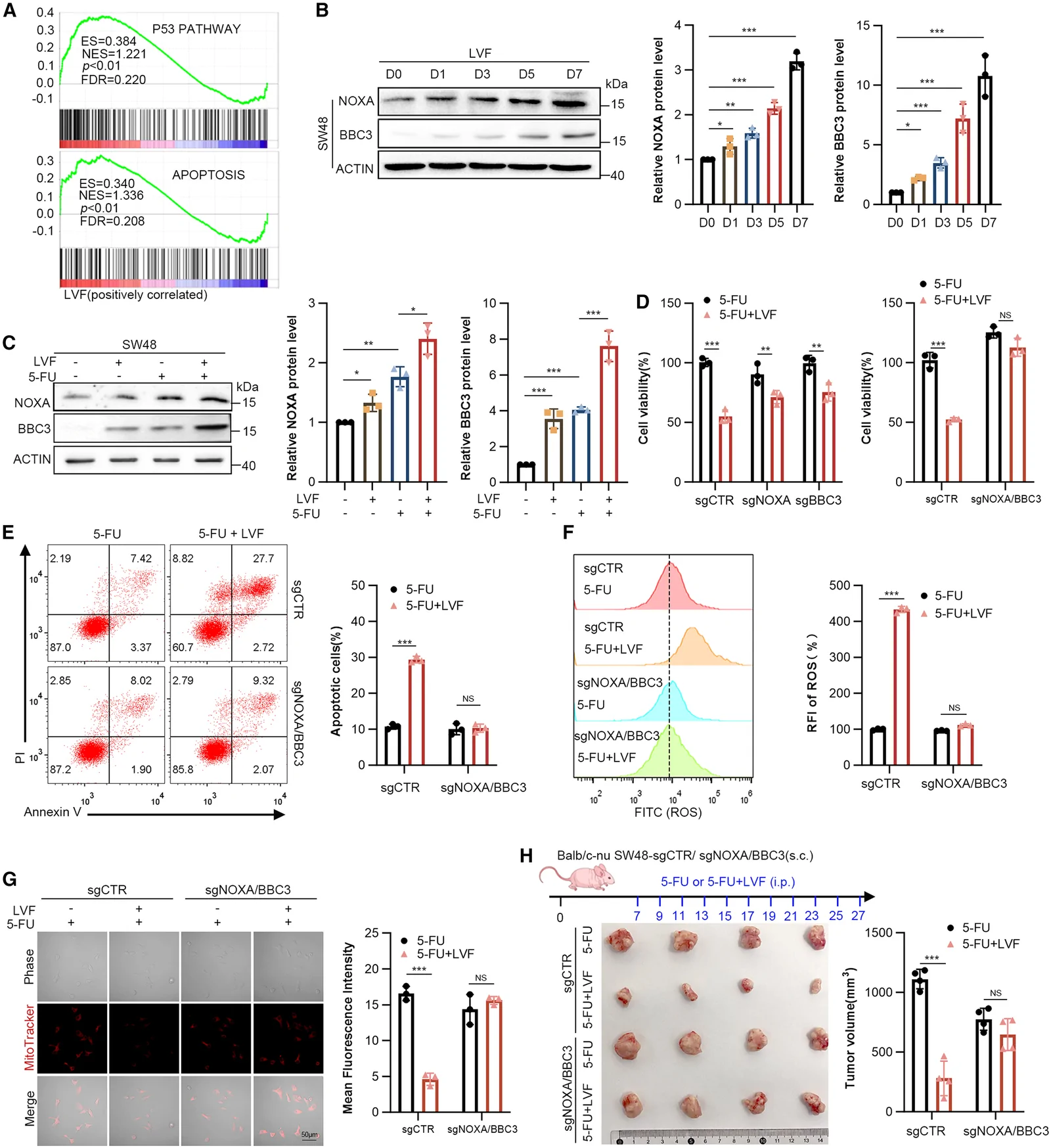
NOXA and BBC3 expression in CRC cells treated with LVF combined with 5-FU (A) GSEA plot showing enrichment of the TP53 and apoptosis pathways among co-upregulated genes. (B) Time-course western blot analysis of NOXA and BBC3 protein levels in SW48 cells treated with LVF from day 0 to day 7. (C) Western blot analysis of NOXA and BBC3 expression in SW48 cells treated with LVF, 5-FU, or their combination for 48 h. (D) (Left) Cell viability measured by CCK-8 assay in SW48 cells expressing control sgRNA (sgCTR), NOXA-knockout (sgNOXA), and BBC3-knockout (sgBBC3) cells treated with LVF, 5-FU, or LVF + 5-FU. (Right) Cell viability measured by CCK-8 assay in SW48-sgCTR and NOXA/BBC3-double-knockout (sgDKO) cells under the same treatment conditions. (E) Apoptosis analysis by annexin V-FITC/PI flow cytometry in SW48-sgCTR and SW48-sgDKO cells treated with 5-FU or LVF + 5-FU. (F) Intracellular ROS levels detected by DCFH-DA flow cytometry in SW48-sgCTR and SW48-sgDKO cells after treatment with LVF + 5-FU. (G) Mitochondrial morphology visualized by MitoTracker Red staining and confocal microscopy in SW48-sgCTR and SW48-sgDKO cells following LVF + 5-FU treatment. Scale bars, 50 μm. (H) Photographs of subcutaneous tumors in the left image and measured tumor volumes in the right image from SW48-sgCTR and SW48-sgDKO xenografts in nude mice treated with 5-FU or LVF + 5-FU for 4 weeks. Data are presented as means ± SD. For *in vitro* experiments, *n* = 3 independent experiments with three technical replicates each. For *in vivo* experiments, each group contained four mice. Statistical comparisons were performed using unpaired Student’s *t* test or one-way ANOVA. ∗*p* < 0.05, ∗∗*p* < 0.01, ∗∗∗*p* < 0.001.

As TP53 and apoptosis pathways were identified as top enriched signatures in our transcriptome analysis (Figure 3A; Figure S8), we next focused on two key pro-apoptotic genes, NOXA and BBC3. qPCR confirmed that both *NOXA* and *BBC3* were induced by the combination in SW48 and HCT116 cells (Figure S9), and time-course western blot analysis revealed their progressive upregulation over 7 days in SW48 (Figure 3B). A 48-h exposure to LVF + 5-FU further increased *NOXA* and *BBC3* expression compared with either drug alone (Figure 3C), with HCT116 showing similar responses (Figure S10).

To test whether these proteins are functionally required for LVF-induced 5-FU sensitization, we generated single and double knockouts (DKOs) in SW48. Notably, single-KO cells exhibited only partial resistance to the LVF + 5-FU combination, whereas DKO cells were completely resistant to the cytotoxic effects (Figures 3D and 3E). Annexin V/PI staining confirmed that apoptosis in the DKO group was reduced to 5-FU-alone levels (Figure 3E, lower), and all downstream markers we examined (ROS, mitochondrial morphology, membrane potential, and cytochrome *c* release) showed the same rescue (Figures 3F and 3G; Figures S12 and S13).

In SW48 xenografts, the LVF + 5-FU combination markedly suppressed tumor growth in control mice, whereas tumors with *NOXA*/*BBC3* DKO showed no enhanced response to LVF-mediated 5-FU chemosensitization (Figure 3H). At study endpoint, tumor weight and TUNEL staining confirmed that NOXA and BBC3 were essential for this enhanced cytotoxicity (Figures S14A–S14C). Collectively, these findings demonstrate that LVF sensitizes CRC to 5-FU by upregulating NOXA and BBC3, which are indispensable for initiating mitochondrial apoptosis and mediating the chemosensitization.

### TP53 is an essential upstream regulator of LVF-mediated 5-FU chemosensitization

**Figure 4:**
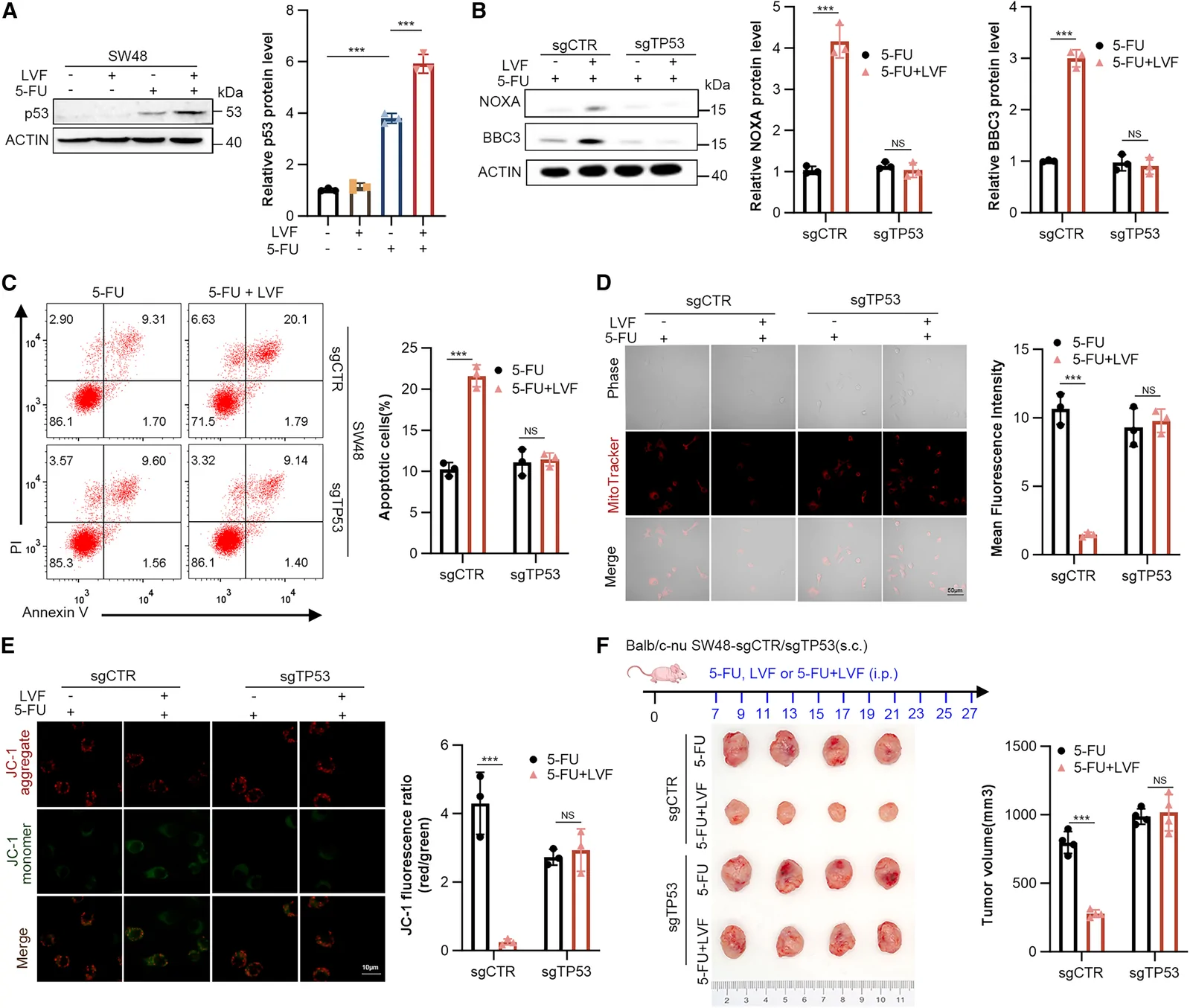
Role of TP53 in LVF combined with 5-FU-mediated sensitization in CRC cells (A) Western blot analysis of total p53 protein levels in SW48 cells treated with LVF, 5-FU, or LVF + 5-FU. (B) Western blot analysis of NOXA and BBC3 protein levels in *TP53*-knockout sgTP53 SW48 cells and control sgCTR cells treated with LVF. (C) Apoptosis analysis by annexin V/PI flow cytometry in control sgCTR and sgTP53 SW48 cells treated with 5-FU or LVF + 5-FU. (D) Mitochondrial morphology visualized by MitoTracker staining in control sgCTR and sgTP53 SW48 cells treated with 5-FU or LVF + 5-FU. Scale bars, 50 μm. (E) Mitochondrial membrane potential assessed by JC-1 staining in control sgCTR and sgTP53 SW48 cells treated with 5-FU or LVF + 5-FU. Scale bars, 10 μm. (F) Left: photographs of subcutaneous tumors from control sgCTR and sgTP53 SW48 xenografts in nude mice treated with 5-FU or LVF + 5-FU. Right: tumor volume measurements of the corresponding xenografts. Data are presented as means ± SD. For *in vitro* experiments, *n* = 3 independent experiments with three technical replicates each. For *in vivo* experiments, each group contained four mice. Statistical comparisons were performed using unpaired Student’s *t* test or one-way ANOVA. ∗*p* < 0.05, ∗∗*p* < 0.01, ∗∗∗*p* < 0.001.

Given that NOXA and BBC3 are direct transcriptional targets of TP53, we examined whether LVF potentiates 5-FU-induced TP53 activation. Indeed, the combination robustly increased TP53 protein levels in SW48 cells compared with either single agent (Figure 4A). To determine whether this upregulation was functionally required, we generated *TP53*-KO SW48 cells (Figure S15). Notably, ablation of *TP53* abolished the LVF-induced upregulation of NOXA and BBC3 (Figure 4B) and abrogated 5-FU chemosensitization, with the combination failing to enhance apoptosis beyond 5-FU alone in these cells (Figure 4C). Mitochondrial dysfunction mirrored this TP53 requirement, and both membrane-potential collapse and network fragmentation were prevented in *TP53*-KO cells (Figures 4D and 4E).

To further confirm the TP53 dependence of LVF-mediated 5-FU chemosensitization, we performed CCK-8 assays in *TP53*-mutant HT29 and SW480 cells. In these lines, the LVF + 5-FU combination exhibited an attenuated sensitization effect (Figure S16).

*In vivo* validation using SW48 xenografts revealed that *TP53* KO completely blocked LVF-mediated 5-FU sensitization. Parental tumors showed marked growth inhibition, whereas *TP53*-KO tumors remained resistant to combination therapy (Figure 4F). Analysis of endpoint tumor weight and TUNEL staining confirmed the TP53-dependency of LVF-mediated 5-FU chemosensitization (Figure S17). These data establish TP53 as an essential upstream mediator of LVF-mediated 5-FU chemosensitization, governing *NOXA*/*BBC3* transcription and thereby driving the ensuing mitochondrial apoptotic cascade.

### LVF sensitizes CRC to 5-FU efficacy by inducing nuclear DNA damage

**Figure 5:**
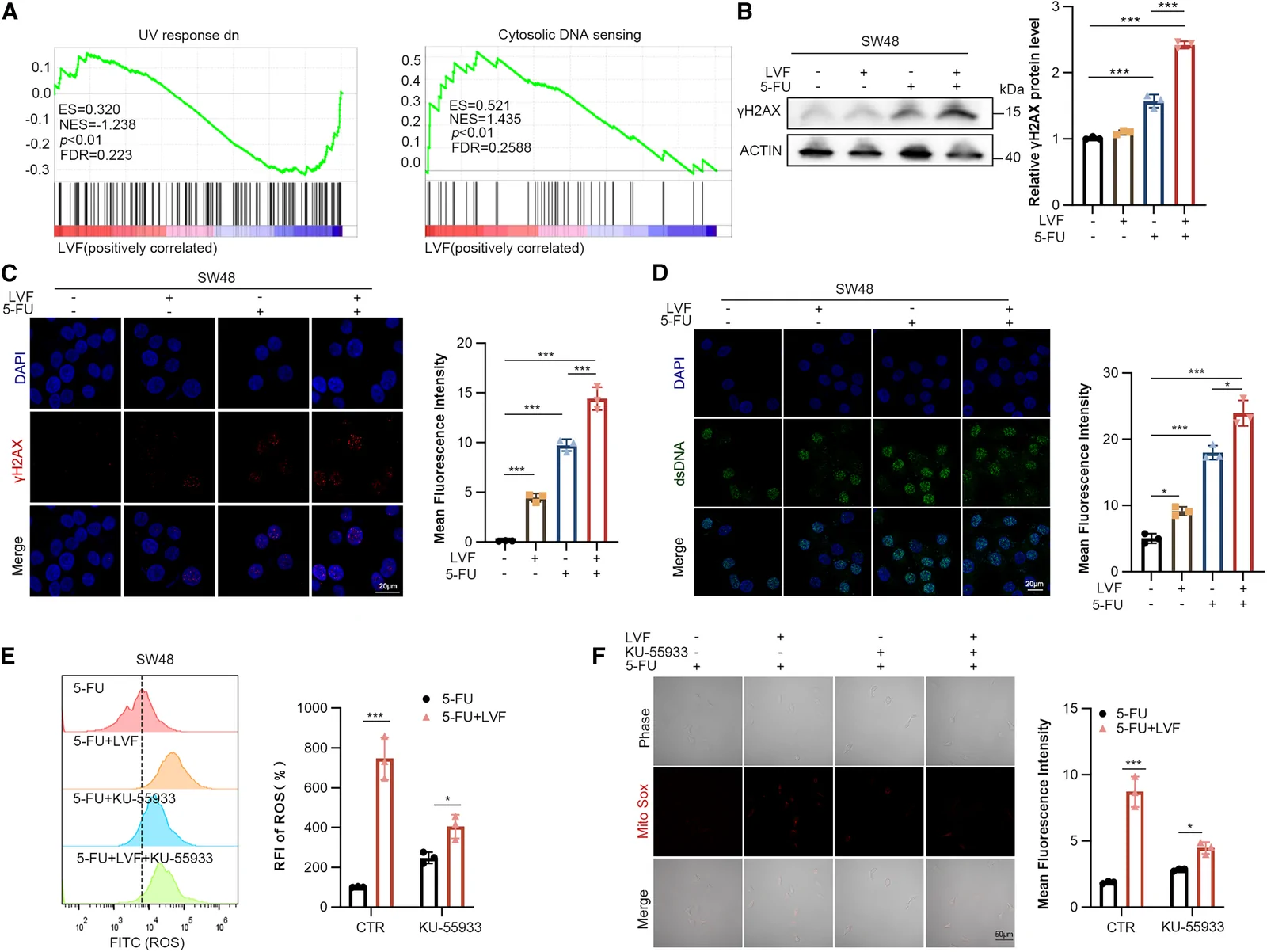
DNA stress in CRC cells treated with LVF combined with 5-FU (A) Gene set enrichment analysis (GSEA) of transcriptome data from LVF-treated SW48 cells, showing enrichment scores for “UV response” and “cytoplasmic DNA sensing” pathways. (B) Western blot analysis of γH2AX protein levels in SW48 cells treated with LVF, 5-FU, or LVF + 5-FU. (C) Immunofluorescence staining of γH2AX foci in SW48 cells treated with LVF, 5-FU, or LVF + 5-FU. Scale bars, 20 μm. (D) Immunofluorescence detection of cytoplasmic double-stranded DNA (dsDNA) in SW48 cells treated with LVF, 5-FU, or LVF + 5-FU. Scale bars, 20 μm. (E) Flow cytometry analysis of total intracellular ROS levels using DCFH-DA probe in SW48 cells treated with LVF + 5-FU in the presence or absence of KU-55933. (F) Mitochondrial ROS levels measured by MitoSOX staining in SW48 cells treated with LVF + 5-FU in the presence or absence of KU-55933. Scale bars, 50 μm. Data are presented as means ± SD from three independent experiments with three technical replicates each. Statistical comparisons were performed using unpaired Student’s *t* test or one-way ANOVA. ∗*p* < 0.05, ∗∗*p* < 0.01, ∗∗∗*p* < 0.001.

To identify mechanisms underlying LVF-induced activation of the *TP53*-*NOXA*/*BBC3* axis, we performed gene set enrichment analysis (GSEA) on transcriptomic data. GSEA of LVF-treated cells revealed strong enrichment in the UV response pathway and cytoplasmic DNA-sensing pathway (Figure 5A), consistent with LVF-induced DNA stress as an initiating event. Concordantly, LVF + 5-FU treatment markedly elevated γH2AX protein levels above either monotherapy (Figure 5B), confirming increased nuclear DNA damage. Immunofluorescence revealed pronounced intranuclear γH2AX foci (Figure 5C) concomitant with increased cytoplasmic dsDNA signals (Figure 5D), indicating that LVF + 5-FU induces genotoxic stress with potential crosstalk to cytosolic DNA sensing pathways.

We next sought to determine whether nuclear DDR activation links nuclear DNA damage to mitochondrial dysfunction by treating cells with KU-55933, a specific ATM inhibitor that blocks ATM-mediated DDR signaling. As expected, KU-55933 abolished the LVF + 5-FU combination induced accumulation of both cellular and mitochondrial ROS (Figures 5E and 5F), indicating that ATM-dependent DDR signaling is required for the downstream mitochondrial dysfunction and chemosensitization. Collectively, these results indicate that LVF initiates nuclear DNA damage, which activates ATM-dependent DDR signaling to drive mitochondrial dysfunction and cytoplasmic dsDNA release, thereby sensitizing CRC cells to 5-FU-induced apoptosis.

### DDR-dependent OXPHOS activation drives LVF-induced mitochondrial damage

**Figure 6:**
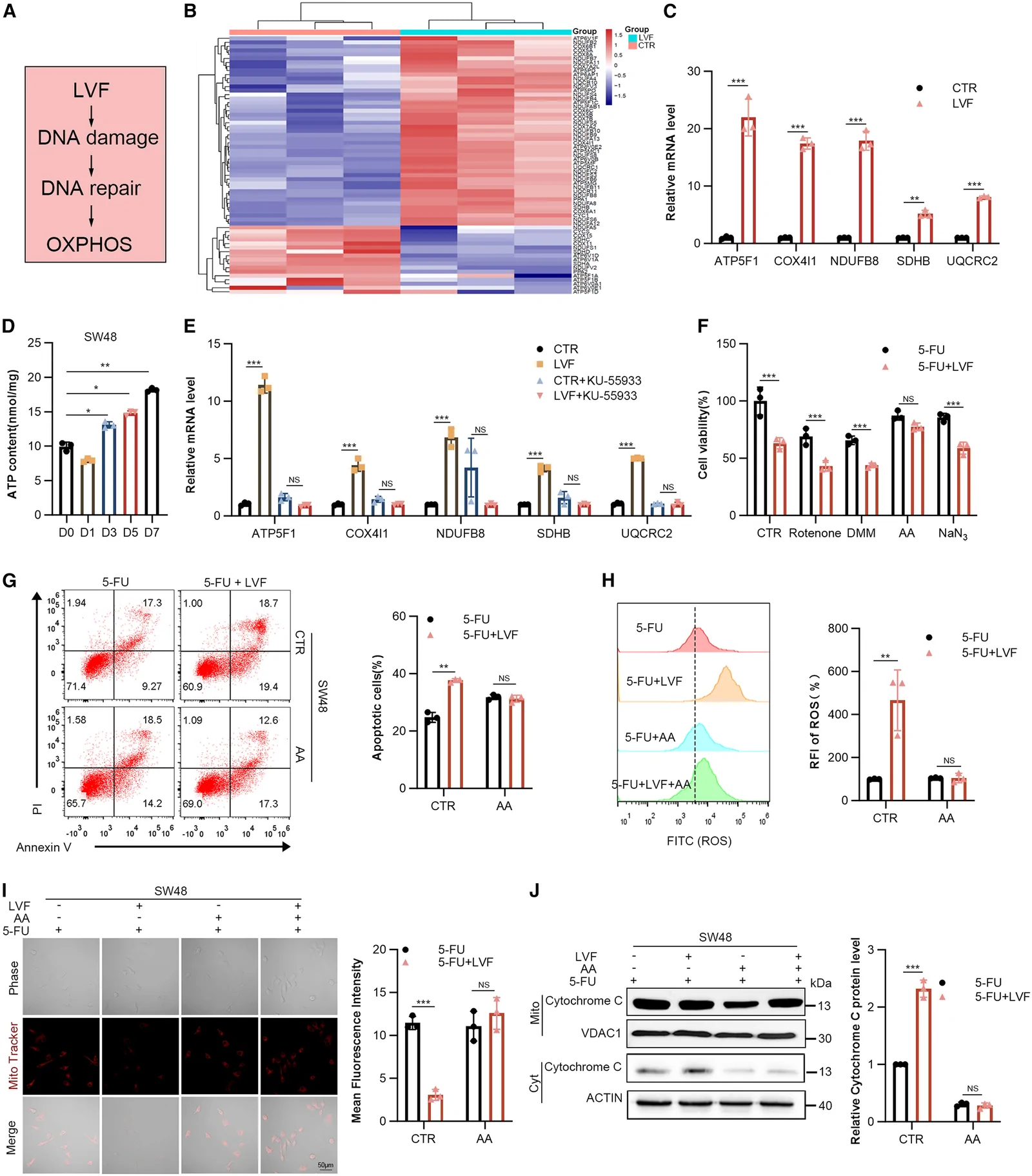
Role of OXPHOS in LVF combined with 5-FU treatment in CRC cells (A) Schematic diagram illustrating the mechanism of LVF-induced oxidative stress in CRC. (B) Heatmap of OXPHOS-related gene expression in SW48 cells after LVF treatment. (C) qPCR analysis of OXPHOS subunit mRNA levels in SW48 cells following LVF treatment. (D) Cellular ATP content in SW48 cells after LVF treatment over time. (E) qPCR analysis of OXPHOS subunit mRNA levels in SW48 cells treated with LVF with or without KU-55933. (F) Cell viability of SW48 cells treated with LVF + 5-FU in combination with various mitochondrial inhibitors. (G) Apoptosis analysis by annexin V/PI staining in SW48 cells treated with LVF + 5-FU with or without AA pretreatment. (H) Flow cytometry analysis of total intracellular ROS levels in SW48 cells treated with LVF + 5-FU with or without AA pretreatment. (I) Mitochondrial morphology and mass visualized by MitoTracker staining in SW48 cells treated with LVF + 5-FU with or without AA pretreatment. Scale bars, 50 μm. (J) Western blot analysis of cytochrome *c* release and VDAC1 loading control in cytoplasmic fractions of SW48 cells treated with LVF + 5-FU with or without AA pretreatment. Data are presented as means ± SD from three independent experiments with three technical replicates each. Statistical comparisons were performed using unpaired Student’s *t* test or one-way ANOVA. ∗*p* < 0.05, ∗∗*p* < 0.01, ∗∗∗*p* < 0.001.

Given that DDR activation rapidly activates oxidative phosphorylation (OXPHOS) to generate ATP required for DNA repair, we hypothesized that LVF-induced genotoxic stress might trigger such metabolic reprogramming (Figure 6A). Indeed, heatmap visualization of RNA sequencing (RNA-seq) data revealed robust induction of OXPHOS gene expression following LVF treatment in SW48 cells (Figure 6B), suggesting that LVF links DNA damage signaling with enhanced oxidative metabolism to meet bioenergetic demands. Consistent with the RNA-seq data, LVF upregulated key OXPHOS subunits (*ATP5F1*, *COX4I1*, *NDUFB8*, *SDHB*, and *UQCRC2*) and drove a progressive increase in ATP levels over the course of 7 days in SW48 cells (Figures 6C and 6D); HCT116 cells exhibited a similar time-dependent response (Figure S18).

To further explore the metabolic changes induced by LVF, we detected the lactate content in both cell supernatant and intracellular compartments. However, no obvious trend was observed in either cell supernatant lactate levels or intracellular lactate content following LVF treatment, indicating that LVF-induced metabolic reprogramming is mainly focused on OXPHOS activation rather than glycolytic pathway regulation (Figure S19). We next examined whether ATM inhibition by KU-55933 reverses the LVF-mediated induction of these OXPHOS genes and found that KU-55933 abrogated the upregulation of *ATP5F1*, *COX4I1*, *NDUFB8*, *SDHB*, and *UQCRC2* (Figure 6E).

As robust OXPHOS activation often generates mitochondrial ROS via respiratory chain leakage, we next investigated whether LVF-induced oxidative metabolism fuels the observed ROS-dependent damage.^24^ To test the functional requirement of respiratory chain complexes, we treated cells with a series of mitochondrial respiratory inhibitors targeting distinct complexes: rotenone (complex I), dimethyl malonate (DMM, complex II), antimycin A (AA, complex III), and sodium azide (NaN₃, complex IV). Cell viability assays showed that only the complex III inhibitor AA effectively blocked the cytotoxicity induced by LVF + 5-FU co-treatment (Figure 6F) and reduced apoptotic cell death (Figure 6G). Flow cytometry analysis revealed that AA attenuated the accumulation of total intracellular ROS induced by LVF + 5-FU treatment (Figure 6H). MitoTracker imaging showed that AA pretreatment restored the mitochondrial network morphology disrupted by LVF + 5-FU (Figure 6I). Western blot showed that AA or mitoquinone mesylate (Mito-Q) co-treatment did not block LVF + 5-FU-induced upregulation of γH2AX and p53, but markedly suppressed *NOXA* and *BBC3* induction in SW48 cells (Figure S20). Western blot analysis of cytoplasmic fractions further confirmed that AA blocked cytochrome c release from mitochondria into the cytosol in LVF + 5-FU treated SW48 cells, with VDAC1 serving as the loading control (Figure 6J).

Collectively, these data establish that DDR-dependent OXPHOS activation drives mitochondrial damage to mediate LVF-induced 5-FU chemosensitization.

### Canonical TOP2A and RSK4 are dispensable for LVF-mediated 5-FU chemosensitization

While TOP2A is widely recognized as the canonical target of fluoroquinolones, whether it mediates LVF-induced 5-FU sensitization remains unknown; we therefore examined TOP2A together with RSK4, a reported mediator of LVF action in other malignancies.^25^

**Figure 7:**
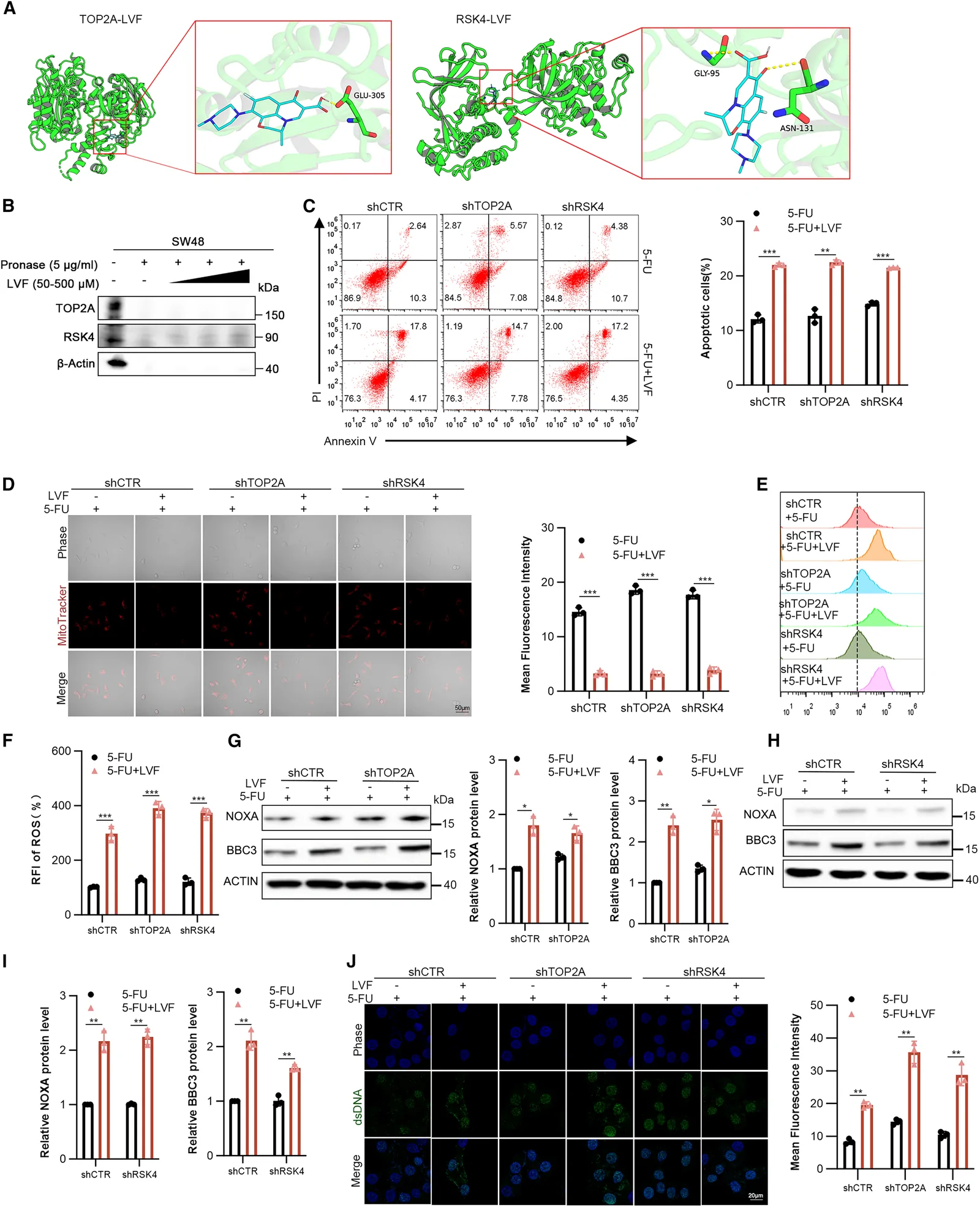
Role of TOP2A and RSK4 in CRC cells treated with LVF combined with 5-FU (A) Molecular docking of LVF with TOP2A and LVF with RSK4. (B) DARTS-WB detection of LVF binding to TOP2A and RSK4. (C) Apoptosis analysis by annexin V/PI staining in control short hairpin RNA (shCTR), shTOP2A, and shRSK4 SW48 cells after indicated treatments. (D) Mitochondrial morphology and mass visualized by MitoTracker staining in shCTR, shTOP2A, and shRSK4 SW48 cells following treatment with LVF + 5-FU. Scale bars, 50 μm. (E and F) Total intracellular ROS levels detected by DCFH-DA flow cytometry in shCTR, shTOP2A, and shRSK4 SW48 cells after LVF + 5-FU treatment. (G) Western blot analysis of NOXA and BBC3 protein expression in shTOP2A SW48 cells treated with LVF + 5-FU. (H and I) Western blot analysis of NOXA and BBC3 protein expression in shRSK4 SW48 cells treated with LVF + 5-FU. (J) Immunofluorescence analysis of cytoplasmic dsDNA in shCTR, shTOP2A, and shRSK4 SW48 cells after LVF + 5-FU treatment. Scale bars, 20 μm. Data are presented as means ± SD from three independent experiments with three technical replicates each. Statistical comparisons were performed using unpaired Student’s *t* test or one-way ANOVA. ∗*p* < 0.05, ∗∗*p* < 0.01, ∗∗∗*p* < 0.001.

To test this, we first performed molecular docking analysis, which predicted binding of LVF to both TOP2A and RSK4 (Figure 7A). To experimentally validate these predictions, we performed drug affinity responsive target stability combined with western blotting (DARTS-WB) assays. Notably, no binding of LVF to TOP2A was detected, whereas a weak but detectable binding interaction was observed between LVF and RSK4 (Figure 7B). We then knocked down *TOP2A* or *RSK4* individually in SW48 cells and assessed the functional requirement of each protein (Figure S21A). Strikingly, loss of either protein resulted in comparable preservation of chemosensitization, with sensitization persisting unchanged and all examined phenotypes remaining intact, including cytotoxicity (Figure S21B) and apoptotic induction (Figure 7C), mitochondrial morphological changes (Figure 7D), intracellular ROS generation and mitochondrial membrane depolarization (Figures 7E and 7F; Figure S21C), NOXA/BBC3 upregulation (Figures 7G–7I), and cytoplasmic dsDNA release (Figure 7J). These findings demonstrate that neither TOP2A nor RSK4 is required for LVF-mediated 5-FU chemosensitization, prompting us to investigate alternative mechanisms. Collectively, these data establish that LVF-mediated 5-FU chemosensitization operates through the DDR-TP53-mitochondrial axis independently of the canonical antibiotic target TOP2A and the reported effector RSK4, thereby establishing a non-canonical mechanism distinct from the antibacterial activity of LVF.

## Discussion

LVF primes *TP53*-WT CRC cells for 5-FU cytotoxicity through a DDR-OXPHOS-dependent mechanism. Rather than inducing extensive DNA damage, LVF triggers limited genotoxic stress that activates cellular DNA repair responses, manifesting as discrete γH2AX foci without widespread chromatin fragmentation or cell death. Mechanistically, this repair response upregulates OXPHOS activity, consistent with the ATP-intensive nature of base-excision repair.^26^^,^^27^^,^^28^ This OXPHOS upregulation increases electron flux through the respiratory chain, promoting electron leakage and mitochondrial ROS accumulation. Consequently, this mitochondrial stress, in conjunction with DDR signaling, activates *TP53*-dependent *NOXA*/*BBC3* transcription, priming cells for mitochondrial apoptosis and thereby enhancing 5-FU cytotoxicity.^29^ When we knocked out *TP53* or deleted both *NOXA*/*BBC3*, the chemosensitization effect of LVF was abolished. Similarly, scavenging mitochondrial ROS or blocked OXPHOS eliminated this phenotype, providing strong evidence that DDR-*TP53*-induced mitochondrial signals are both necessary and sufficient for LVF-mediated 5-FU chemosensitisation.

The *TP53*-*NOXA*/*BBC3* axis serves as a critical regulator of mitochondrial apoptosis in CRC.^30^^,^^31^ NOXA selectively neutralizes MCL-1, the primary anti-apoptotic guardian of mitochondrial integrity that is aberrantly upregulated in multiple malignancies including CRC and is a major driver of 5-FU chemoresistance, whereas BBC3 directly activates BAX/BAK, together ensuring MOMP, a point of no return in chemosensitivity.^32^^,^^33^ Our data mechanistically connect LVF-induced DNA damage to TP53 activation and subsequent upregulation of *NOXA* and *BBC3*, bypassing traditional cytotoxic synergy. This explains why *TP53* KO completely abrogates LVF-mediated 5-FU chemosensitization, confirming TP53 as an essential mediator rather than a passive indicator.

Unlike other fluoroquinolones, whose anticancer activity is linked to TOP2A-mediated DNA breaks,^34^^,^^35^ LVF induces nuclear DNA damage as the primary source of cytoplasmic dsDNA distinct from mtDNA release mechanisms, as evidenced by marked γH2AX foci formation. However, the DNA-damaging effect of LVF is modest and selective, triggering OXPHOS activation without overwhelming genomic integrity. We observed a transient ATP rise that reflects this compensatory increase in OXPHOS activity, consistent with a stress-induced hypermetabolism response to meet increased ATP demand during DNA repair.^36^^,^^37^ This metabolic adaptation thereby primes cells for apoptosis upon 5-FU exposure, consistent with our observation that inhibiting complex III with AA blocks this chemosensitization. Future studies should dissect whether LVF-induced dsDNA engages alternative sensors such as interferon gamma-inducible protein 16 (IFI16) or metabolic effectors such as S-adenosylhomocysteine/S-adenosylmethionine (SAH/SAM) ratios to activate TP53.

### Limitations of this study

First, although validated in Apc^min/+^ and xenograft models, studies in *TP53*^*fl/fl*^; *Villin-Cre* mice are needed to establish a causal link between intestinal epithelial *TP53* deletion to LVF resistance; we are currently generating these models. Second, RNA-seq data were derived from different sources (SW48 for LVF and GEO-sourced HCT116 for 5-FU). To eliminate bias, parallel RNA-seq analysis of matched patient-derived organoids (PDOs) treated with LVF + 5-FU will provide a more comprehensive profile that captures tumor heterogeneity.^38^ Third, while 10 mg/kg LVF showed no gross toxicity, systematic histopathology of liver/heart and cardiac troponin monitoring are mandated, especially given the QT prolongation risk associated with fluoroquinolones.^34^^,^^39^ Phase 0 microdosing trials in CRC patients could define the human equivalent dose. Fourth, all animal experiments were conducted exclusively in male mice to minimize hormonal variability as a confounding factor; future studies must include both male and female subjects to confirm the generalizability of our findings Fifth, LVF’s gyrase-independent effects suggest novel protein targets. We have verified that LVF does not directly inhibit TOP2A or RSK4 in CRC cells at chemosensitizing concentrations (data not shown), indicating that its priming effect proceeds via previously uncharacterized binding partners. We are currently conducting affinity chromatography and thermal proteome profiling to identify direct binders. Preliminary screens have identified candidate interactors potentially involved in LVF-induced mitochondrial dysfunction and oxidative stress. Characterization of these targets is ongoing.

## Resource availability

### Lead contact

Further information and requests for resources and reagents should be directed to and will be fulfilled by the lead contact, Yongli Fan (fanyongli1987@163.com).

### Materials availability

This study did not generate new, unique reagents.

### Data and code availability

This paper does not report original code. The RNA-seq data generated in this study have been deposited in NCBI GEO and are publicly available as of the date of publication. Accession number: GEO: GSE341447. This paper analyzes existing, publicly available RNA-seq data (GEO: GSE154146). These datasets are available from NCBI GEO. Any additional information required to reanalyze the data reported in this paper is available from the lead contact upon request.

## Acknowledgments

This work was financially supported by the Medical Technology Co-construction Project Fund of Henan (LHGJ20230419), the Medical Education Research Project of Henan Province (Wjlx2022214), the 10.13039/501100006407Natural Science Foundation of Henan Province (252300420584), the Science and Technology Research Project of Henan Province (262102310445), and the Open Project Research of the First Affiliated Hospital of Henan University (KFQN24006).

## Author contributions

C.H., investigation, writing – original draft, formal analysis; C.S., investigation, formal analysis, visualization; H.W., investigation, formal analysis; Y.W., formal analysis; Y.Z., formal analysis; W.L., formal analysis; A.Y., formal analysis; Y.Y., formal analysis; J.W., writing – review & editing; J.G., writing – review & editing; Z.H., conceptualization, writing – review & editing. J.C., conceptualization, investigation, writing – original draft, writing – review & editing, visualization; Y.F., conceptualization, investigation, writing – review & editing, visualization.

## Declaration of interests

The authors declare no competing interests.

## STAR★Methods

### Key resources table

**Table:** 

REAGENT or RESOURCE	SOURCE	IDENTIFIER
**Antibodies**		

Anti-Cleaved Caspase 3	Proteintech	Cat# 25128-1-AP; RRID: AB_3073913
Anti-BAX	ABclonal	Cat# A20227; RRID: AB_3663017
Anti-BCL2	ABclonal	Cat# A19693; RRID: AB_2862738
Anti-Cleaved PARP	Proteintech	Cat# 66520-1-Ig; RRID: AB_2881883
Anti-NOXA	Proteintech	Cat# 17418-1-AP; RRID: AB_3669271
Anti-p53	ABclonal	Cat# A0263; RRID: AB_2757076
Anti-BBC3	ABclonal	Cat# A3752; RRID: AB_2863135
Anti-Phospho-Histone H2AX-S139	ABclonal	Cat# AP1555; RRID: AB_3698593
Anti-Cytochrome C	Abcam	Cat# ab133504; RRID: AB_2802115
Anti-VDAC1	ABclonal	Cat# A19707; RRID: AB_2862746
Anti-TOP2A	ABclonal	Cat# A4389; RRID: AB_2863260
Anti-β-actin	ABclonal	Cat# AC026; RRID: AB_2768234
HRP-conjugated goat anti-rabbit IgG secondary antibody	ABclonal	Cat# AS014; RRID: AB_2769854
HRP-conjugated goat anti-mouse IgG secondary antibody	ABclonal	Cat# AS003; RRID: AB_2769851

**Chemicals, peptides, and recombinant proteins**		

Levofloxacin (LVF)	Aladdin Biochemical	Cat# 100986-85-4
5-Fluorouracil (5-FU)	MedChemExpress	Cat# HY-90006
KU-55933	MedChemExpress	Cat# HY-12016
Rotenone	MedChemExpress	Cat# HY-B1756
Dimethyl malonate	MedChemExpress	Cat# HY-Y1787
Antimycin A	Merck (Sigma-Aldrich)	Cat# A8674
Mitoquinone mesylate	MedChemExpress	Cat# HY-100116A
Sodium azide (NaN_3_)	Merck (Sigma-Aldrich)	Cat# S2002
MitoTracker Red CMXRos (fluorescent probe)	Beyotime	Cat# C1049B
JC-1 (fluorescent probe)	Beyotime	Cat# C2006
MitoSOX™ Red (mitochondrial superoxide indicator)	Invitrogen/Thermo Fisher	Cat# M36008
DCFH-DA (cellular ROS indicator)	MedChemExpress	Cat# HY-D0940
TRIzol (for qPCR RNA extraction)	Solarbio	Cat# R1100
DMEM (cell culture medium)	Gibco	Cat# 11965092
Fetal bovine serum (FBS)	PAN-Biotech	Cat# ST30-3302
M-MLV Reverse Transcriptase	Vazyme	Cat# R021-01
SYBR Green qPCR Master Mix	Monad Biotech	Cat# MQ10701S
Neofect™ transfection reagent	NEOFECT	Cat# TF201201
Proteinase K	Merck (Sigma Aldrich)	Cat# HY-108717
CCK-8 Cell Counting Kit	Solarbio	Cat# CA1210
TUNEL Apoptosis Detection Kit (FITC)	Servicebio	Cat# G1501-50
ATP Assay Kit (luminometric)	Beyotime	Cat# S0026
Lactate Assay Kit (colorimetric)	Beyotime	Cat# S0227S
Annexin V-FITC/PI Apoptosis Detection Kit	Beyotime	Cat# C1062L
BCA Protein Assay Kit	Solarbio	Cat# PC0020
Mycoplasma Detection Kit (PCR)	MedChemExpress	Cat# HY-K0552

**Deposited data**		

RNA-seq data	This paper	GEO: GSE341447
RNA-seq data	NCBI GEO	GEO: GSE154146

**Experimental models: Cell lines**		

HEK293T	ATCC	CRL-3216
SW48	ATCC	CCL-231
HCT116	ATCC	CCL-247
HT29	ATCC	HTB-38
SW480	ATCC	CCL-228

**Experimental models: Organisms/strains**		

BALB/c-nude mice (male, 5-week-old)	Shouzheng Pharma (Wuhan)	N/A
Apc^min/+^ mice (male, 3-month-old)	Shouzheng Pharma (Wuhan)	N/A

**Oligonucleotides**		

sgRNA NOXA: ACGCTCAACCGAGCCCCGCG	This study	N/A
sgRNA BBC3: AGATTGTACAGGACCCTCCAGGG	This study	N/A
sgRNA TP53: AGACCTAAGAGCAATCAGTG	This study	N/A
shTOP2A: GCTGATGATGAGTACGAGA	This study	N/A
shRSK4: GGAAGTATCAGCGTACGAA	This study	N/A
qPCR primers (see Table S1)	This study	N/A

**Recombinant DNA**		

Lenti-CRISPR-v2 (for sgRNA cloning)	Addgene	Cat# 52961
pLKO.1 (for shRNA cloning)	Addgene	Cat# 10878

**Software and algorithms**		

DESeq2	R/Bioconductor	1.38.0
PyMOL	Schrödinger	3.1.0
AutoDock Tools	Scripps Research	1.5.7
AutoDock Vina	Scripps Research	1.1.2
GraphPad Prism	GraphPad Software	8.5.0

### Experimental model and study participant details

#### Cell lines

HEK293T, SW48, HCT116, HT29 and SW480 cell lines were purchased from the American Type Culture Collection (ATCC, USA). SW48 is a colorectal carcinoma cell line isolated from a 82-year-old female patient and harbors wild-type TP53. HCT116 is a colorectal carcinoma cell line isolated from an adult male patient and harbors wild-type TP53. HT29 is a colorectal adenocarcinoma cell line isolated from a 44-year-old female patient and carries TP53-mutant alleles. SW480 is a colorectal adenocarcinoma cell line isolated from the primary tumor of a 50-year-old male Dukes’ type B patient and carries TP53-mutant alleles. HEK293T is a human embryonic kidney cell line used for lentivirus production. All cell lines were maintained in DMEM supplemented with 10% fetal bovine serum and 1% penicillin-streptomycin at 37 °C in a humidified atmosphere containing 5% CO_2_. All cell lines were authenticated by short tandem repeat (STR) profiling within the last 6 months, and the STR profiling results were consistent with the standard cell line profiles provided by ATCC. All cell lines used in this study were routinely tested for mycoplasma contamination using a mycoplasma PCR detection kit (MedChemExpress Cat# HY-K0552), and no mycoplasma contamination was detected during the entire experimental period.

Sex-based study limitation statement: All cell lines utilized in the present study were derived from male and female human patients as specified above. In this *in vitro* cell experiment system, cell proliferation, apoptosis and molecular pathway activation induced by levofloxacin combined with 5-fluorouracil were not observed to be associated with the sex origin of the cell lines. Given the focused *in vitro* experimental design, systematic evaluation of sex-dependent differences in drug efficacy was not performed, which serves as a minor limitation of this cell-based study.

#### Animals

Male BALB/c-nude mice (5 weeks old) and male Apc^min/+^ mice (3 months old) were procured from Shouzheng Pharma (Wuhan) Biotechnology Co., Ltd. (Wuhan, China).

A total of 4 mice per group were used, and animals were randomly assigned to experimental groups. BALB/c-nude mice are immunodeficient mice with severe T-cell deficiency due to thymic aplasia, commonly used for xenograft tumor studies, enabling efficient engraftment of human cancer cells. The Apc^min/+^ mouse is a well-established transgenic model of familial adenomatous polyposis that carries a germline nonsense mutation at codon 850 of the murine adenomatous polyposis coli (Apc) gene, resulting in spontaneous formation of multiple intestinal adenomas and serving as a widely used preclinical model for colorectal cancer.

All animals were housed in grouped cages under specific-pathogen-free conditions with a 12-hour light/dark cycle at 22 ± 2 °C and provided with standard chow diet and water *ad libitum*.

All animal experiments were conducted in accordance with the Guidelines for the Care and Use of Laboratory Animals and were approved by the Biomedical Research Ethics Committee of Henan University (#HUSOM2024-648). No blinding was used in this study. Only male mice were included in animal experiments to eliminate the confounding effects of female hormonal fluctuations on tumor growth and drug response. Accordingly, the potential influence of sex differences on *in vivo* therapeutic efficacy was not explored in the current animal study.

### Method details

#### Cell culture and treatment

SW48 (TP53-WT), HCT116 (TP53-WT), HT29 (TP53-mutant) and SW480 (TP53-mutant) cells were cultured in DMEM (Gibco, 11965092) supplemented with 10% fetal bovine serum (PAN-Biotech, ST30-3302) at 37 °C in a humidified atmosphere containing 5% CO_2_. HEK293T cells were cultured in the same medium for lentivirus production. For drug treatments, cells were pretreated with LVF (0, 25, 50, 100, or 200 μM) for 48 hours, followed by co-treatment with 5-FU (0, 10, 20, or 50 μM) and LVF for an additional 48 hours.

#### RNA sequencing (RNA-seq)

SW48 cells treated with LVF (100 μM) for 5 days were subjected to RNA-seq. Total RNA was extracted using TRIzol (R1100, Solarbio) and assessed for quality using a NanoDrop spectrophotometer and Agilent 2100 Bioanalyzer. Libraries were prepared using the NEBNext® Ultra™ RNA Library Prep Kit (NEB) and sequenced on an Illumina NovaSeq 6000 platform (150 bp paired-end reads). Reads were aligned to the GRCh38/hg38 genome. Differentially expressed genes were identified using DESeq2 v1.38.0 (R package).

#### Molecular docking analysis

The Structured Data File (SDF) of levofloxacin (LVF) was retrieved from the PubChem database (PubChem: 149096). Protein structures of TOP2A (PDB: 1ZXM) and RSK4 (PDB: 6G78) were obtained from the Protein Data Bank and optimized using PyMOL-3.1.0. Water molecules and small-molecule ligands were removed, followed by hydrogenation and charge assignment using AutoDockTools-1.5.7. These structures were saved in pdbqt format. Molecular docking was performed using AutoDock Vina v1.1.2. The key targets served as receptors and LVF as the ligand. The docking grid was centered on the ATP-binding pocket of TOP2A and the active site of RSK4 with grid box dimensions set to 20 × 20 × 20 Å and spacing of 0.375 Å to ensure accurate sampling of the known binding pockets. Binding energies were calculated and output results were generated. Finally, PyMOL was used to visualize the results. The affinity value (kcal/mol) represents the binding strength between ligand and receptor, with lower values indicating stronger and more stable binding. PyMOL was also used for visual analysis of interaction patterns.

#### CRISPR-mediated knockout and shRNA-Mediated knockdown

For CRISPR knockout, sgRNAs targeting NOXA, BBC3, or TP53 were cloned into lenti-CRISPR-v2 (Addgene, Cat#52961). Lentivirus was produced in 293T cells using Neofect™ reagent (Cat#TF201201). SW48 cells were infected and selected with puromycin for 1-2 weeks. Monoclonal populations were established and validated by sequencing and western blotting. sgRNA sequences: NOXA, ACGCTCAACCGAGCCCCGCG; BBC3, AGATTGTACAGGACCCTCCAGGG; TP53, AGACCTAAGAGCAATCAGTG.

For knockdown, shRNAs targeting TOP2A or RSK4 were cloned into pLKO.1 (Addgene, Cat#10878). SW48 cells were infected and selected with puromycin for 5-7 days. Efficiency was validated by qRT-PCR and western blotting. shRNA sequences: shTOP2A, GCTGATGATGAGTACGAGA; shRSK4, GGAAGTATCAGCGTACGAA.

#### Animals

For the subcutaneous xenograft model, SW48 cells (MSS-type, TP53-WT; 1 × 10^6^) were injected subcutaneously into the right flank of 5-week-old male BALB/c-nude mice. When tumors reached approximately 100 mm^3^, mice were randomly assigned to four treatment groups (*n* = 4 per group) and received intraperitoneal injections of vehicle, LVF (10 mg/kg), 5-FU (25 mg/kg), or their combination (dissolved in PBS, injection volume 10 mL/kg) every 2 days over a 3-week period. Tumor volumes were calculated using the formula (W^2^ × L)/2, where W represents the shorter axis and L the longer axis. At the endpoint, mice were euthanized by CO_2_ asphyxiation followed by cervical dislocation, and tumors were surgically excised for subsequent analyses.

For the primary colorectal cancer model, 3-month-old male Apc^min/+^ mice were randomly divided into four groups (*n* = 4 per group): control, LVF, 5-FU, and combination (LVF + 5-FU). These mice received intraperitoneal injections (dissolved in PBS, injection volume 10 mL/kg) every 2 days. After a two-month treatment regimen, mice were euthanized as described above, and the number of intestinal polyps was meticulously counted.

#### Cell viability assay

Cells were pretreated with LVF (100 μM) for 48 hours in culture flasks containing medium with 10% serum, harvested, and seeded in 96-well plates (2000 cells/well). After attachment, cells were cotreated with 5-FU (0, 10, 20, or 50 μM) and LVF (100 μM) for 48 hours at 37 °C in 5% CO_2_ in medium with 1% serum. CCK-8 solution (10 μL; CA1210, Solarbio) was then added, and plates were incubated for an additional 1.5 hours. Optical density was measured at 450 nm using a microplate reader (BioTek Epoch), and cell survival rates were calculated according to the manufacturer’s instructions.

#### Western blotting

Cells were pretreated with 100 μM LVF for 48 hours, followed by co-treatment with 100 μM LVF and 50 μM 5-FU for an additional 48 hours. For time-course experiments, cells were collected at the indicated time points (days 0-7). Proteins were separated by 10%-15% SDS-PAGE and transferred to PVDF membranes. Membranes were blocked with 5% skim milk in TBST for 1 hour at room temperature, followed by incubation with primary antibodies at 4 °C overnight. After washing, membranes were incubated with HRP-conjugated secondary antibodies for 1 hour at room temperature. Protein bands were visualized using an imaging system (Baygene). The following primary and secondary antibodies were used (dilutions in 5% BSA-TBST): Anti-Cleaved Caspase-3 (rabbit polyclonal, Proteintech, Cat#25128-1-AP, RRID: AB_3073913) at 1:1000; Anti-BAX (rabbit monoclonal, ABclonal, Cat#A20227, RRID: AB_3663017) at 1:2000; Anti-BCL2 (rabbit monoclonal, ABclonal, Cat#A19693, RRID: AB_2862738) at 1:2000; Anti-Cleaved PARP (rabbit polyclonal, Proteintech, Cat#66520-1-Ig, RRID: AB_2881883) at 1:1000; Anti-NOXA (rabbit polyclonal, Proteintech, Cat#17418-1-AP, RRID:AB_3669271) at 1:1000; Anti-p53 (rabbit monoclonal, ABclonal, Cat#A0263, RRID: AB_2757076) at 1:2000; Anti-BBC3 (rabbit monoclonal, ABclonal, Cat#A3752, RRID: AB_2863135) at 1:2000; Anti-Phospho-Histone-H2AX-S139 (rabbit monoclonal, ABclonal, Cat#AP1555, RRID: AB_3698593) at 1:2000; Anti-Cytochrome C (rabbit monoclonal, Abcam, Cat#ab133504, RRID: AB_2802115) at 1:1000; Anti-VDAC1 (rabbit polyclonal, ABclonal, Cat#A19707, RRID: AB_2862746) at 1:2000; Anti-TOP2A (rabbit polyclonal, ABclonal, Cat#A4389, RRID: AB_2863260) at 1:1000; Anti-β-actin (mouse monoclonal, ABclonal, Cat#AC026, RRID: AB_2768234) at 1:5000; HRP-conjugated goat-anti-rabbit IgG secondary antibody (ABclonal, Cat#AS014, RRID: AB_2769854) at 1:5000; HRP-conjugated goat-anti-mouse IgG secondary antibody (ABclonal, Cat#AS003, RRID: AB_2769851) at 1:5000.

#### Drug affinity responsive target stability (DARTS) assay

Cells were harvested and lysed in M-PER lysis buffer supplemented with protease and phosphatase inhibitor cocktails for 10 minutes on ice. Cell lysates were mixed with 10× TNC buffer, followed by centrifugation at 12,000 rpm for 10 minutes at 4 °C. The supernatants were collected, aliquoted, and incubated with the indicated concentrations of LVF (0, 50, 100, and 200 μM) at 25 °C with shaking at 600 rpm for 3 hours. Subsequently, freshly diluted Pronase was added to a final concentration of 5 μg/mL, followed by incubation at 25 °C with shaking at 600 rpm for 10 minutes. The proteolysis reactions were terminated by adding 5× SDS loading buffer and heating at 100 °C for 10 minutes. The samples were then subjected to sodium dodecyl sulfate-polyacrylamide gel electrophoresis and subsequent immunoblotting analysis.

#### Real-time quantitative PCR

Cells were pretreated with 100 μM LVF for 48 hours, followed by co-treatment with 100 μM LVF and 50 μM 5-FU for an additional 48 hours. Total RNA was extracted using TRIzol (R1100, Solarbio) and reverse-transcribed into cDNA using M-MLV reverse transcriptase (R021-01, Vazyme). qRT-PCR was performed on an ABI StepOne Plus system using a SYBR Green kit (MQ10701S, Monad Biotech). Gene expression was quantified using the 2^-ΔΔCt^ method, with β-actin as the internal control. Primer sequences are listed in Table S1.

#### Cellular ROS and apoptosis analysis

Cells were harvested, washed with PBS, and subjected to the following analyses. For ROS detection, pellets were resuspended in serum-free medium containing 10 μM DCFH-DA (MedChemExpress, Cat#HY-D0940) and incubated at 37 °C for 30 minutes. DCF fluorescence was measured by flow cytometry (FCM). For apoptosis analysis, cells were double-stained with Annexin V-FITC and PI using an Annexin V-FITC/PI Apoptosis Detection Kit (Beyotime, Cat#C1062L), and apoptotic rates were determined by FCM according to the kit protocol.

#### TUNEL staining

Paraffin-embedded tumor sections (3μm) were processed using the Servicebio® FITC TUNEL Kit (G1501-50). Sections were digested with Proteinase K (20 mg/mL), rinsed, and incubated with TdT enzyme/FITC-dUTP mix (1:9) at 37 °C for 1 hour. After washing and blocking with 3% H_2_O_2_, sections were counterstained with DAPI and visualized under a fluorescence microscope. Histological services were provided by Wuhan Servicebio Technology Co., Ltd.

#### Mitochondrial staining using Mito Tracker

Cells were pretreated with LVF (100 μM) for 48 hours, seeded into confocal dishes, and then treated with LVF (100 μM) or 5-FU (50 μM) for an additional 48 hours. Subsequently, mitochondrial staining was performed using Mito Tracker Red CMXRos (Beyotime, Cat#C1049B) in serum-free medium at 37 °C for 30 minutes protected from light. Subsequently, cells were washed twice with PBS and immediately subjected to confocal microscopy (Zeiss LSM 880 or equivalent) for acquisition of fluorescence and corresponding bright-field images to assess mitochondrial distribution and morphology.

#### Mitochondrial membrane potential assay (JC-1)

Cells were pretreated with LVF (100 μM) for 48 hours, seeded into confocal dishes, and then treated with LVF (100 μM) or 5-FU (50 μM) for an additional 48 hours. The cells were then incubated with JC-1 staining solution (Beyotime, Cat#C2006) at 37 °C in the dark for 30 minutes, followed by two washes with 250 μL PBS. Fluorescence images of JC-1 aggregates (red) and monomers (green) were acquired using a confocal microscope. Mitochondrial membrane potential was evaluated based on the red/green fluorescence intensity ratio.

#### Mitochondrial ROS detection

Cells were pretreated with LVF (100 μM) for 48 hours, seeded into confocal dishes, and then treated with LVF (100 μM) or 5-FU (50 μM) for an additional 48 hours. Following incubation with MitoSOX™ Red (Invitrogen/Thermo Fisher, Cat#M36008) working solution at 37 °C for 15–20 minutes in the dark, cells were washed twice with PBS. Mitochondrial superoxide production was evaluated by capturing fluorescence and corresponding bright-field images using a confocal microscope.

#### ATP measurement

The culture medium was removed, and the cells were rinsed with PBS. An appropriate amount of lysis buffer (Beyotime, Cat#S0026) was then added, and the supernatant was collected after complete cell lysis. Subsequently, 100 μL of ATP detection reagent from the ATP Assay Kit (Beyotime, Cat#S0026) was dispensed into each well and incubated at room temperature for 5 minutes. Next, 20 μL of sample supernatant was added to the wells and mixed thoroughly. The relative light units (RLU) were finally recorded using a luminometer.

#### Transmission electron microscopy

SW48 cells were treated with LVF (100 μM) for a duration of 2 days. After treatment, the cells were rinsed and collected by centrifugation. Subsequently, they were fixed by resuspension in a solution containing 4% paraformaldehyde and 2% glutaraldehyde for at least 4 hours to ensure proper cross-linking and preservation of cellular architecture. Following fixation, the cells were embedded in Araldite 502 resin, and semi-thin sections of 80-100 nm thickness were carefully prepared using a Leica ULTRACUT R ultramicrotome. These sections were then subjected to a negative staining process with 2% uranyl acetate to enhance contrast and resolution. Cellular morphology and ultrastructure were examined and visualized using a transmission electron microscope (TEM), specifically the Hitachi HT7700, operated at an acceleration voltage of 100 keV. This sophisticated imaging was provided by Pinuofei Biological Technology Co., Ltd.

#### Hematoxylin and eosin (H&E) staining

Tissues embedded in paraffin were subjected to deparaffinization and dehydration. After rinsing with distilled water, the sections were stained with Harris’s modified hematoxylin for 3 minutes, followed by washing under running tap water for 5-10 minutes. The sections were then counterstained with eosin in an alcoholic solution for 0.5-1 minutes, dehydrated through a graded ethanol series, and cleared in xylene for 2 minutes. Microscopic images were captured for further analysis. This histological service was provided by Wuhan Servicebio Technology Co., Ltd.

#### Lactate assay

SW48 cells were treated with LVF (0, 50, 100, or 200 μM) for 48 hours. Following treatment, cell culture supernatants were harvested and centrifuged at 12,000 rpm for 5 min at 4 °C to remove cellular debris. Adherent cells were washed twice with ice-cold PBS and lysed using RIPA lysis buffer. Lactate concentrations were determined using a commercial colorimetric lactate assay kit (Beyotime, Cat#S0227S) according to the manufacturer’s instructions. Absorbance at 595 nm was measured with an Epoch microplate reader (BioTek). Intracellular lactate levels were normalized to total protein content quantified by a BCA Protein Assay Kit (Solarbio, Cat#PC0020). All experiments were performed in triplicate and repeated independently three times.

#### Colony formation assay

Cells were pretreated with LVF (100 μM) for 48 hours in culture flasks containing medium with 10% serum, harvested, and seeded into 6-well plates at a density of 1,000 cells per well. After attachment, cells were cotreated with 5-FU (10 μM) and LVF (100 μM) for 10–14 days at 37 °C in 5% CO_2_ in medium with 10% serum. The culture medium containing the indicated drugs was replenished every 3 days. At the endpoint, colonies were washed twice with PBS, fixed with 4% paraformaldehyde for 15 minutes at room temperature, and stained with 0.1% crystal violet for 20 minutes at room temperature. After washing with distilled water and air-drying, colonies containing ≥50 cells were counted manually under an inverted microscope. Cell survival rates were calculated as the percentage of colony numbers relative to the control group.

### Quantification and statistical analysis

Statistical analysis and graphical representation were performed using GraphPad Prism 8.5 (GraphPad Software, San Diego, CA, USA). All data are presented as the mean ± standard deviation (SD). Comparisons between two groups were performed using the unpaired two-tailed Student’s t-test. For multi-group comparisons, one-way ANOVA was applied, followed by appropriate post-hoc tests (e.g., Tukey’s or Dunnett’s) where applicable. A *p*-value of less than 0.05 was considered statistically significant, and significance levels are denoted as follows: ∗*p* < 0.05, ∗∗*p* < 0.01, ∗∗∗*p* < 0.001; non-significant results are marked as “ns”. All *in vitro* experiments were performed independently at least three times, with three technical replicates per experiment. All statistical details, including exact *p*-values, sample sizes (n), and the specific tests used, are provided in the corresponding figure legends or the results section.
