# Supplementary material for: Levofloxacin enhances 5-fluorouracil efficacy in colorectal cancer by activating the DDR-TP53-mitochondrial apoptosis axis
Source: iScience. 2026 Sep 17;29(10):117500. doi: 10.1016/j.isci.2026.117500 (PMC13602227; doi:10.1016/j.isci.2026.117500)

iScience, Volume *29*

Supplemental information

Levofloxacin enhances 5-fluorouracil efficacy in colorectal cancer by activating the DDR-TP53-mitochondrial apoptosis axis

Chenmeng Huang, Chenchen Sun, Han Wang, Ying Wei, Yufei Zhang, Wenfeng Li, Aping Yue, Yajing Yang, Jiajia Wang, Jindan Guo, Zhengxi Hu, Jiangyang Chi, and Yongli Fan

Supplementary material

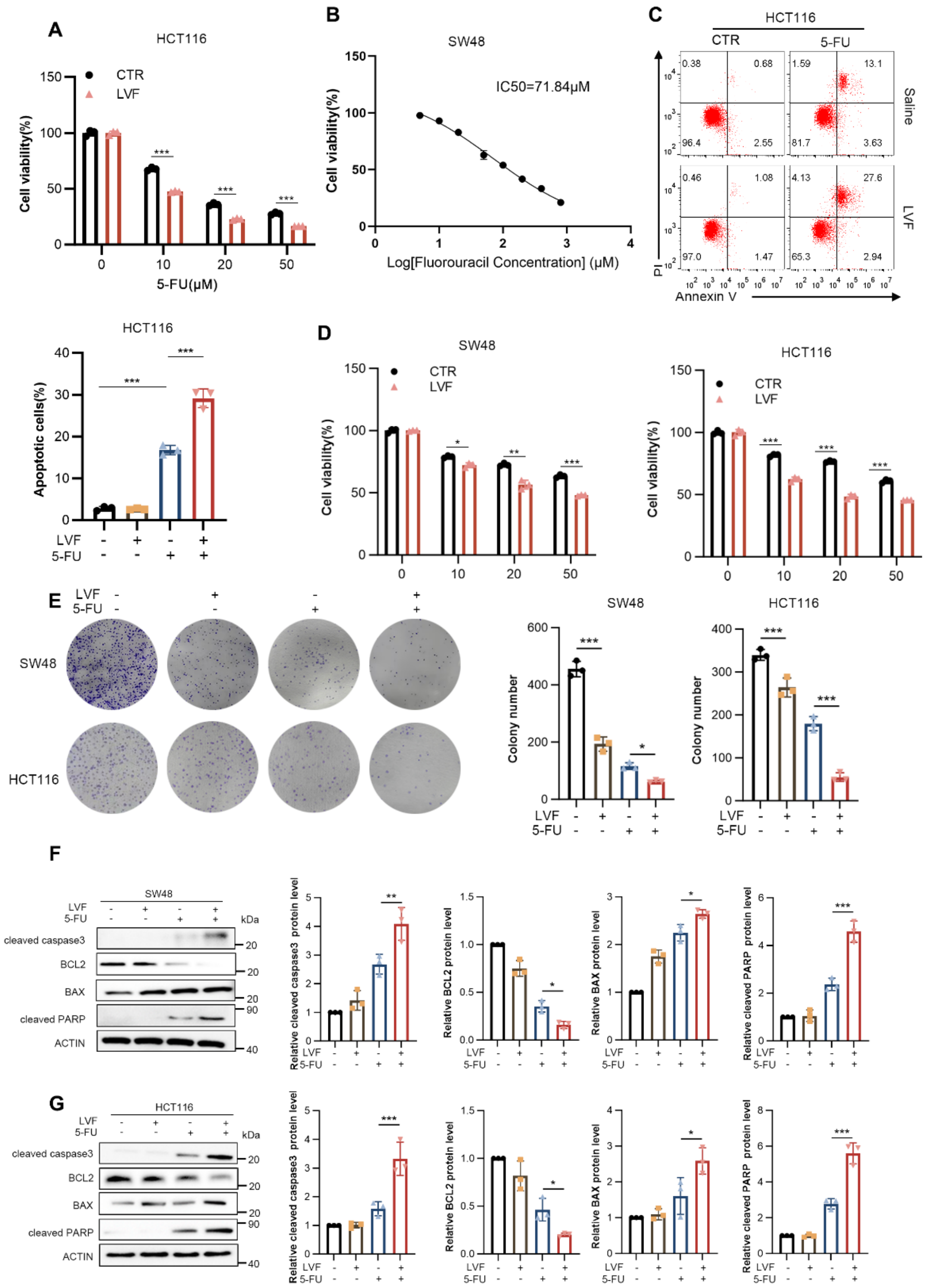

Fig. S1 Cell viability, dose-response, apoptosis, washout, and clonogenic assays in CRC cells. (**A**) Cell viability measured by CCK-8 assay in HCT116 cells treated with LVF, 5-FU, or LVF + 5-FU. (**B**) Apoptosis analysis by Annexin V-FITC/PI flow cytometry in HCT116

cells treated with LVF, 5-FU, or LVF + 5-FU. (**C**) Dose-response curve of 5-FU in SW48 cells; the 48-hour IC~50~ was determined to be 71.84 μM. (**D**) Cell viability measured by CCK-8 assay in SW48 and HCT116 cells under continuous LVF exposure versus LVF washout prior to 5-FU treatment. (**E**) Representative images and quantitative analysis of colony formation assays in SW48 and HCT116 cells treated with LVF, 5-FU, or LVF + 5-FU. (**F**) Western blot analysis of cleaved caspase-3, BCL-2, BAX, and cleaved PARP in SW48 cells treated with LVF, 5-FU, or LVF + 5-FU, with corresponding quantitative densitometry. (**G**) Western blot analysis of cleaved caspase-3, BCL-2, BAX, and cleaved PARP in HCT116 cells treated with LVF, 5-FU, or LVF + 5-FU, with corresponding quantitative densitometry. Data are presented as means ± SD from three independent experiments with three technical replicates each. Statistical comparisons were performed using unpaired Student's t-test or one-way ANOVA. *p < 0.05, **p < 0.01, ***p < 0.001.

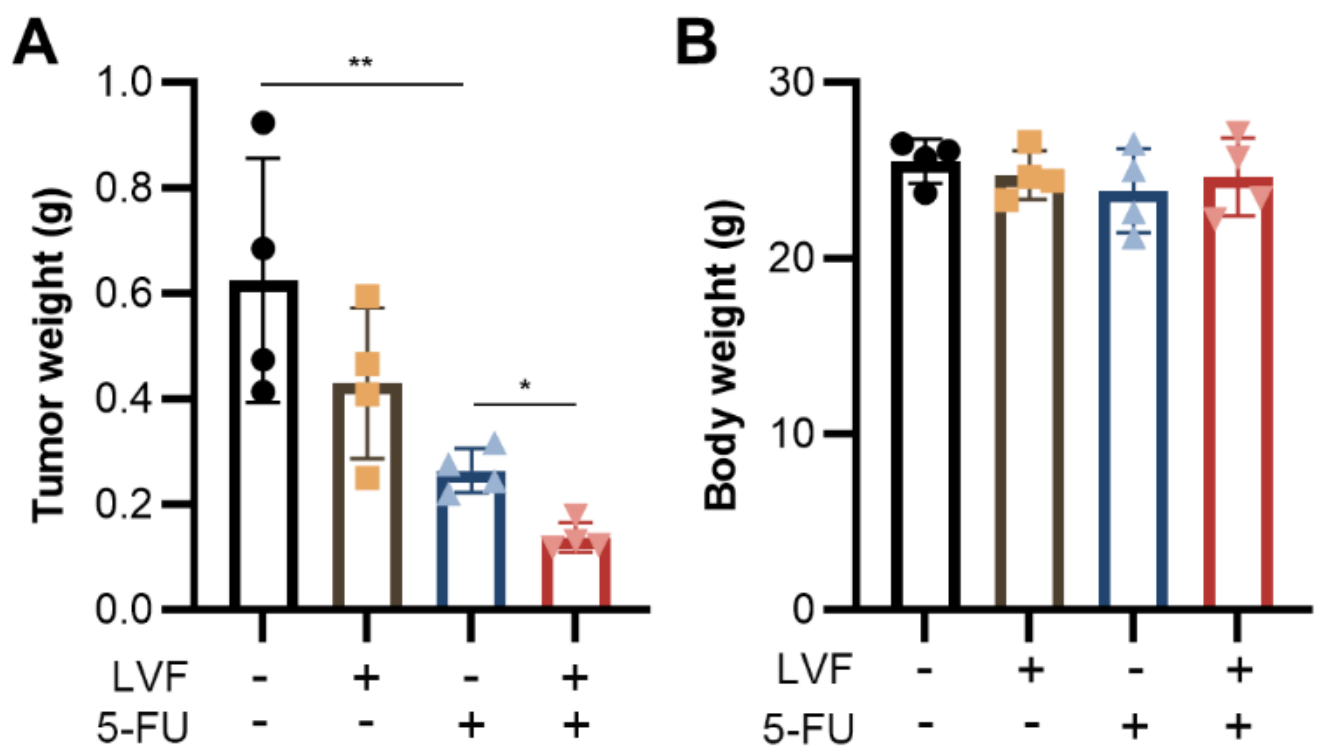

Fig. S2 Tumor weight and body weight monitoring in SW48 xenograft mice. (**A**) Tumor weight measurements at endpoint from SW48 xenograft mice treated with vehicle, LVF, 5-FU, or LVF + 5-FU. (**B**) Body weight measurements of SW48 xenograft mice monitored throughout the 3-week treatment period. Data are presented as means ± SD. Each group contained four mice. Statistical comparisons were performed using unpaired Student's t-test or one-way ANOVA. *p < 0.05, **p < 0.01, ***p < 0.001.

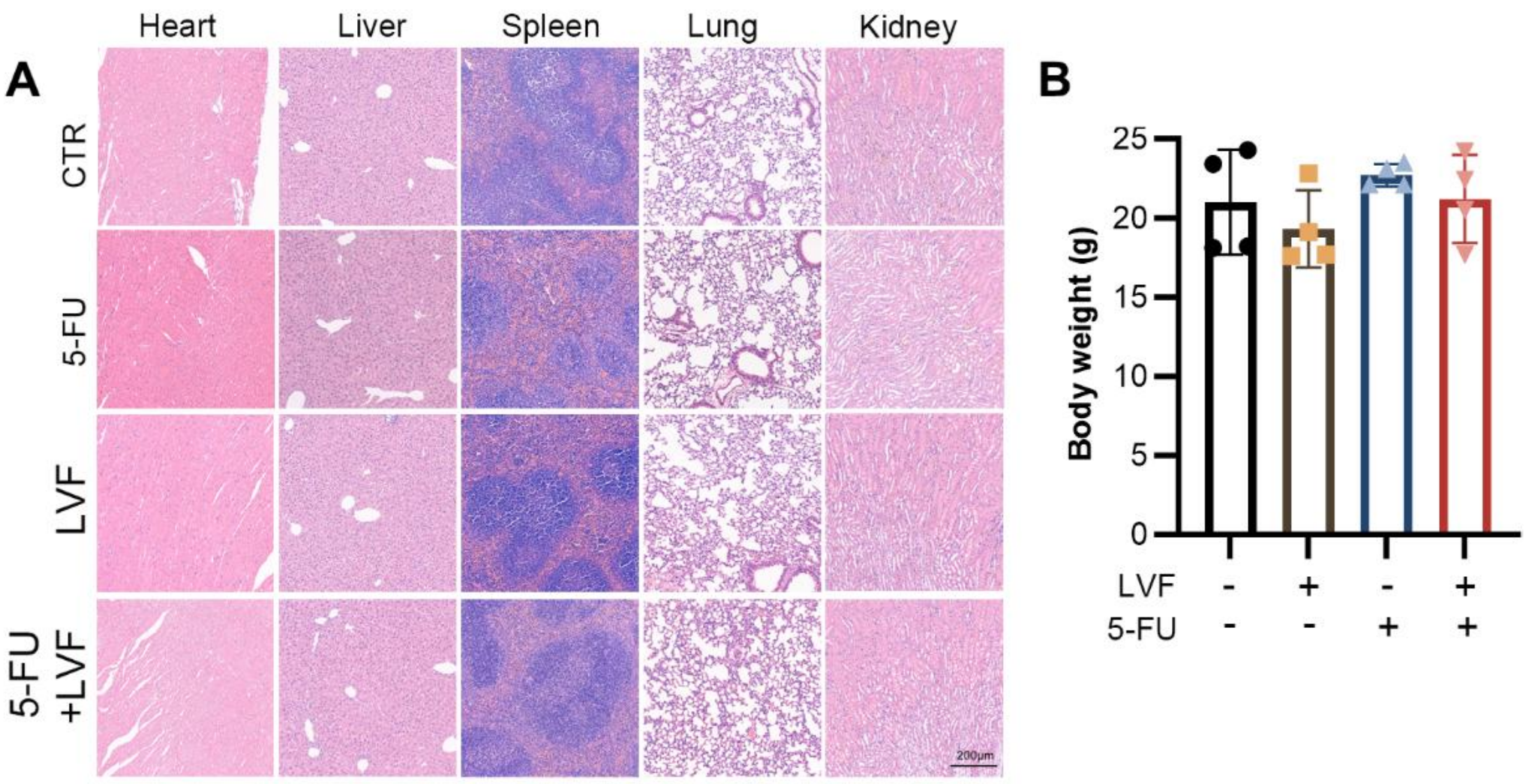

Fig. S3 Organ histology and body weight monitoring in APC$^{min/+}$ mice. (**A**) Representative hematoxylin and eosin (H&E) staining images of major organs (heart, liver, spleen, lung, and kidney) from APC$^{min/+}$ mice treated with vehicle, LVF, 5-FU, or LVF + 5-FU. Scale bar, 100 μm. (**B**) Body weight measurements of APC$^{min/+}$ mice monitored throughout the 2-month treatment period. Data are presented as means ± SD. Each group contained four mice. Statistical comparisons were performed using unpaired Student's t-test or one-way ANOVA. *p < 0.05, **p < 0.01, ***p < 0.001.

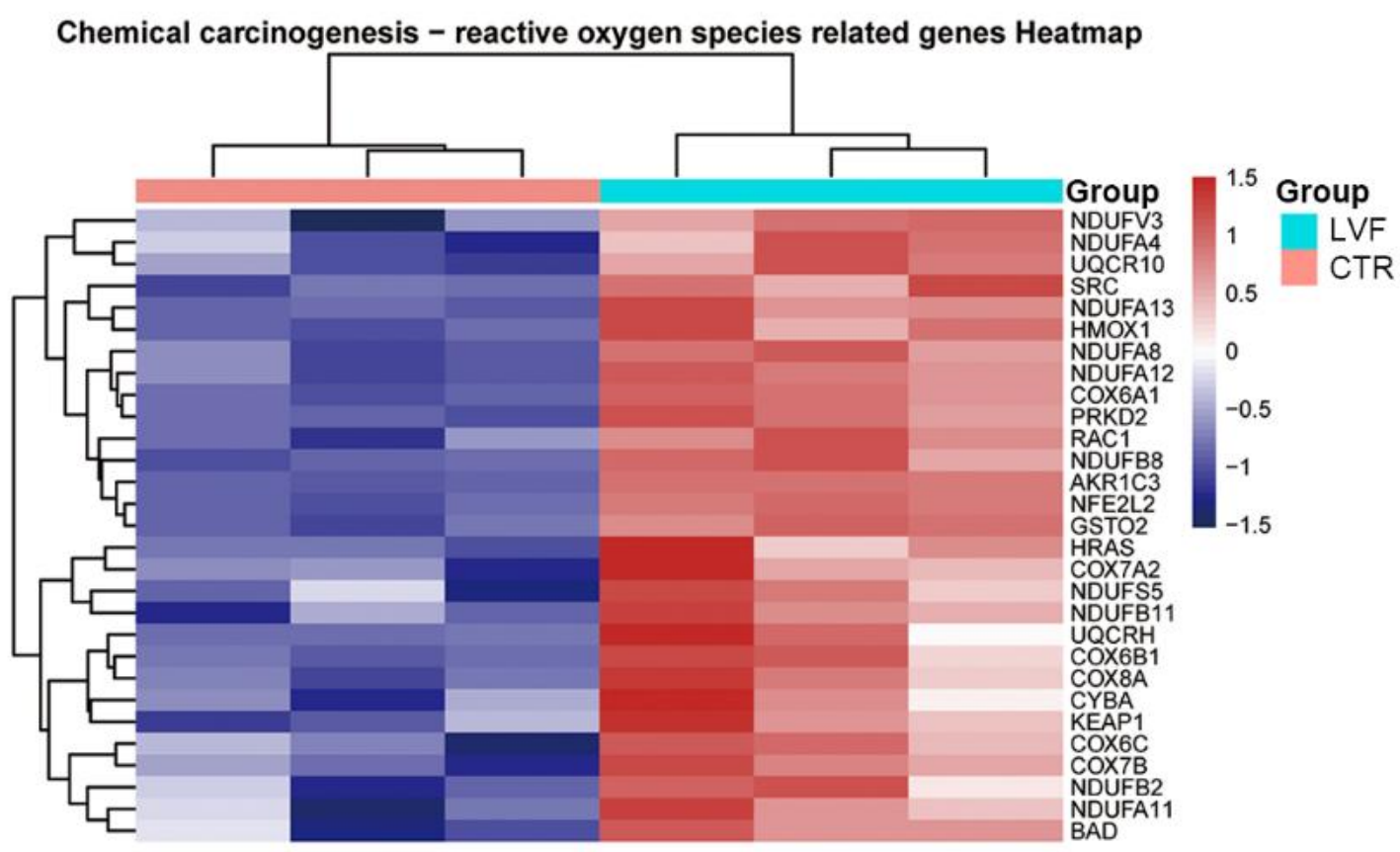

Fig. S4 Heatmap of ROS metabolism-related gene expression in SW48.

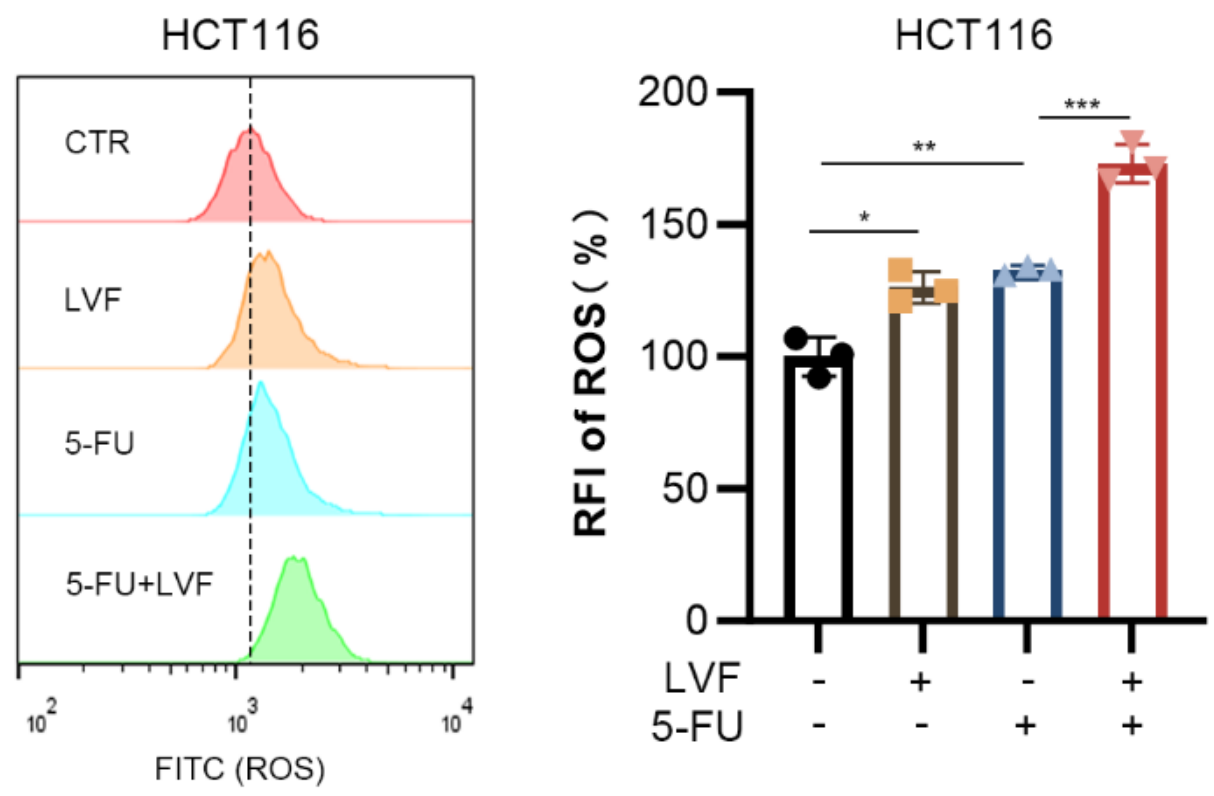

Fig. S5 Flow cytometry analysis of total intracellular ROS levels using DCFH-DA probe in HCT116 cells treated with LVF, 5-FU, or LVF + 5-FU. Data are presented as means ± SD from three independent experiments with three technical replicates each. Statistical comparisons were performed using one-way ANOVA. **p* < 0.05, ***p* < 0.01, ****p* < 0.001.

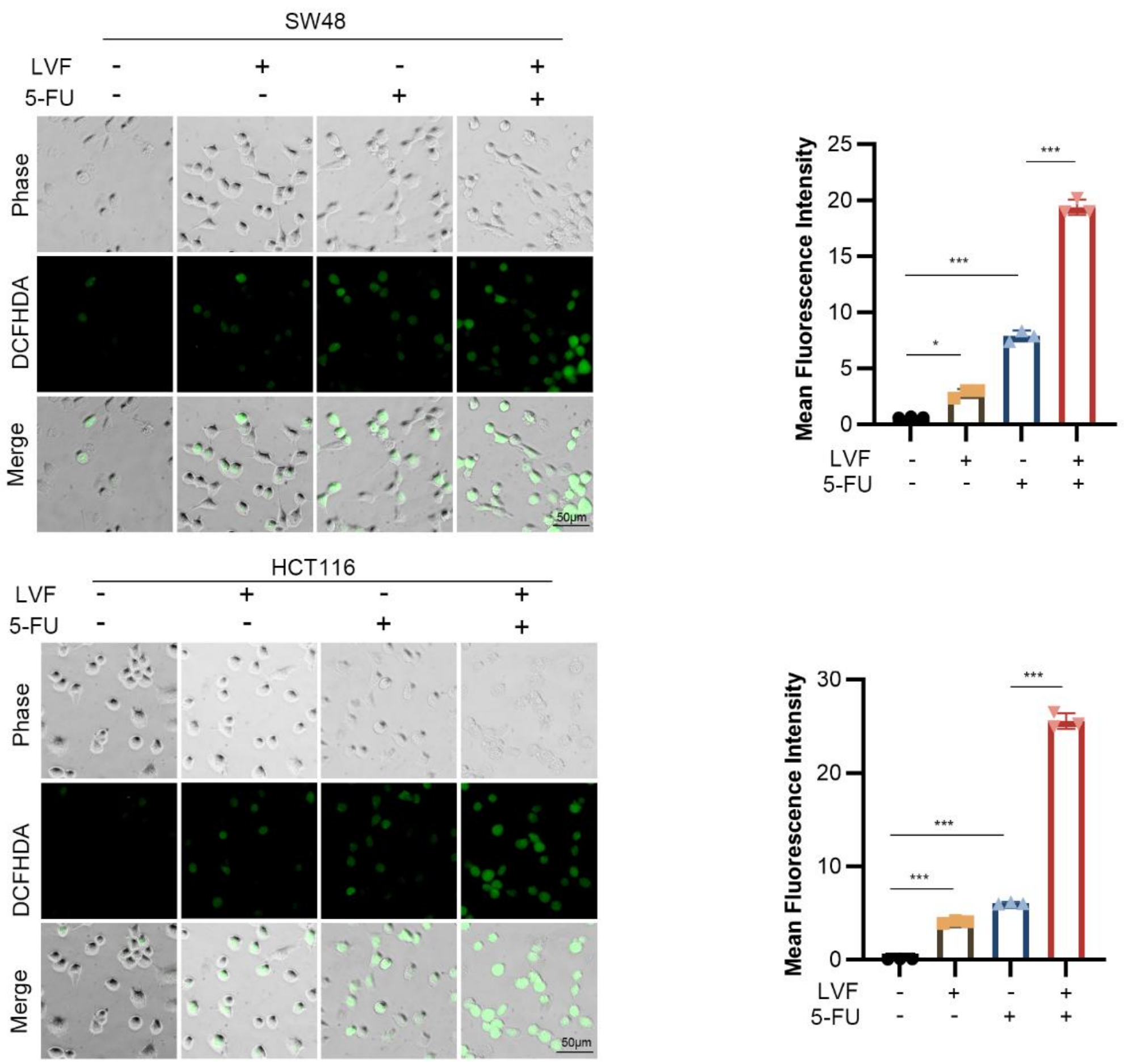

Fig. S6 Immunofluorescence staining of DCFH-DA in SW48 and HCT116 cells treated with

LVF, 5-FU, or LVF + 5-FU. Scale bar, 50 μm. Data are presented as means ± SD from three independent experiments with three technical replicates each. Statistical comparisons were performed using one-way ANOVA. **p* < 0.05, ***p* < 0.01, ****p* < 0.001.

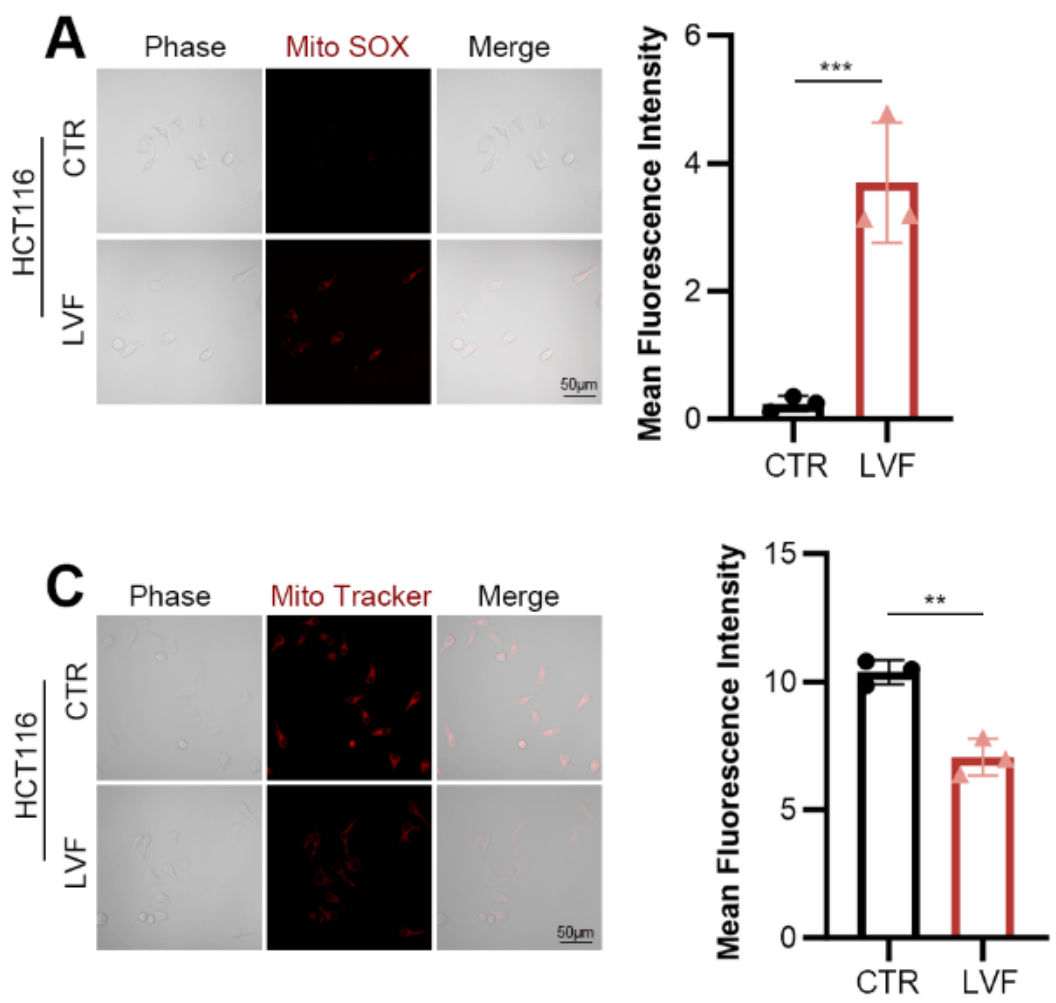

Fig. S7 Assessment of mitochondrial function and morphology in HCT116 cells. (**A**) Mitochondrial ROS levels measured by Mito SOX staining in HCT116 cells treated with LVF, 5-FU, or LVF + 5-FU. (**B**) Mitochondrial membrane potential (ΔΨm) detected by JC-1 staining in HCT116 cells treated with LVF, 5-FU, or LVF + 5-FU. (**C**) Mitochondrial morphology visualized by Mito Tracker staining in HCT116 cells treated with LVF, 5-FU, or LVF + 5-FU. Scale bar, 20 μm. Data are presented as means ± SD from three independent experiments with three technical replicates each. Statistical comparisons were performed using one-way ANOVA. *p < 0.05, **p < 0.01, ***p < 0.001.

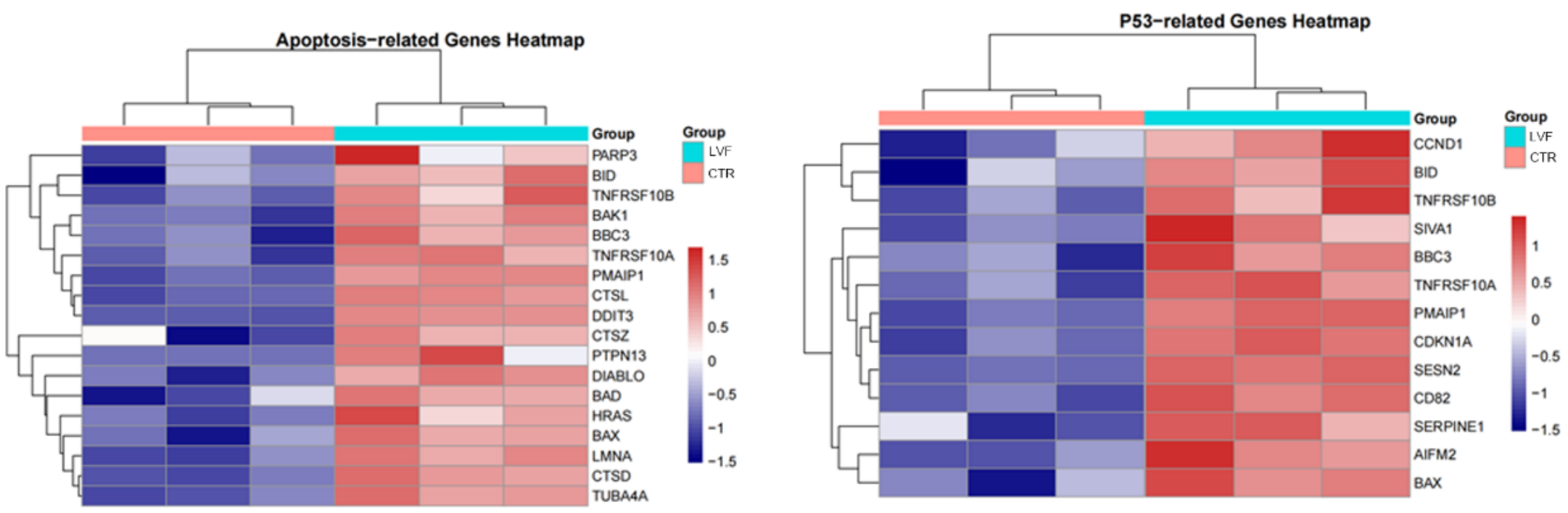

Fig. S8 Heatmap analysis of p53 and apoptosis pathway gene expression in SW48 cells.

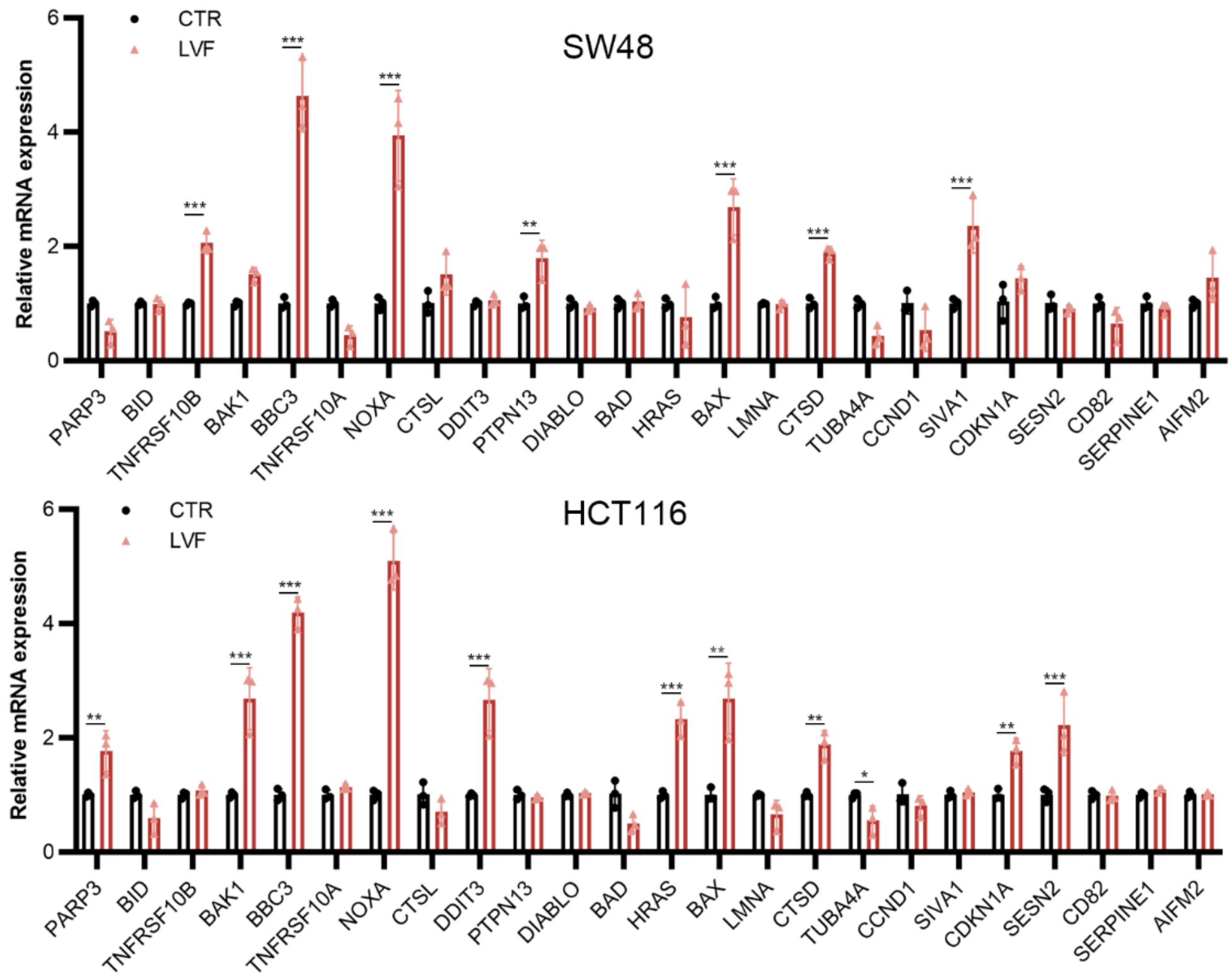

Fig. S9 qPCR analysis of TP53 and apoptosis-related gene expression in SW48 and HCT116 cells treated with LVF. Data are presented as means ± SD from three independent experiments with three technical replicates each. Statistical comparisons were performed using one-way ANOVA. *$p < 0.05$, **$p < 0.01$, ***$p < 0.001$.

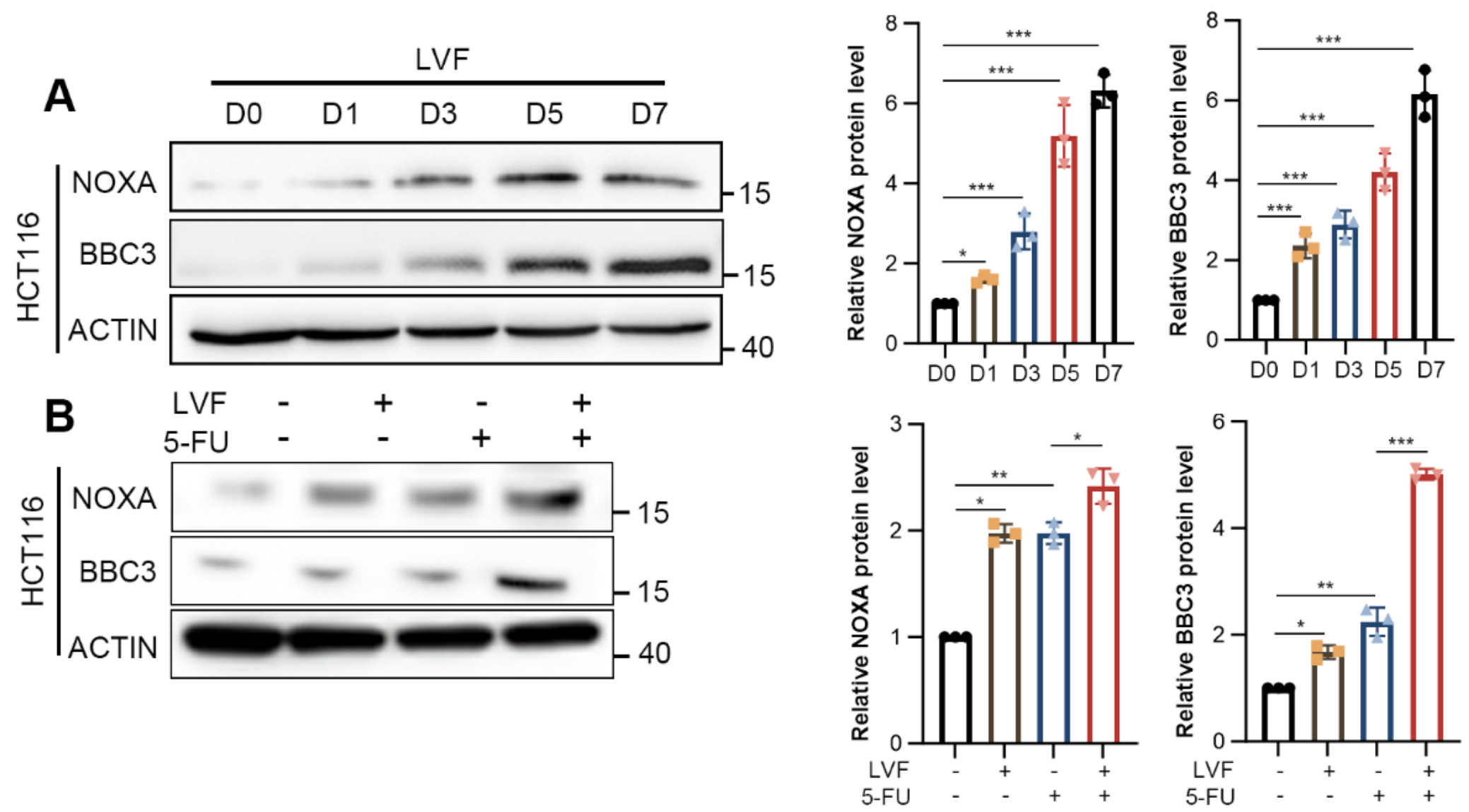

Fig. S10 Western blot analysis of NOXA and BBC3 upon LVF and LVF-5-FU combination treatment in HCT116 cells. (**A-B**) Western blot profiling of NOXA and BBC3 in HCT116

cells. (A) NOXA and BBC3 protein levels in HCT116 cells treated with LVF, 5-FU, or LVF + 5-FU for 48 hours. (B) Time-course analysis of NOXA and BBC3 protein levels in HCT116 cells treated with LVF from day 0 to day 7. Data are presented as means ± SD from three independent experiments with three technical replicates each. Statistical comparisons were performed using one-way ANOVA. *$p < 0.05$, **$p < 0.01$, ***$p < 0.001$.

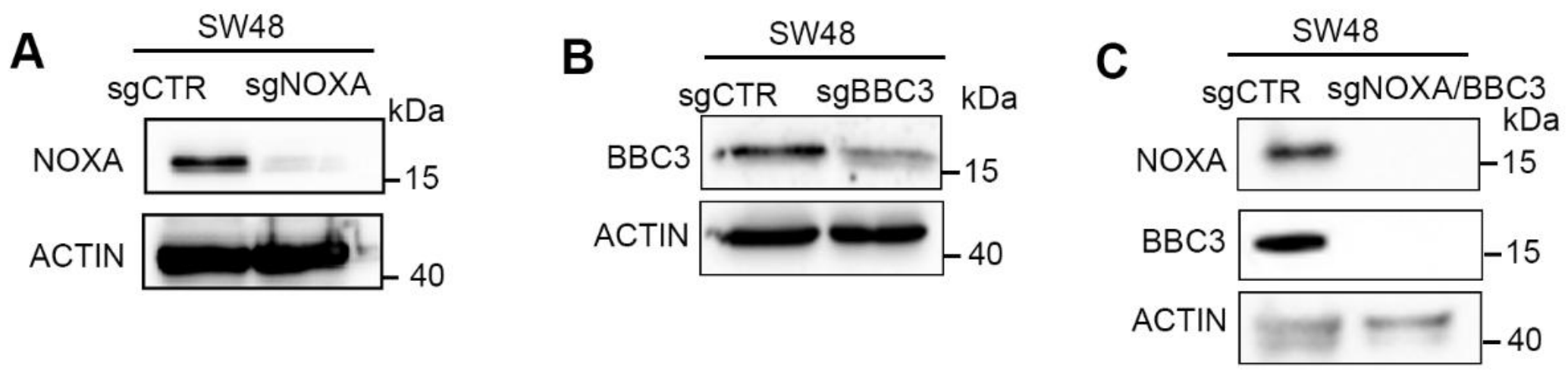

Fig. S11 Western blot analysis of NOXA and BBC3 protein levels in control, (**A**) NOXA-knockout, (**B**) BBC3-knockout, and (**C**) NOXA/BBC3-double-knockout SW48 cells.

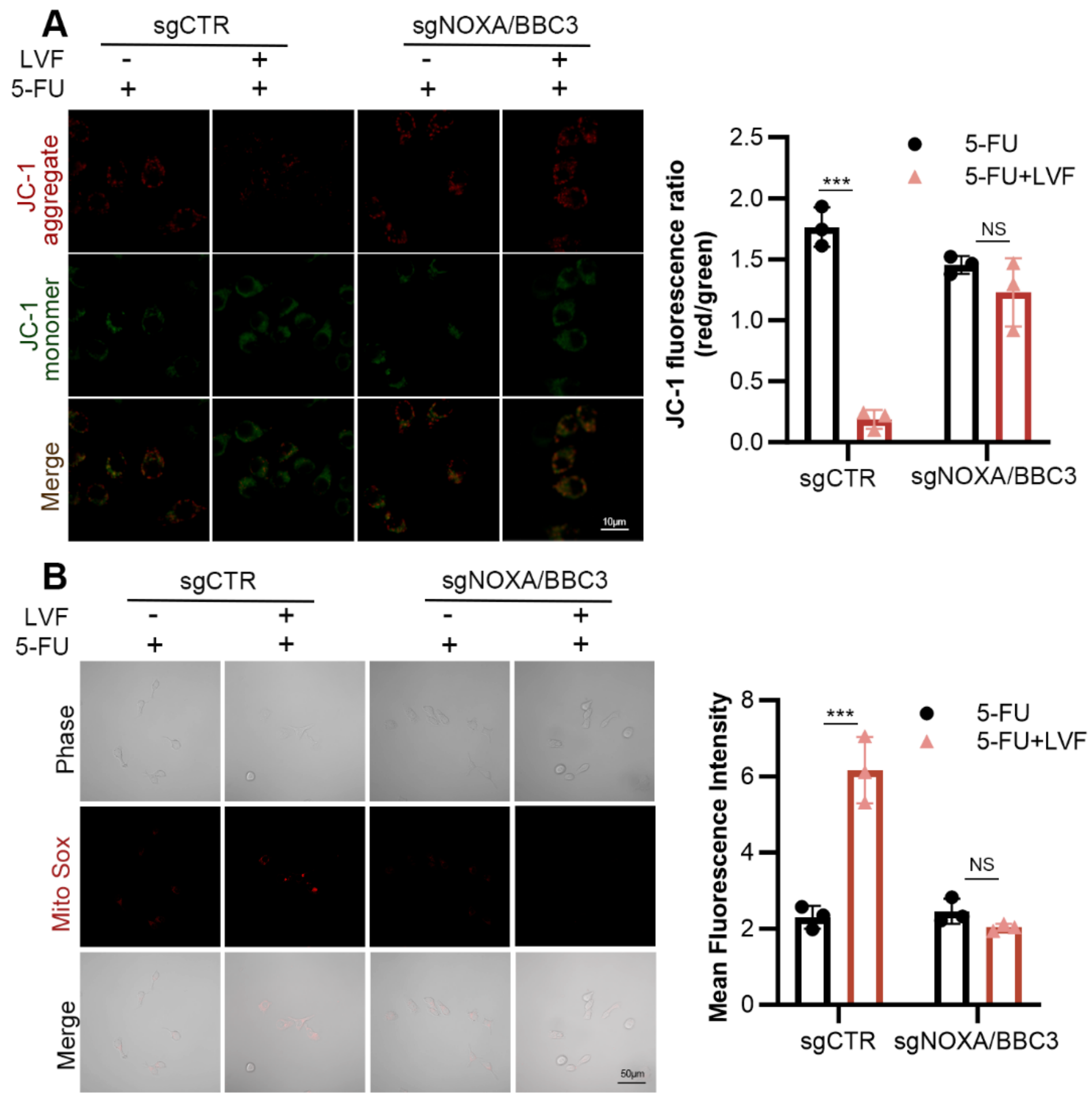

Fig. S12 Mitochondrial membrane potential and ROS in control and NOXA/BBC3-double-

knockout (DKO) SW48 cells. (**A**) Mitochondrial membrane potential (ΔΨm) detected by JC-1 staining in sgCTR and sgDKO SW48 cells treated with 5-FU or LVF + 5-FU. (**B**) Mitochondrial ROS levels measured by Mito SOX staining in sgCTR and sgDKO SW48 cells treated with 5-FU or LVF + 5-FU. Data are presented as means ± SD from three independent experiments with three technical replicates each. Statistical comparisons were performed using one-way ANOVA. *$p < 0.05$, **$p < 0.01$, ***$p < 0.001$.

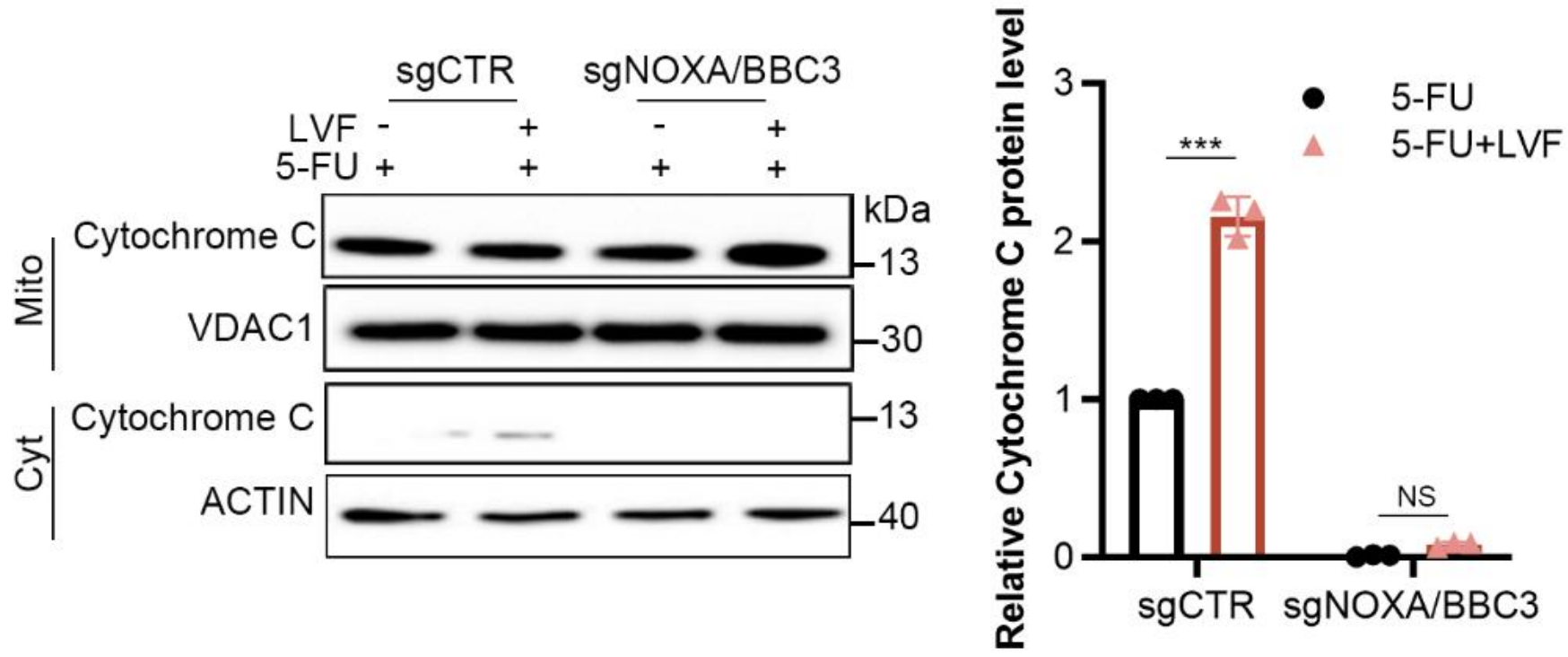

Fig. S13 Western blot analysis of cytochrome c release in control and NOXA/BBC3-double-knockout (DKO) SW48 cells. Cytoplasmic fractions were isolated from sgCTR and sgDKO SW48 cells treated with 5-FU or LVF + 5-FU, and cytochrome c levels were detected by Western blot. Data are presented as means ± SD from three independent experiments with three technical replicates each. Statistical comparisons were performed using one-way ANOVA. *$p < 0.05$, **$p < 0.01$, ***$p < 0.001$.

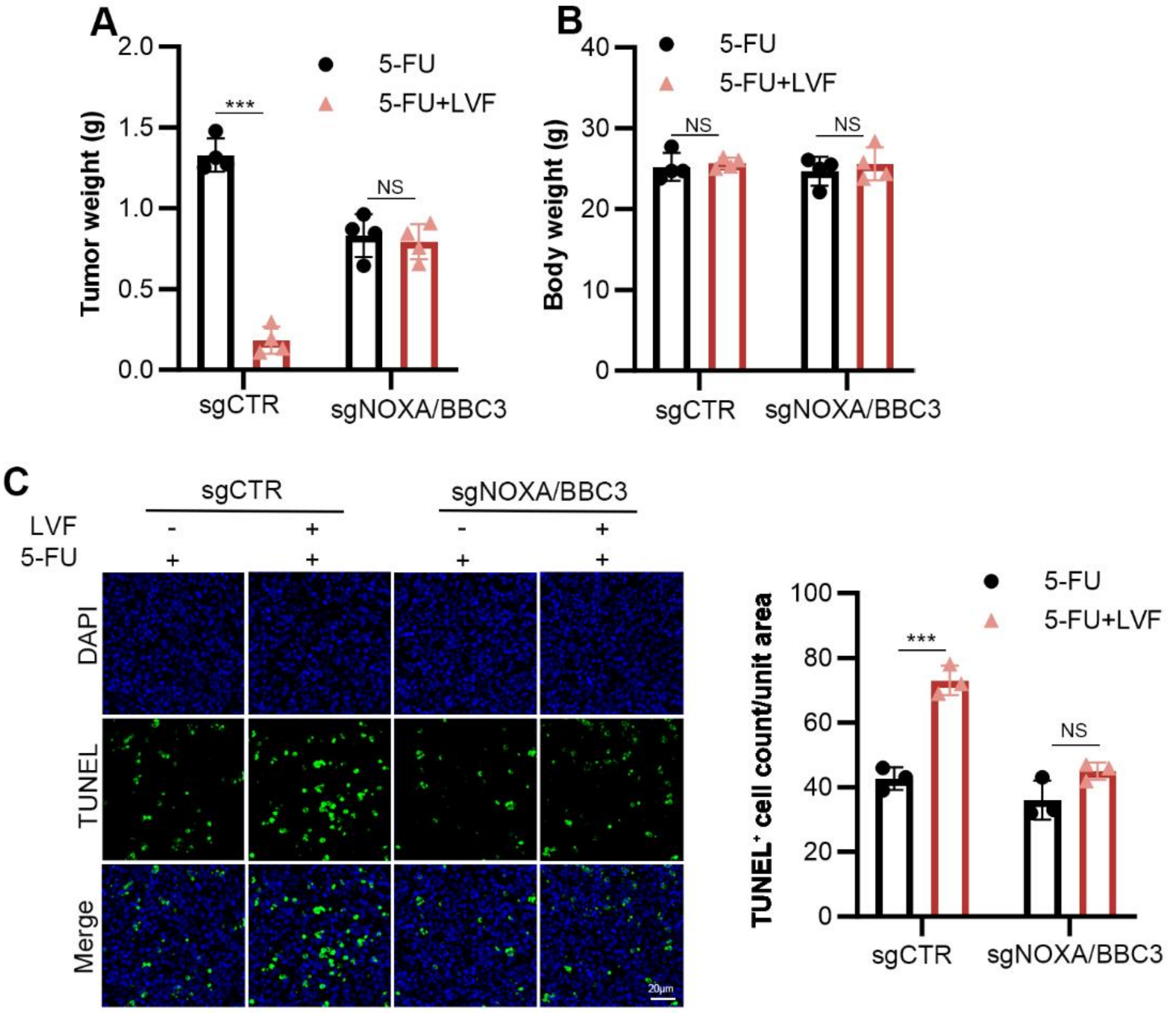

Fig. S14 Tumor weight, body weight, and TUNEL staining in NOXA/BBC3-double-knockout (DKO) SW48 xenografts. (**A**) Tumor weight measurements at endpoint from control (sgCTR) and NOXA/BBC3-DKO (sgDKO) SW48 xenografts treated with vehicle, 5-FU, or LVF + 5-FU. (**B**) Body weight measurements of mice bearing control and NOXA/BBC3-DKO SW48 xenografts monitored throughout the 4-week treatment period. (**C**) Representative TUNEL staining images of tumor sections from control and NOXA/BBC3-DKO SW48 xenografts treated with 5-FU or LVF + 5-FU. Scale bar, 100 μm. Data are presented as means ± SD. For in vivo experiments, each group contained four mice. Statistical comparisons were performed using unpaired Student's t-test or one-way ANOVA. *$p < 0.05$, **$p < 0.01$, ***$p < 0.001$.

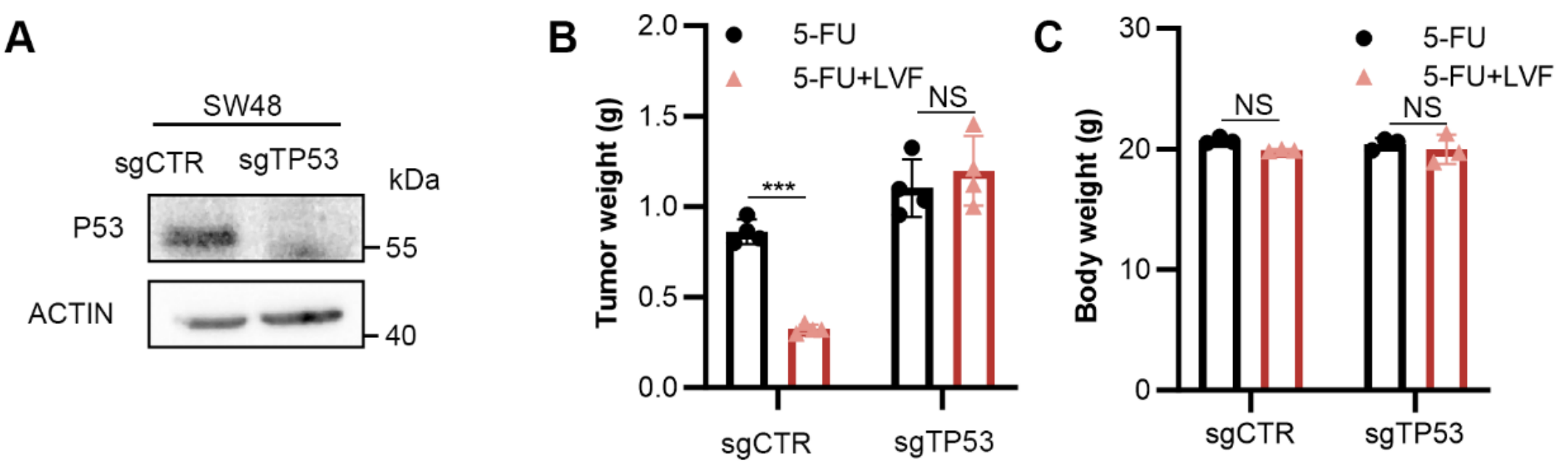

Fig. S15 TP53 protein validation and xenograft data in TP53-knockout SW48 cells. (**A**) Western blot analysis of TP53 protein levels in control (sgCTR) and TP53-knockout (sgTP53) SW48 cells. (**B**) Tumor weight measurements at endpoint from control and TP53-knockout SW48 xenografts treated with vehicle, 5-FU, or LVF + 5-FU. (**C**) Body weight measurements of mice bearing control and TP53-knockout SW48 xenografts monitored throughout the 4-week treatment period. Data are presented as means ± SD. For in vivo experiments, each group contained four mice. Statistical comparisons were performed using unpaired Student's t-test or one-way ANOVA. *$p < 0.05$, **$p < 0.01$, ***$p < 0.001$.

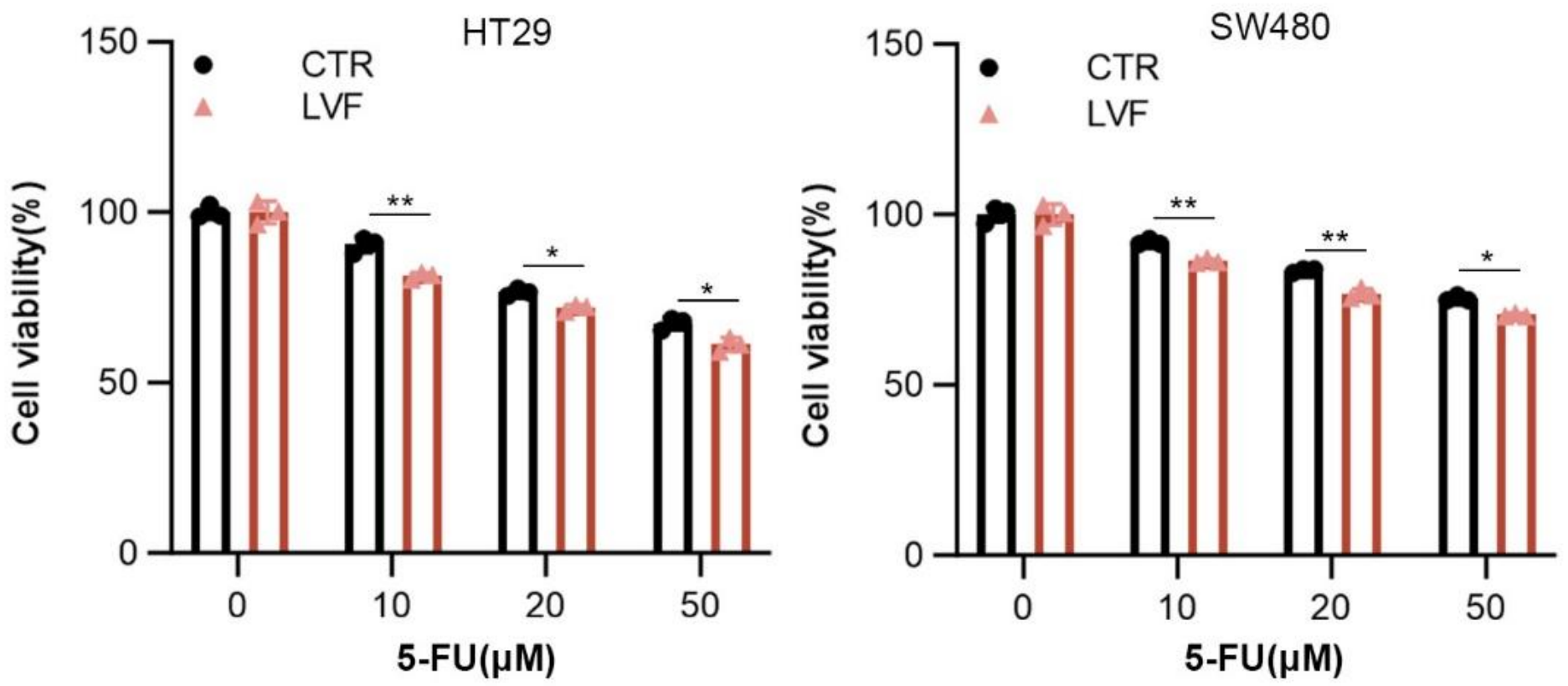

Fig S16 CCK-8 assay of cell viability in TP53-mutant CRC cell lines. HT29 (left) and SW480 (right) cells were treated with 5-FU alone (0, 10, 20, or 50 μM) or pretreated with LVF followed by co-treatment with 5-FU and LVF. Cell viability was measured by CCK-8 assay. Data are presented as means ± SD from three independent experiments with three technical replicates each. Statistical comparisons were performed using one-way ANOVA.

*p < 0.05, **p < 0.01, ***p < 0.001.

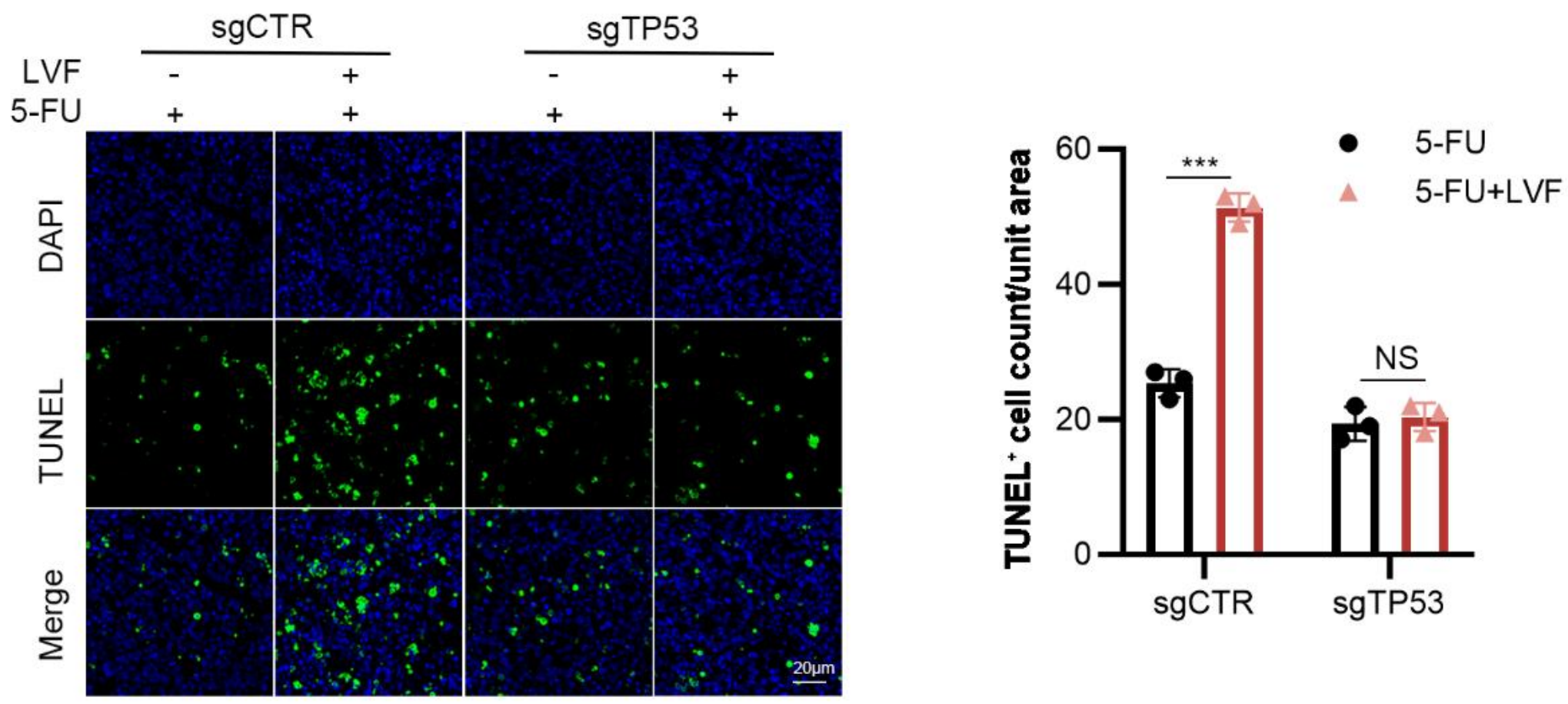

Fig. S17 TUNEL staining of tumor sections from control and TP53-knockout SW48 xenografts. Representative TUNEL staining images and quantitative analysis of apoptotic cells in sgCTR and sgTP53 SW48 xenograft tumors treated with 5-FU or LVF + 5-FU. Scale bar, 50 μm. Data are presented as means ± SD. Each group contained four mice. Statistical comparisons were performed using unpaired Student's t-test or one-way ANOVA. *p < 0.05, **p < 0.01, ***p < 0.001.

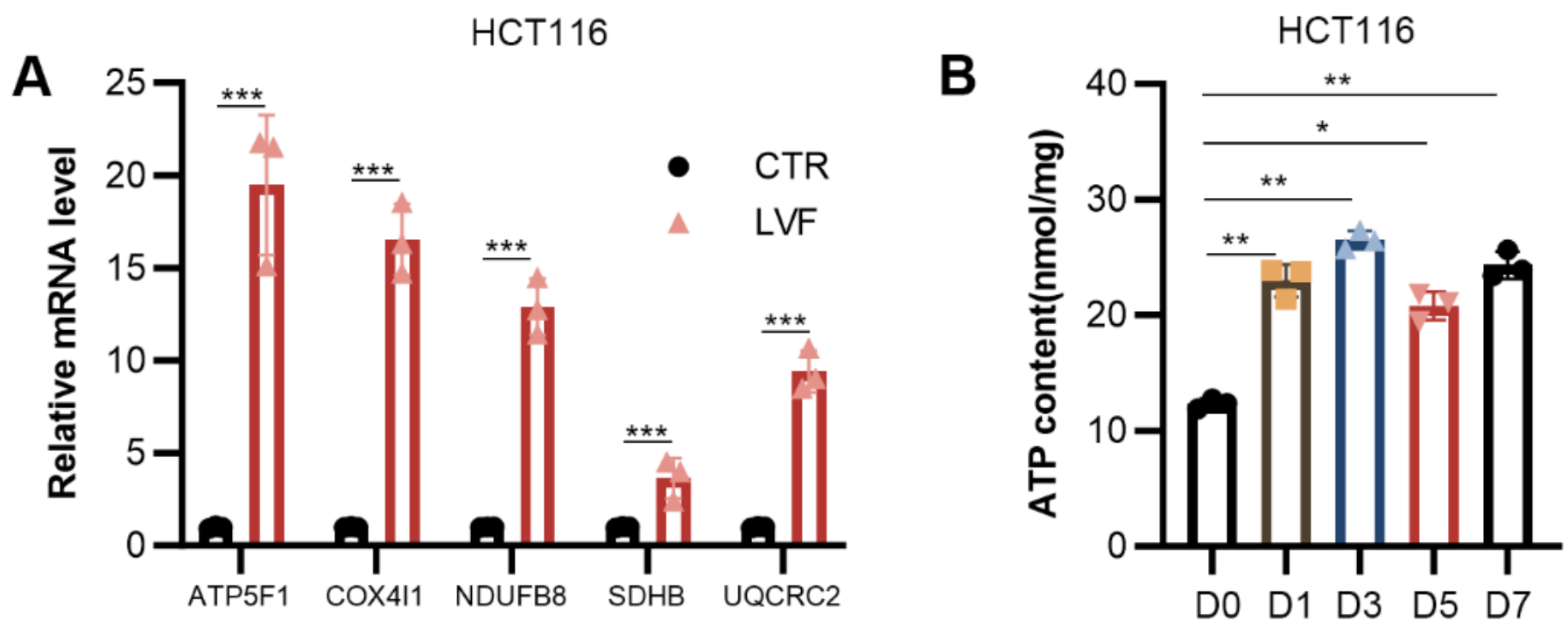

Fig. S18 OXPHOS mRNA levels and ATP content in LVF-treated HCT116 cells. (**A**) qPCR analysis of OXPHOS subunit mRNA levels (ATP5F1, COX4I1, NDUFB8, SDHB, and UQCRC2) in HCT116 cells treated with LVF (100 μM) for 5 days. (**B**) Cellular ATP content in HCT116 cells treated with LVF (100 μM) measured at indicated time points over 7 days. Data are presented as means ± SD from three independent experiments with three technical replicates each. Statistical comparisons were performed using one-way ANOVA (A) or unpaired Student's t-test (B). *p < 0.05, **p < 0.01, ***p < 0.001.

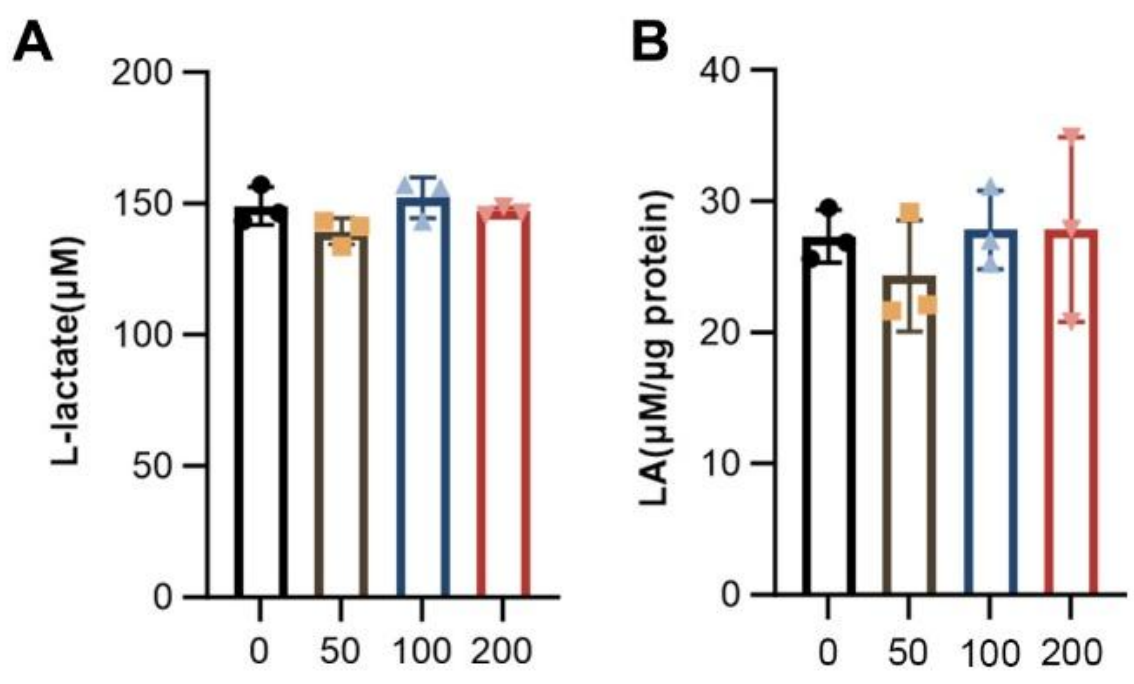

Fig. S19 Lactate production in LVF-treated SW48 cells. (**A**) Extracellular L-lactate levels in culture supernatants of SW48 cells treated with LVF (0, 50, 100, or 200 μM) for 48 hours. (**B**) Intracellular lactate content normalized to total protein in SW48 cells treated with LVF (0, 50, 100, or 200 μM) for 48 hours. Data are presented as means ± SD from three independent experiments with three technical replicates each. Statistical comparisons were performed using one-way ANOVA.

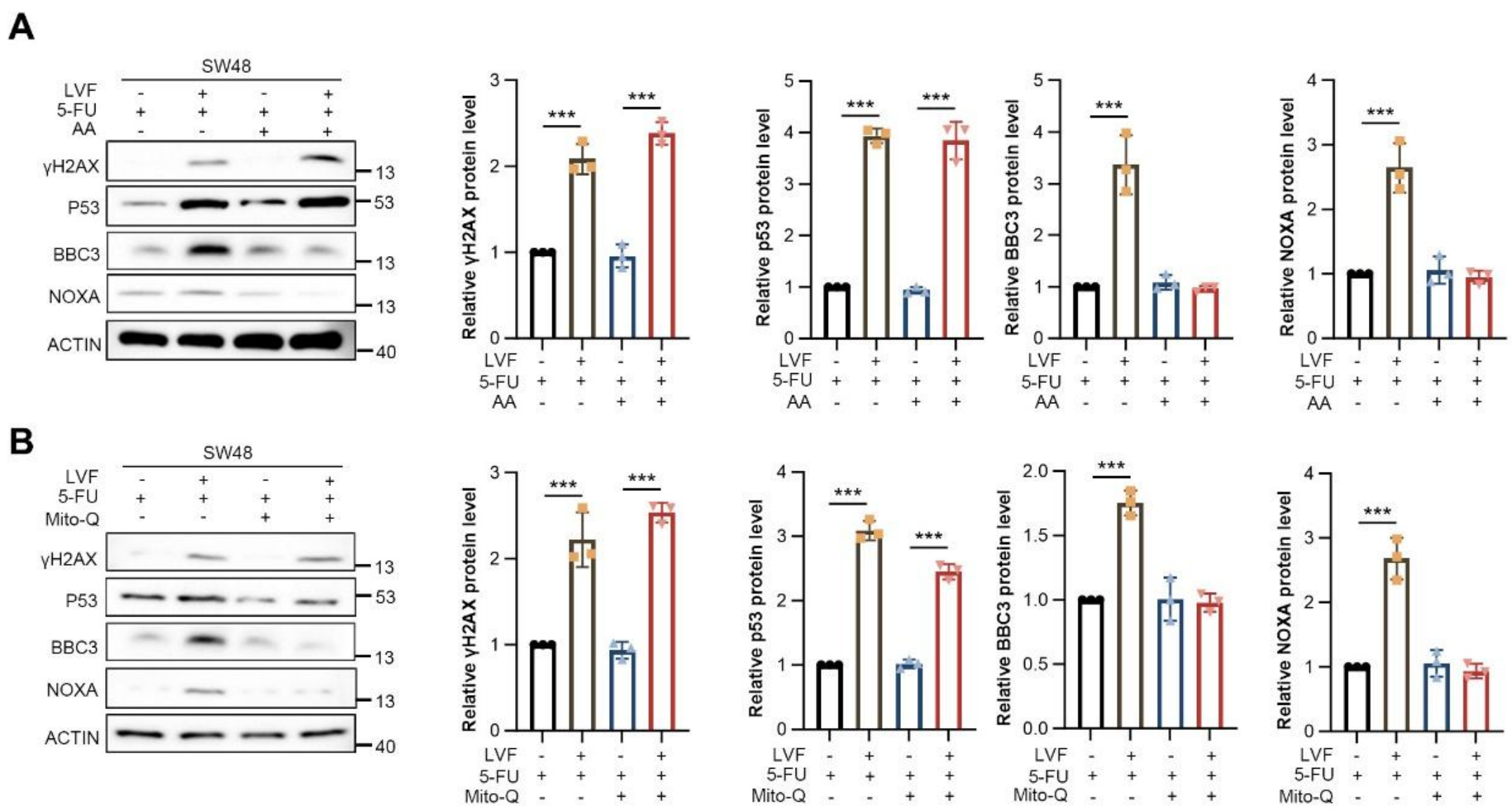

Fig. S20 Western blot analysis of DDR-TP53-mitochondrial apoptosis axis markers in rescue experiments. (**A**) SW48 cells were pretreated with LVF (100 μM) for 48 hours, followed by co-treatment with 5-FU (50 μM) and/or antimycin A (AA, Complex III inhibitor) for an additional 48 hours. Western blot analysis of γH2AX, P53, BBC3, and NOXA protein levels, with corresponding quantitative densitometry. (**B**) SW48 cells were pretreated with LVF (100 μM) for 48 hours, followed by co-treatment with 5-FU (50 μM) and/or Mito-Q (mitochondria-targeted ROS scavenger) for an additional 48 hours. Western blot analysis

of γH2AX, P53, BBC3, and NOXA protein levels, with corresponding quantitative densitometry. Data are presented as means ± SD from three independent experiments with three technical replicates each. Statistical comparisons were performed using one-way ANOVA. *$p < 0.05$, **$p < 0.01$, ***$p < 0.001$.

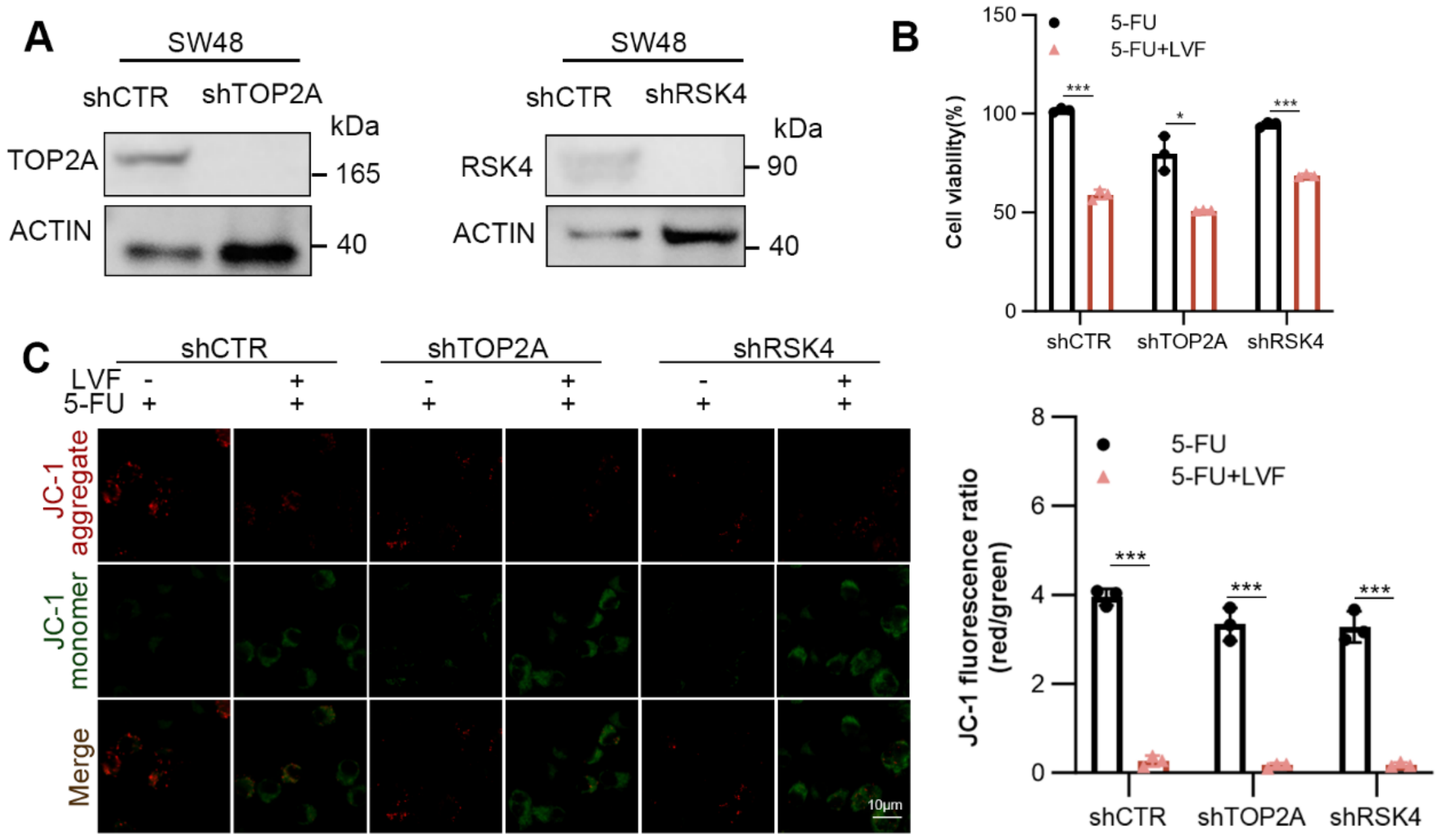

Fig. S21 TOP2A/RSK4 knockdown validation and functional assessment. (**A**) Western blot analysis of TOP2A and RSK4 protein levels in control (shCTR), TOP2A-knockdown (shTOP2A), and RSK4-knockdown (shRSK4) SW48 cells. (**B**) CCK-8 assay evaluating cell viability of shCTR, shTOP2A, and shRSK4 SW48 cells treated with 5-FU alone or 5-FU combined with LVF. (**C**) Mitochondrial membrane potential (ΔΨm) detected by JC-1 staining in shCTR, shTOP2A, and shRSK4 SW48 cells upon 5-FU + LVF treatment. Data are presented as means ± SD from three independent experiments with three technical replicates each. Statistical comparisons were performed using one-way ANOVA. *$p < 0.05$, **$p < 0.01$, ***$p < 0.001$.

Table S1. The primer sequences used for qPCR.

GENE	Forward primer	Reverse primer
**AIFM2**	AATGAGGTTTCCAGCCAGCA	GGCCACTTGGGAGTGAATGA
**BAD**	AGAGCATGTTCCAGATCCCA	GCGATGATGCTTGCCGGAG

	G	
**BAK1**	TCATCGGGGACGACATCAAC	ATAGGCATTCTCTGCCGTGG
**BAX**	GACGGCCTCCTCTCCTACTT	AAGACCACTCTTCCCCACAC
**BBC3**	GAGCAGCACCTGGAGTCG	CTCGTACTGTGCGTTGAGGT
**BID**	AGCTGCAGACTGATGGCAAC	CTACGGTCCATGCTGTCCC
**CCND1**	GAGGAACAGAAGTGCGAGGAG	GGATGGAGTTGTCGGTGTAGAT
**CD82**	CAGTGATCCTGGGCTTCGG	CACCTGGGCAATGAGGATCA
**CDKN1A**	AGGCGGTTATGAAATTCACCC	GCCCCTTCAAAGTGCCATC
**CTSD**	GACATCCACTATGGCTCGGG	AGCACGTTGTTGACGGAGAT
**CTSL**	ACTGCACAATCAGGAATACAGG	AAAAGCCCAACAAGAACCAC
**DDIT3**	GCAGCTGAGTCATTGCCTTT	CAGGTCCTCATACCAGGCTTC
**DIABLO**	TCATAGGAGCCAGAGCTGAGA	AGACCAACTGCAGTCATCCAA
**HRAS**	GGGTGAAGGACTCGGATGAC	TTGGCCGAGGTCTCGATGTA
**LMNA**	GCTGAGAGGAACAGCAACCT	TGCCAGCTGCTTCTGGAG
**NOXA**	CTGGAAGTCGAGTGTGCTACT	TCAGGTTCCTGAGCAGAAGAG
**PARP3**	GGTACCATCCAGGCTGCTTT	TTCGCCACTTCATCCACTCC
**PTPN13**	GTCAAGACCAAGAGAGGCCC	CTTTGCTTTGCAGTCCCCAC
**SERPINE1**	AGCCACTGGAAAGGCAACAT	GTCGACTTCAGTCTCCAGGG
**SESN2**	CCCAGCGCCTTCATCCC	CCAGGTTGTCTACTCGCCC
**SIVA1**	GGTAGCGTCCATTGCCTGTT	GCTCACACTGACCGCAGAC
**TNFRSF10A**	CAGCAGACCGAGTGGCAG	AGCGCAGGTAATCCCAAAAG
**TNFRSF10B**	GTACAAAGCACAGTGGGGAAG	CCACAATCAAGACTACGGCTG
**TUBA4A**	CCGAAATGGCCCATACCGAC	ACCACGGGCATAGTTGTTGG
**H-ACTIN-F**	GTCCACCGCAAATGCTTCTA	TGCTGTCACCTTCACCGTTC
**NDUFB8-F**	CCTGTGTGTAGGCACCACTT	TAGGAAGGCAGGAACAGCAC
**SDHB-F**	AGGCATCTGTGGCTCTTGTG	AGTTGCTCAAATCCTTATTGAGGT
**UQCRC2-F**	CCTGCGGGGTGATGTTGATA	CAGCTACTTCCCAACGACGA
**COX4I1-F**	CTCTTCCGGTCGCGGG	AAAAATGTACACCTGCCGCC
**ATP5F1A-F**	CAGTCTACGCCGCACTTACT	CATCTCAGCAGTCCCACAGT

DATA S1. Original western blot membranes

Figure 2H

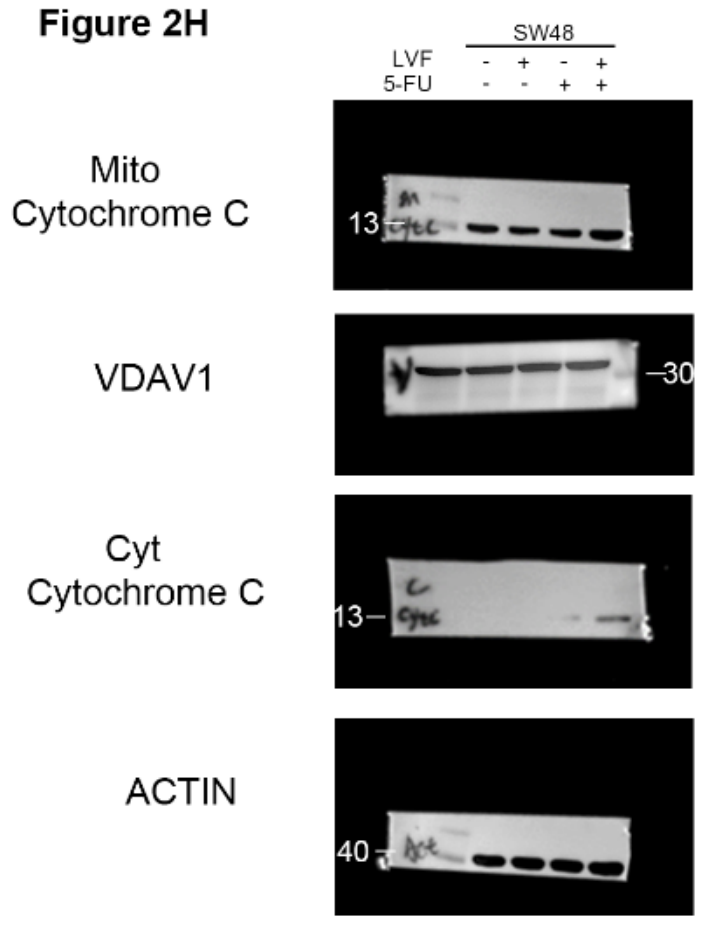

Figure 3B

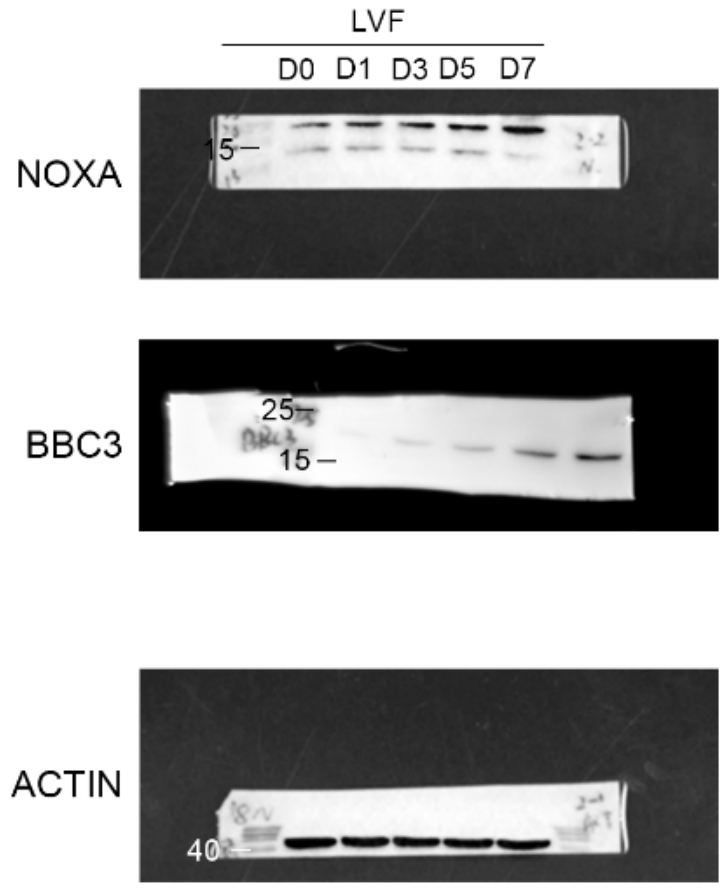

Figure 3C

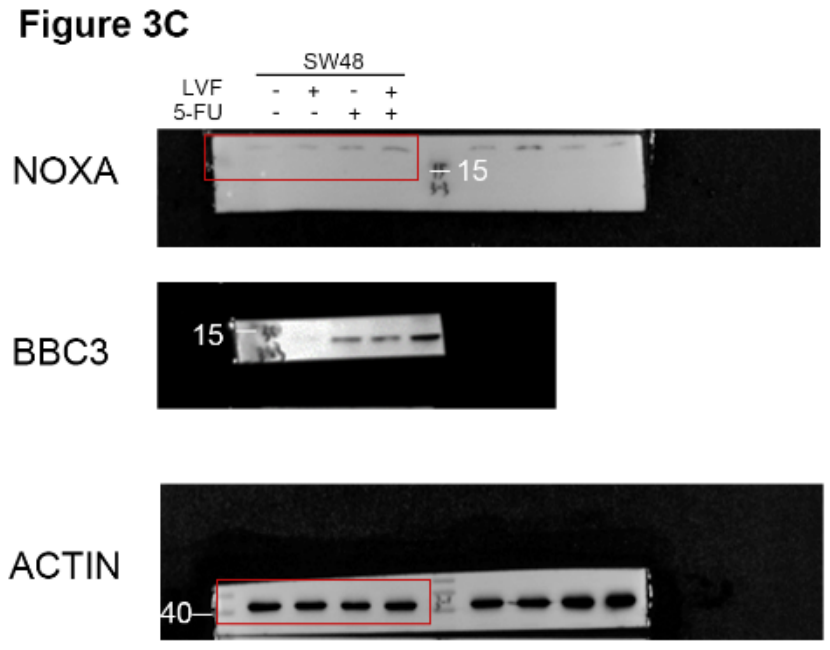

Figure 4A

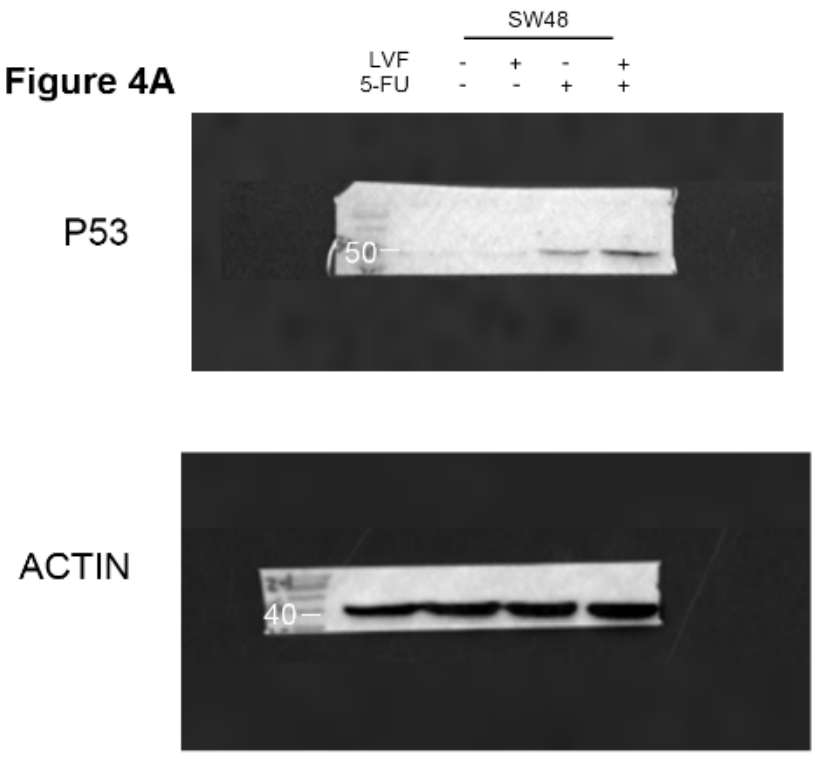

Figure 4B

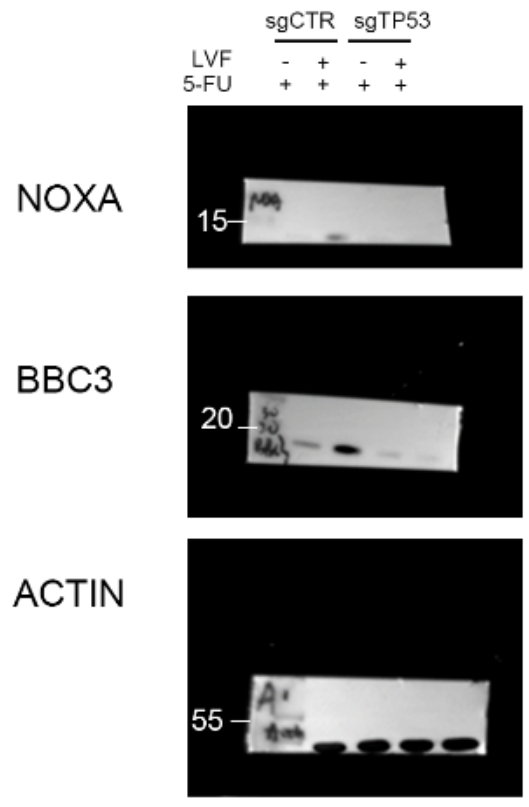

Figure 5B

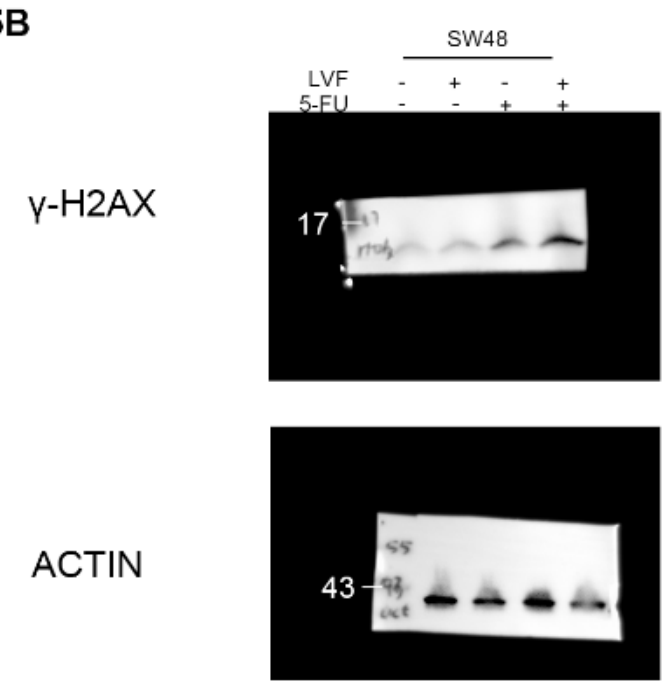

Figure 6J

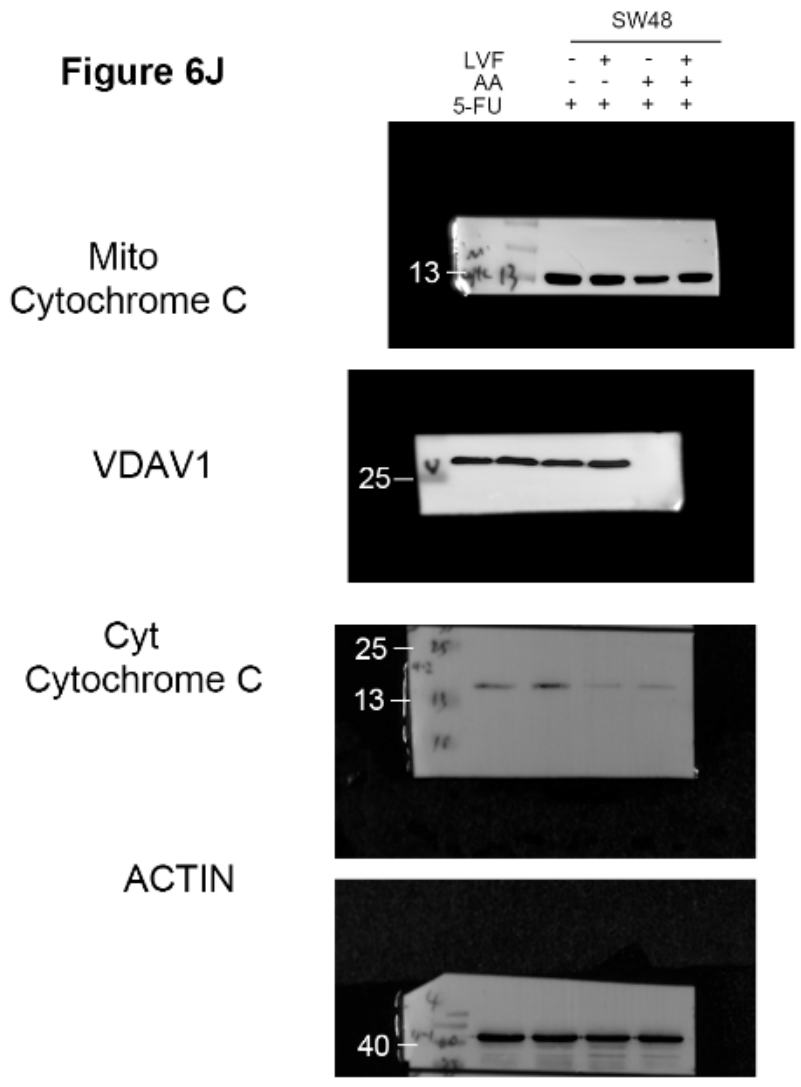

Figure 7B

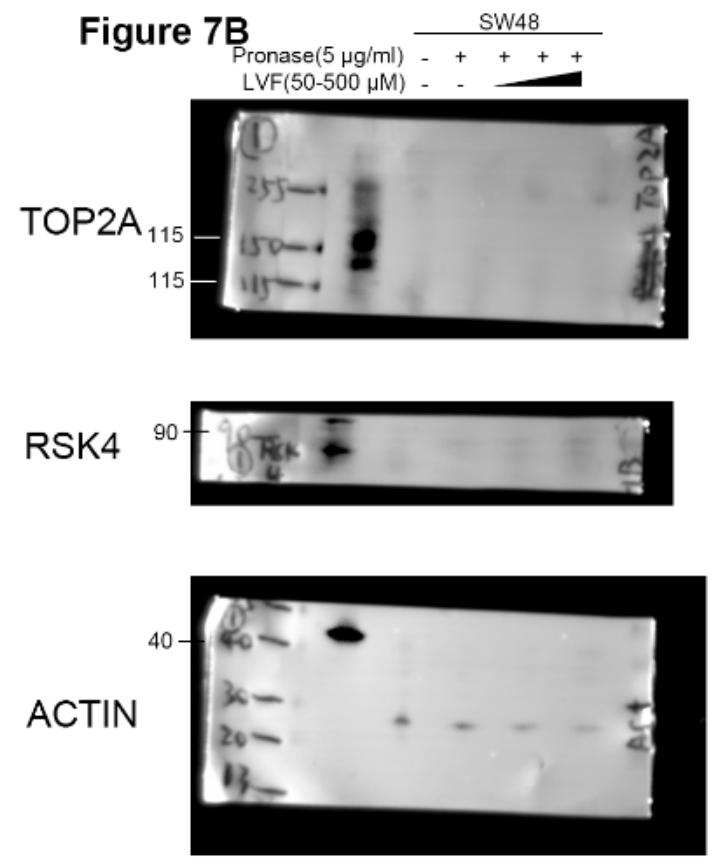

Figure 7G

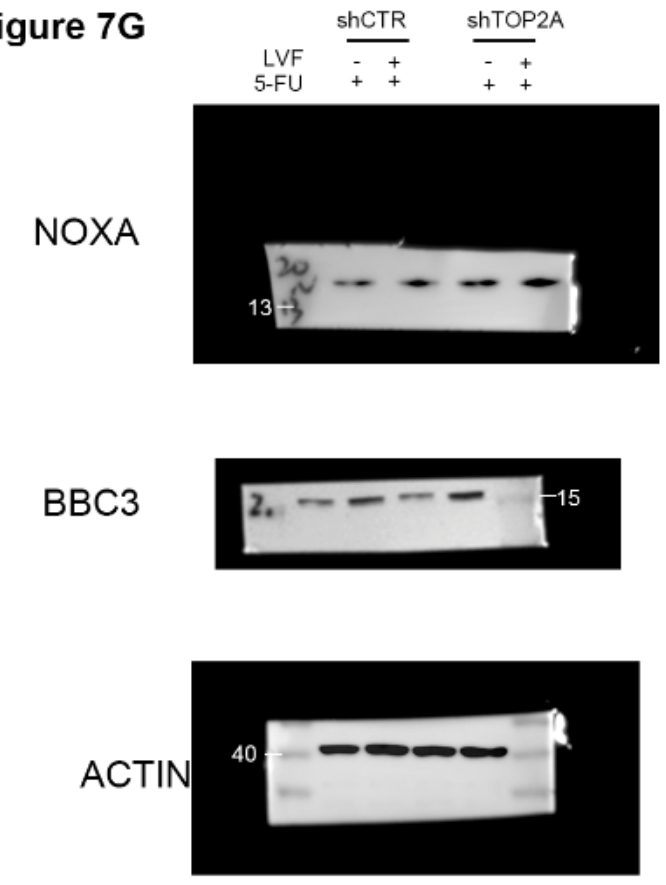

Figure 7H

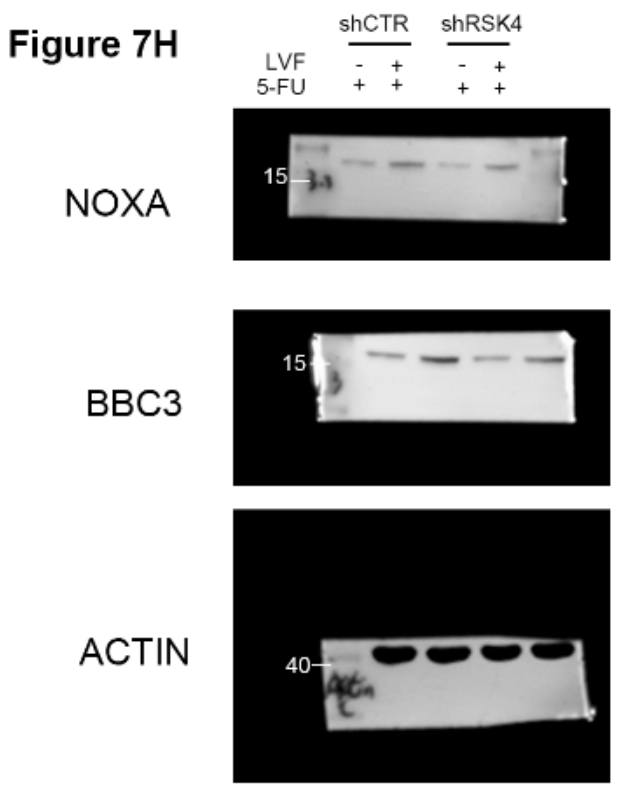

Figure S1F

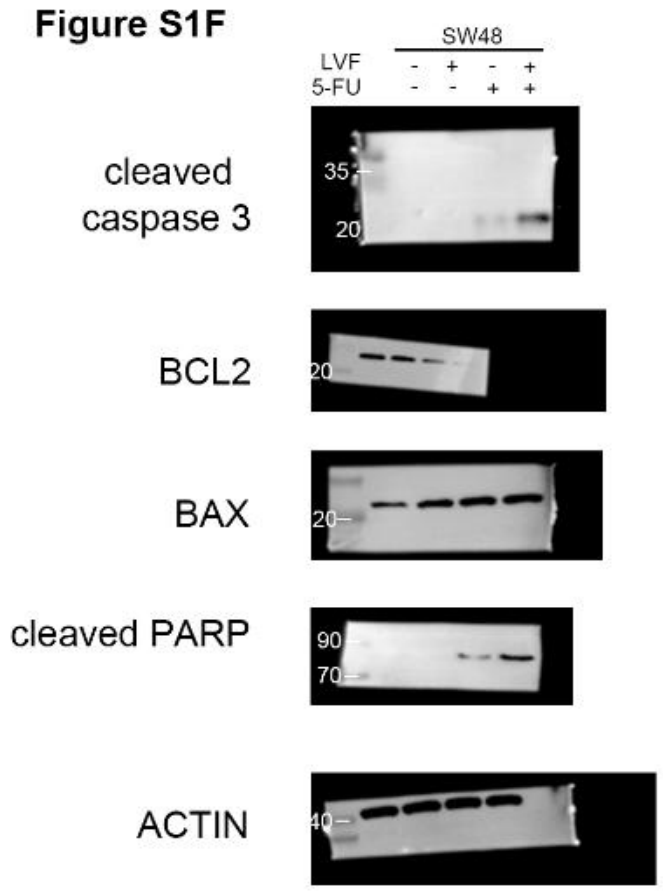

Figure S1G

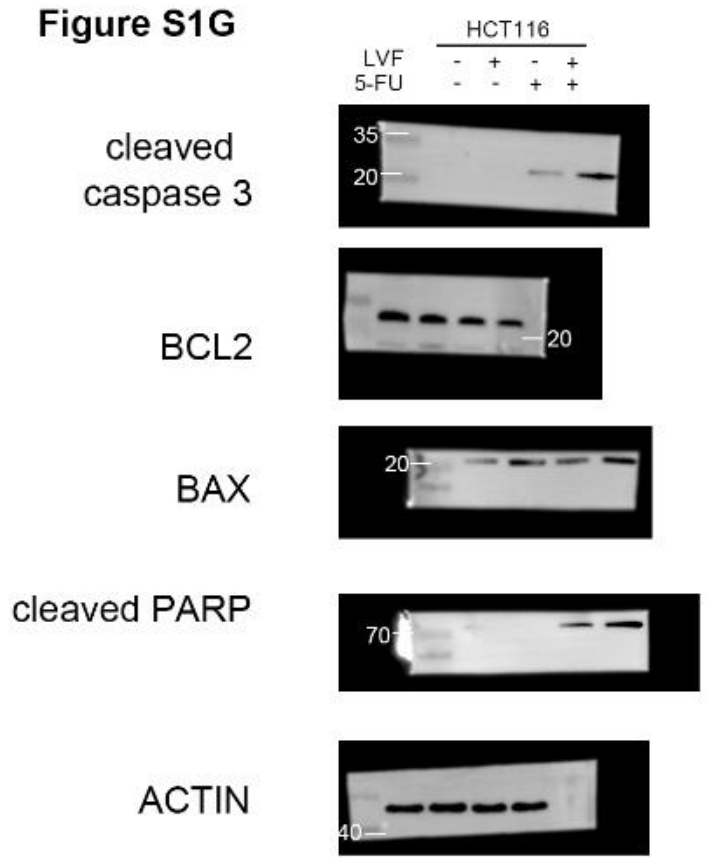

Figure S10A

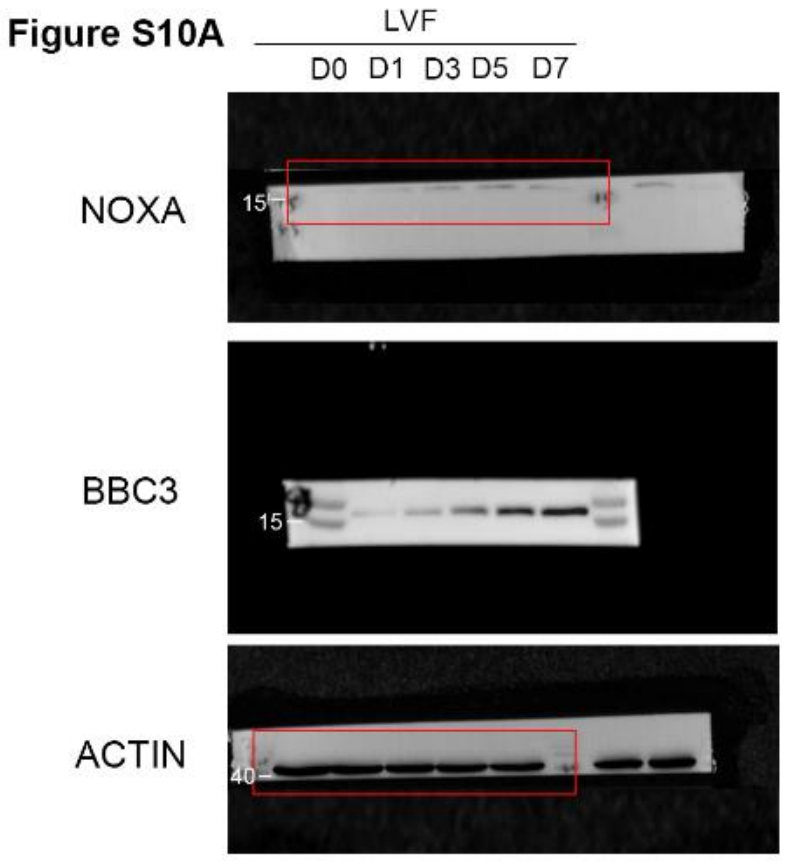

Figure S10B

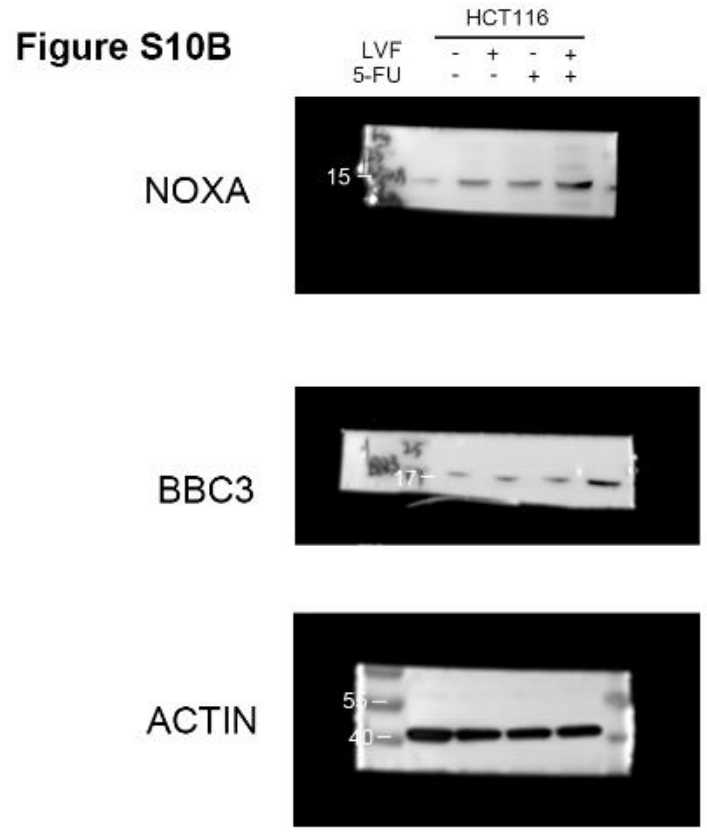

Figure S11A

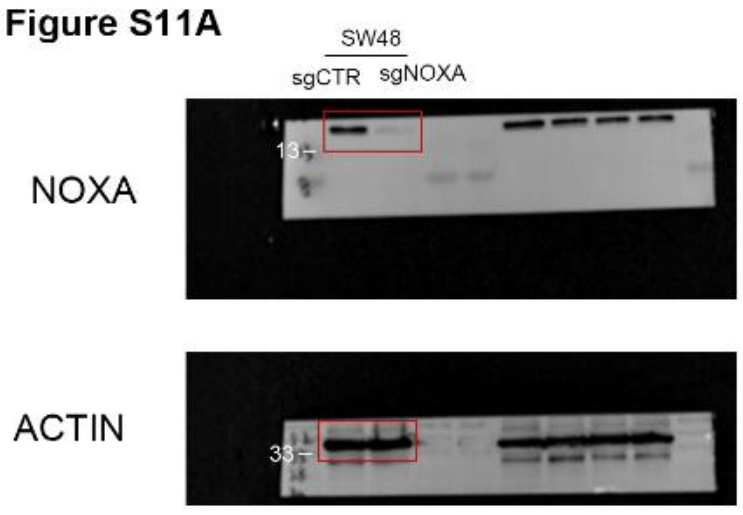

Figure S11B

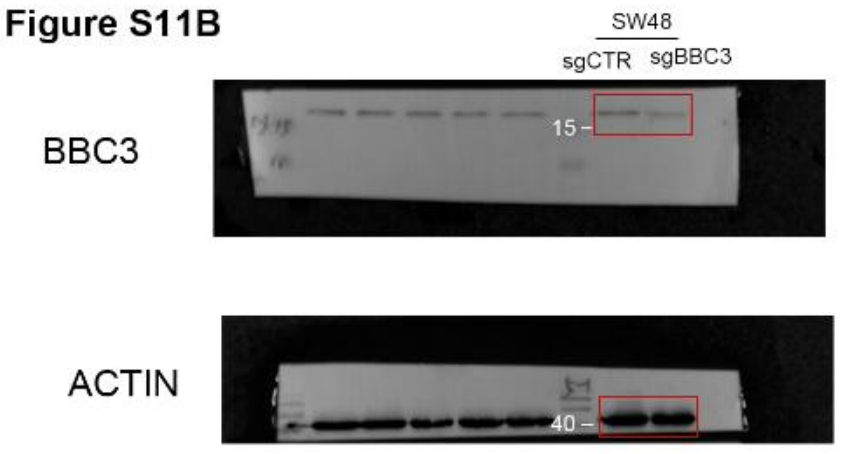

Figure S11C

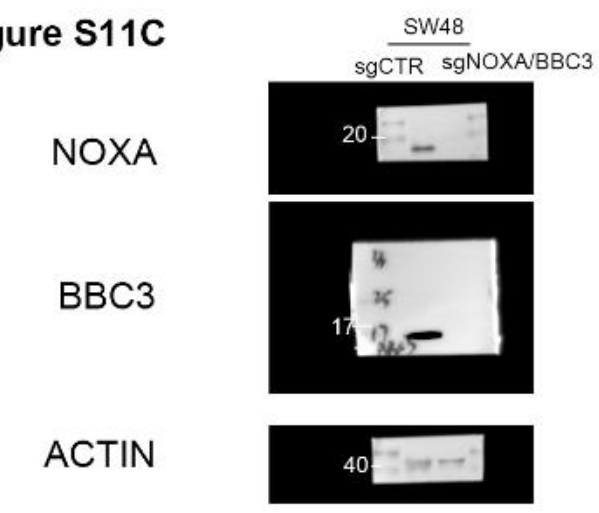

Figure S13

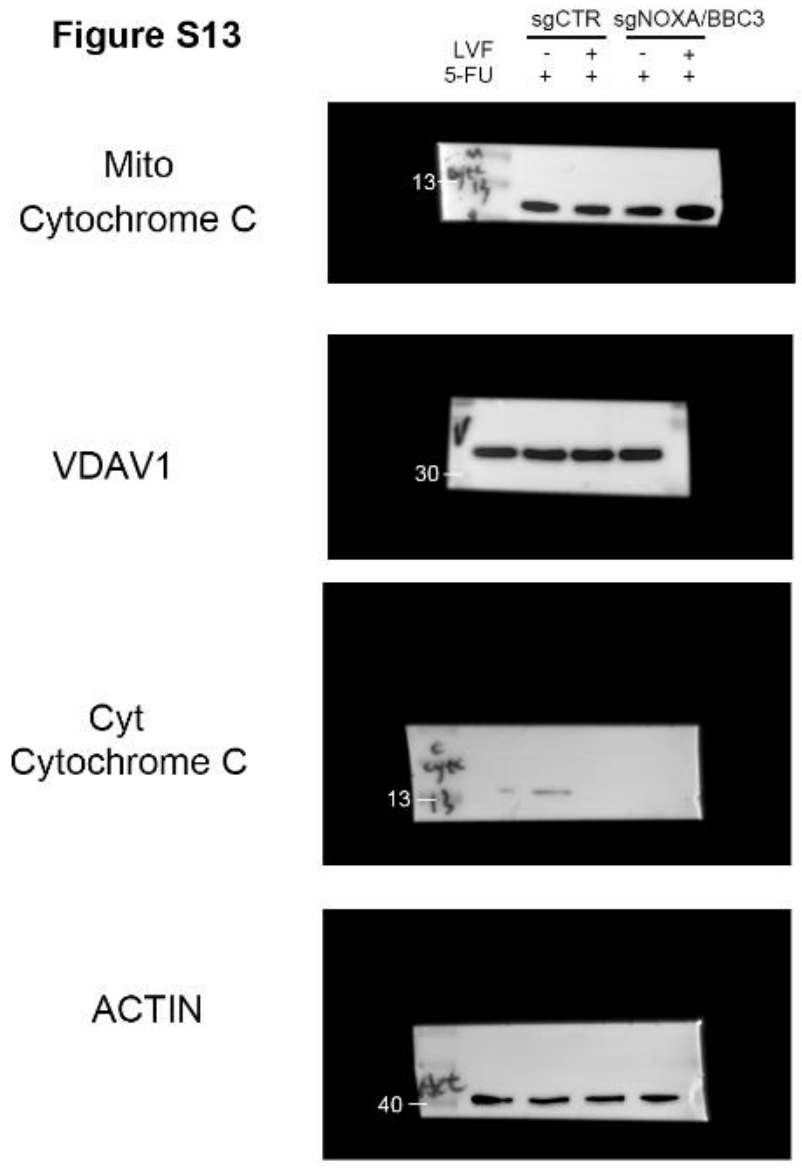

Figure S15

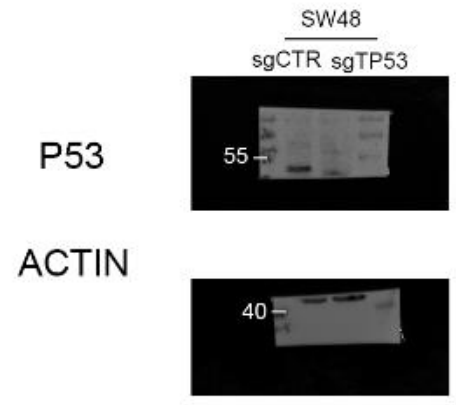

Figure S20A

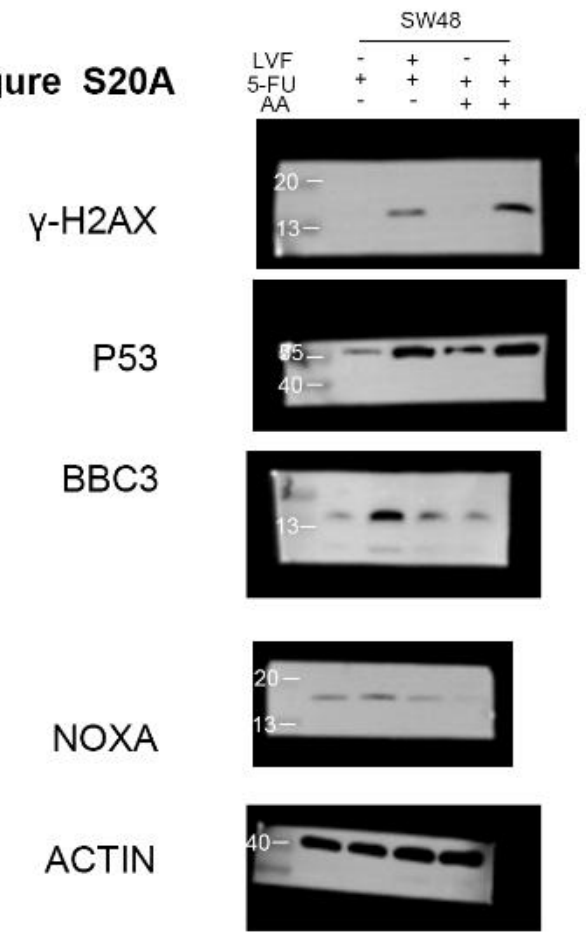

Figure S20B

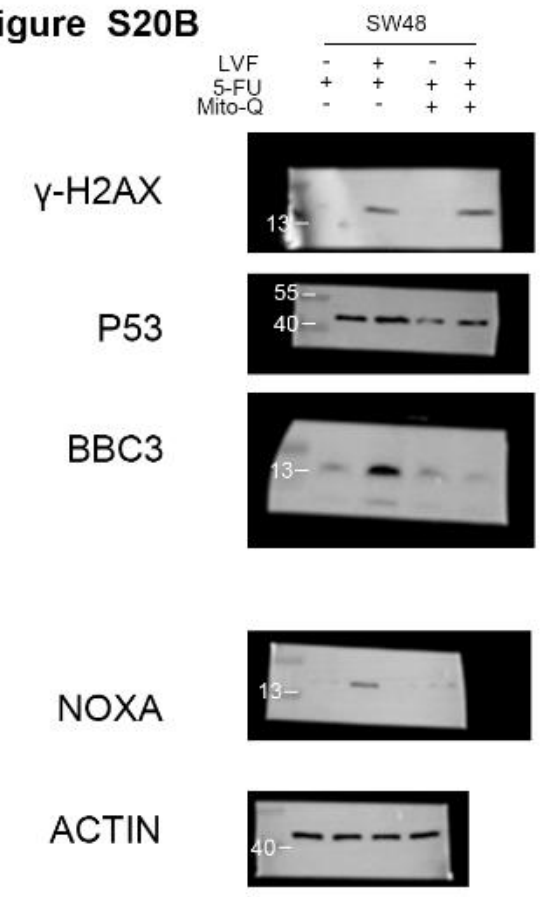

Figure S21

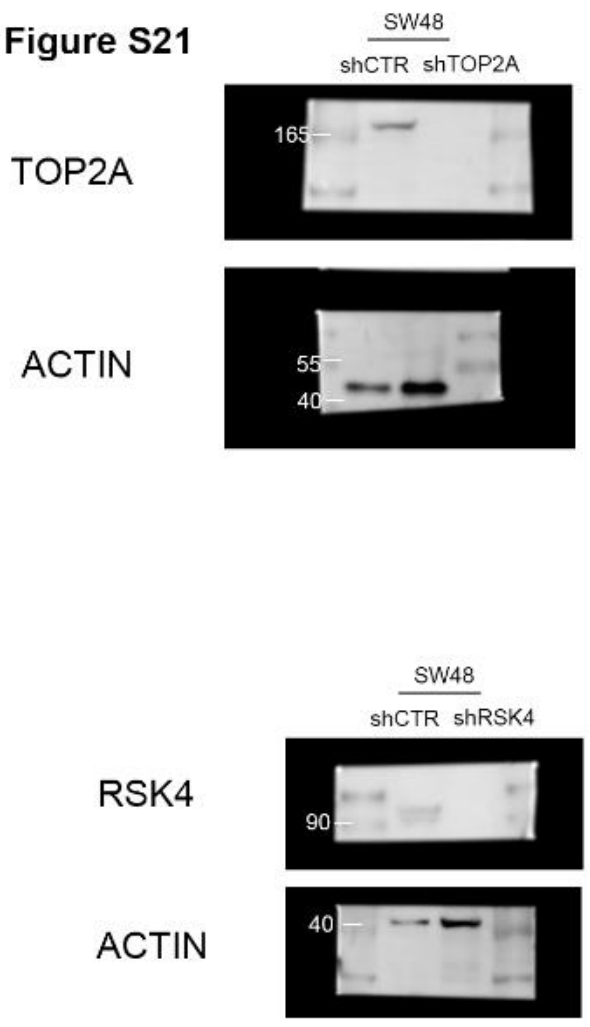

Supplement: Supplementary file 1 [file mmc1.pdf]
